# Repurposing anthelmintic agents to eradicate resistant leukemia

**DOI:** 10.1038/s41408-020-0339-9

**Published:** 2020-06-26

**Authors:** Caterina Mezzatesta, Liridon Abduli, Anna Guinot, Cornelia Eckert, Denis Schewe, Marketa Zaliova, Luciana Vinti, Blerim Marovca, Yi-Chien Tsai, Silvia Jenni, Julia Aguade-Gorgorio, Arend von Stackelberg, Martin Schrappe, Franco Locatelli, Martin Stanulla, Gunnar Cario, Jean-Pierre Bourquin, Beat C. Bornhauser

**Affiliations:** 1 Department of Oncology and Children’s Research Center, Children’s Hospital Zurich, Lengghalde 5, Balgrist Campus AG, 8008 Zurich, Switzerland; 2 Department of Pediatric Oncology/Hematology, Charité-Universitätsmedizin Berlin, Campus Virchow Klinikum, Berlin, Germany; 3 German Cancer Consortium (DKTK), Berlin, Germany; 4 Department of Pediatrics, University Hospital Schleswig-Holstein, Kiel, Germany; 5 Department of Pediatric Hematology and Oncology, Second Faculty of Medicine, Charles University and University Hospital Motol, Prague, Czech Republic; 6 Department of Pediatric Haemato-Oncology, IRCCS Ospedale Pediatrico Bambino Gesù, Sapienza University of Rome, Rome, Italy; 7 Pediatric Hematology and Oncology, Hannover Medical School, Hannover, Germany

**Keywords:** Acute lymphocytic leukaemia, Translational research

## Abstract

Despite rapid progress in genomic profiling in acute lymphoblastic leukemia (ALL), identification of actionable targets and prediction of response to drugs remains challenging. To identify specific vulnerabilities in ALL, we performed a drug screen using primary human ALL samples cultured in a model of the bone marrow microenvironment combined with high content image analysis. Among the 2487 FDA-approved compounds tested, anthelmintic agents of the class of macrocyclic lactones exhibited potent anti-leukemia activity, similar to the already known anti-leukemia agents currently used in induction chemotherapy. Ex vivo validation in 55 primary ALL samples of both precursor B cell and T-ALL including refractory relapse cases confirmed strong anti-leukemia activity with IC_50_ values in the low micromolar range. Anthelmintic agents increased intracellular chloride levels in primary leukemia cells, inducing mitochondrial outer membrane depolarization and cell death. Supporting the notion that simultaneously targeting cell death machineries at different angles may enhance the cell death response, combination of anthelmintic agents with the BCL-2 antagonist navitoclax or with the chemotherapeutic agent dexamethasone showed synergistic activity in primary ALL. These data reveal anti-leukemia activity of anthelmintic agents and support exploiting drug repurposing strategies to identify so far unrecognized anti-cancer agents with potential to eradicate even refractory leukemia.

## Introduction

Intensive chemotherapy regimens in childhood lymphoblastic leukemia (ALL) have led to substantial improvement in survival, which now overcomes 80%^1,2^. Despite these advances, relapsed leukemia rank among the most frequent diagnoses in childhood malignancies, and remain a major clinical problem, being often associated with fatal outcome^3^. While powerful new immunotherapeutic approaches are currently being developed in particular in B cell precursor ALL (BCP-ALL)^4–6^, identification of small molecules with anti-leukemic potential that could be incorporated into current treatment regimens represents an appealing possibility to increase efficacy of anti-leukemic therapy. Examples for such a strategy are modulation of survival and cell death pathways through small molecules, such as the BCL-2 mimetic venetoclax^7,8^, inhibitors of the PI3K/AKT/mTOR pathway such as Torin, or of the MEK pathway such as Trametinib^9,10^. Another strategy exploits the potential of small molecules to activate non-apoptotic cell death pathways, such as the IAP inhibitor birinapant, which we showed to activate necroptosis to potently interfere with leukemia progression^11,12^. Common to all these strategies is the need for the development of functional approaches to identify the target patient population which is most likely to respond. Compounds that are clinically used for non-cancer indications may represent an appealing source for a novel treatment approach. Repurposing potential anti-leukemic drugs for ALL treatment may accelerate clinical development, and will reduce cost and time required for the introduction in the clinic^13,14^. We performed a functional imaging-based drug repurposing screen using an extended FDA-approved drug library, which we tested on primary relapsed ALL co-cultured on mesenchymal stroma cells (MSCs) in a model of the bone marrow microenvironment^15^. Unexpectedly, we identified the anthelmintic agents moxidectin, ivermectin, and milbemycin to have a high anti-leukemic potential as single agents and to exhibit synergistic activity with the BCL-2 inhibitor navitoclax (ABT-263) and standard chemotherapeutic agents of frontline ALL therapy, such as dexamethasone in highly refractory primary ALL.

## Materials and methods

### Human and patient-derived xenograft (PDX) samples

Primary human ALL samples were obtained from cryopreserved bone marrow aspirates of patients enrolled in the AIEOP-BFM 2009 and ALL-REZ 2002 studies. Informed consent was obtained in accordance with the Declaration of Helsinki, and approval was granted by the Ethics Commission of the Kanton Zürich (approval no. 2014–0383). Samples were classified as standard risk (SR), medium risk (MR), high risk (HR), very high risk (VHR), and relapse (R) samples according to the clinical criteria used in the ALL-BFM 2000 study^16^. Primary human ALL cells were transplanted into 5- to 12-week-old immunodeficient NOD/SCID/IL2rγnull (NSG) mice in order to obtain PDX cells^17^. Leukemia progression was monitored weekly by staining peripheral blood after red blood cell lysis with hCD19-PE and hCD45-Alexa Fluor 647 (Biolegend) and analyzed by flow cytometry (Fortessa LSR, BD Biosciences). Engrafted ALL cells were collected from the spleen. In vivo experiments were approved by the veterinary office of the Canton of Zurich.

### Ex vivo drug screen

The drug screen was performed with ALL cells grown in co-culture on MSCs in 384-well plates^15^. Briefly, 2500 MSCs in 30 µL AIM-V (Thermo Fisher Scientific) medium per well were plated 24 h before adding 25,000 ALL cells in 27.5 µL of AIM-V using a Biotek EL406 microplate washer/dispenser. The drug library of 2487 FDA-approved (Nexus platform ETH) compounds at 1 µM drug concentration was applied 24 h after the seeding of ALL cells with a Tecan EVA 100 liquid handling robot (Nexus platform ETH). Treated cells were incubated at 37 °C with 5% CO_2_ and 95% humidity for 72 h. Cell viability was evaluated by CyQuant staining (Thermo Fisher Scientific) and analyzed by TTP Labtech Acumen Cellista. Specific details on antibodies and other reagents are given in Supplementary Table S1.

### Statistical analysis of ex vivo drug screen

Systematic variation from plate to plate was removed by standard normalization procedures^18^. Following common practice, assay quality was evaluated on the basis of the *Z*′ factor. To eliminate within-plate edge effects a smooth polynomial correction was applied using the Loess function^19^. Differential activity was analyzed following the workflow sketched in ref. ^20^. Briefly, for each single-dose measurement of a compound, a *Z*-test was performed against the Null hypothesis that its activity is indistinguishable from the negative controls. For this to be valid, the distribution of activities of the negative controls was checked to be normal. The mean and variance of the distribution was estimated robustly for each plate and smoothly averaged over a range of consecutive plates. The drug activity was ranked based on the percentage of the positive control idarubicin.

### Ex vivo dose response and synergy profiling

Drug sensitivity and drug synergy profiles were evaluated using ALL cells in co-culture with MSCs as described above. A Tecan D300 robot was used to dispense drugs at the indicated dilutions. ABT-263 (navitoclax) and dexamethasone (Selleckchem) concentrations for synergy were based on the half maximal inhibitory concentration (IC_50_) of the different samples screened. Cell viability was evaluated after 72 h of drug treatments using CyQuant staining and imaging-based cell viability analysis on an ImageXpress microscope (Molecular Devices; ref. ^15^). All the dose response curves and the IC_50_ were represented and calculated using GraphPad Prism 8. The synergy was calculated using SynergyFinder tool^21,22^. The resulting ZIP score indicates synergism (ZIP score ≥ 1), additivity (ZIP score 0–1) or antagonism (ZIP score ≤ 0), and is here represented by a 3D graph^21,22^.

### Generation of knockout PDX cells

Knockout PDXs of RIPK1 (RIPK1KO) and of Caspases-3 and -7 (C3/C7KO) were generated using the multicolor lentiCRISPR system^11,23^. The triple knockout containing depletion of Caspase-2 (C3/C7/C2KO) or Caspase-6 (C3/C7/C6KO) was produced transducing C3/C7KO cells with the lentiviral vector sg_shuttle_RFP657 (ref. ^24^) containing the respective sgRNAs. Transduced cells were transplanted into NSG mice for expansion, subsequently sorted and retransplanted to obtain a pure knockout population. The knockout was confirmed by western blotting. The sgRNAs used for RIPK1, Caspase-3, Caspase-7, Caspase-6, and Caspase-2 were published in refs. ^11,23,25^.

### Intracellular chloride measurement

Intracellular chloride concentration was assessed using the fluorescent indicator MQAE (Thermo Fisher Scientific). In total, 500,000 ALL cells were recovered in 300 µL AIM-V conditioned medium, which was harvested upon 24 h incubation of MSC monocultures. After 1 h of recovery, the ALL cells were treated with moxidectin (Sigma Aldrich) (1, 2, and 3 µM) or ivermectin (Selleckchem) (1 or 3 µM) for 2 or 4 h. The cells were then incubated at 37 °C for 1 h with MQAE (1 mM) and for 15 min with PI (Thermo Fisher Scientific). After staining, the cells were collected, centrifuged, and resuspended in fresh 1× phosphate-buffered saline (PBS). MQAE fluorescence was quantified in PI-negative cells by flow cytometry (Fortessa LSR, BD Biosciences). Results were analyzed by FlowJo 10.2 and statistical analysis was performed using GraphPad Prism 8 with *t*-test analysis.

### Measurement of mitochondrial outer membrane permeabilization (MOMP)

Evaluation of MOMP was done using the fluorescent dye tetramethylrhodamine ethyl-ester (TMRE) (Thermo Fisher Scientific). In total, 500,000 cryopreserved ALL cells were recovered for 1 h in 300 µL AIM-V medium. Upon recovery, ALL cells were treated with moxidectin (1, 2, or 3 µM) or ABT-263 (50, 100, or 250 nM) for 2 h and subsequently incubated at 37 °C for 15 min with TMRE (50 nM). After staining, the cells were collected, centrifuged, and resuspended in 1× PBS. TMRE fluorescence was quantified by flow cytometry (Fortessa LSR, BD Biosciences). Results were analyzed by FlowJo 10.2 and statistical analysis was performed using GraphPad Prism 8 with *t*-test analysis.

## Results

### Ex vivo drug screen identifies antiparasitic compounds as promising candidates to target refractory leukemia

**Fig. 1:**
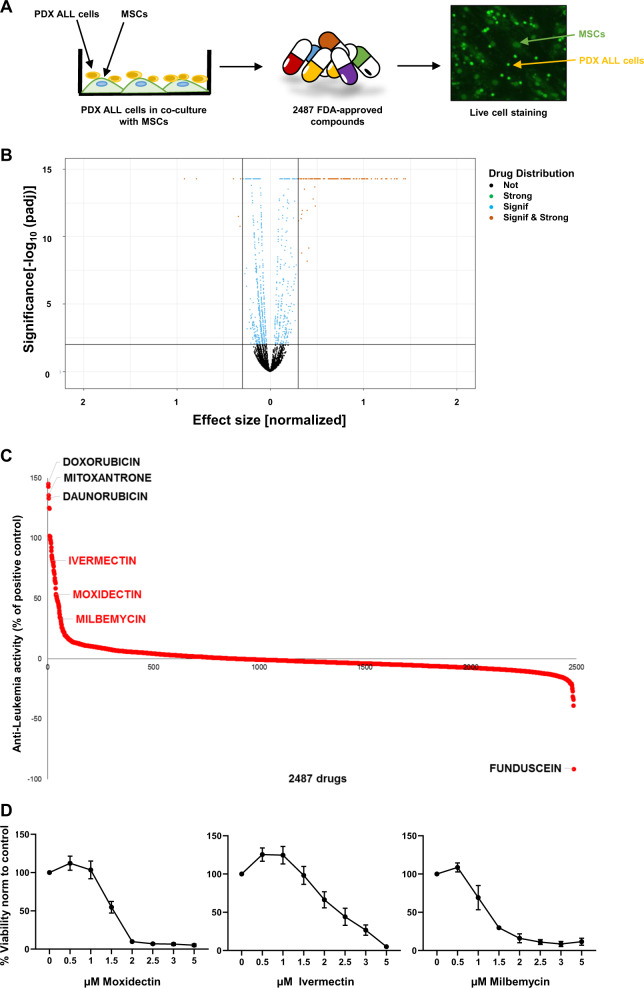
Ex vivo drug repurposing screen identifies anthelmintic agents as a new option for anti-leukemia therapy. **a** Strategy for ex vivo drug screening of patient-derived xenograft (PDX) cells in co-culture with mesenchymal stroma cells (MSCs), which were treated with 2487 compounds (each at 1 µM concentration) with FDA approval. Fluorescent live cell staining using CyQuant and automated image analysis was used to quantify living ALL^15^. **b** Volcano plot of the resulting drug activity. The horizontal line corresponds to FDR = 0.01 and compounds below this line are labeled “not significant”. The two vertical lines correspond to a change of ±0.3 in activity as compared to vehicle control and compounds outside this range are labeled “strong”. The legend refers to “not” (black)—not significant and weak effect; “signif & strong” (light brown)—significant and strong effect; “signif” (light blue)—significant but weak effect; “strong” (green)—not significant but strong effect. **c** Rank order of anti-leukemia activity of the 2487 FDA-approved compounds in percentage of the positive control idarubicin. On the left the top drugs with the highest anti-leukemia activity doxorubicin, mitoxantrone, and daunorubicin are indicated in black. In red, the anthelmintic agents, ivermectin, moxidectin, and milbemycin are indicated. Funduscein, a drug with high fluorescent characteristics, leads to seemingly proliferative activity revealing a false negative example. **d** Validation of anthelmintic compounds, moxidectin, ivermectin, and milbemycin. Dose–response curves of the three drugs for B-R-03 are given normalized to vehicle treated control. *N* = 3 independent experiments. All quantifications represent mean ± s.e.m.

To identify novel compounds with anti-leukemia potential, we used a co-culture model of primary ALL cells on MSCs enabling long-term survival ex vivo (Fig. 1a)^15^. On this platform, we tested 2487 FDA-approved compounds (Supplementary Table S2) at 1 µM by using a fluorescent cell viability readout combined with automated imaging analysis. After normalization of cell viability to the effect of the highly potent anthracycline idarubicin, we identified 61 compounds with strong anti-leukemia activity (Fig. 1b, Supplementary Fig. S1A, B). As expected, the majority of these drugs were chemotherapeutic agents that are already in clinical use to treat leukemia. Among these, we found DNA-intercalating agents, including mitoxanthrone, daunorubicine, or doxorubicine, which appeared to have the strongest anti-leukemia effect among the screened compounds (Fig. 1c, Supplementary Fig. S1A). Furthermore, agents with known anti-leukemia activity, e.g. steroids, proteasome, and HDAC inhibitors, but also BCL-2 and cIAP antagonists or tyrosine kinase inhibitors also emerged with potent anti-leukemia activity (Supplementary Fig. S1A, B). Unexpectedly, we further identified several agents from drug families that have, so far, not been associated with anti-leukemia activity. For instance, anti-fungal and anti-bacterial agents as well as cardiac glycosides showed substantial anti-leukemia activity (Supplementary Fig. S1A, B). The most potent among these were the anthelmintic drugs ivermectin, moxidectin, and milbemycin, which showed up to 80% maximal cytotoxic effect compared to idarubicin (Fig. 1c).

### Anthelmintic agents are widely active in precursor B- and T-ALL

**Fig. 2:**
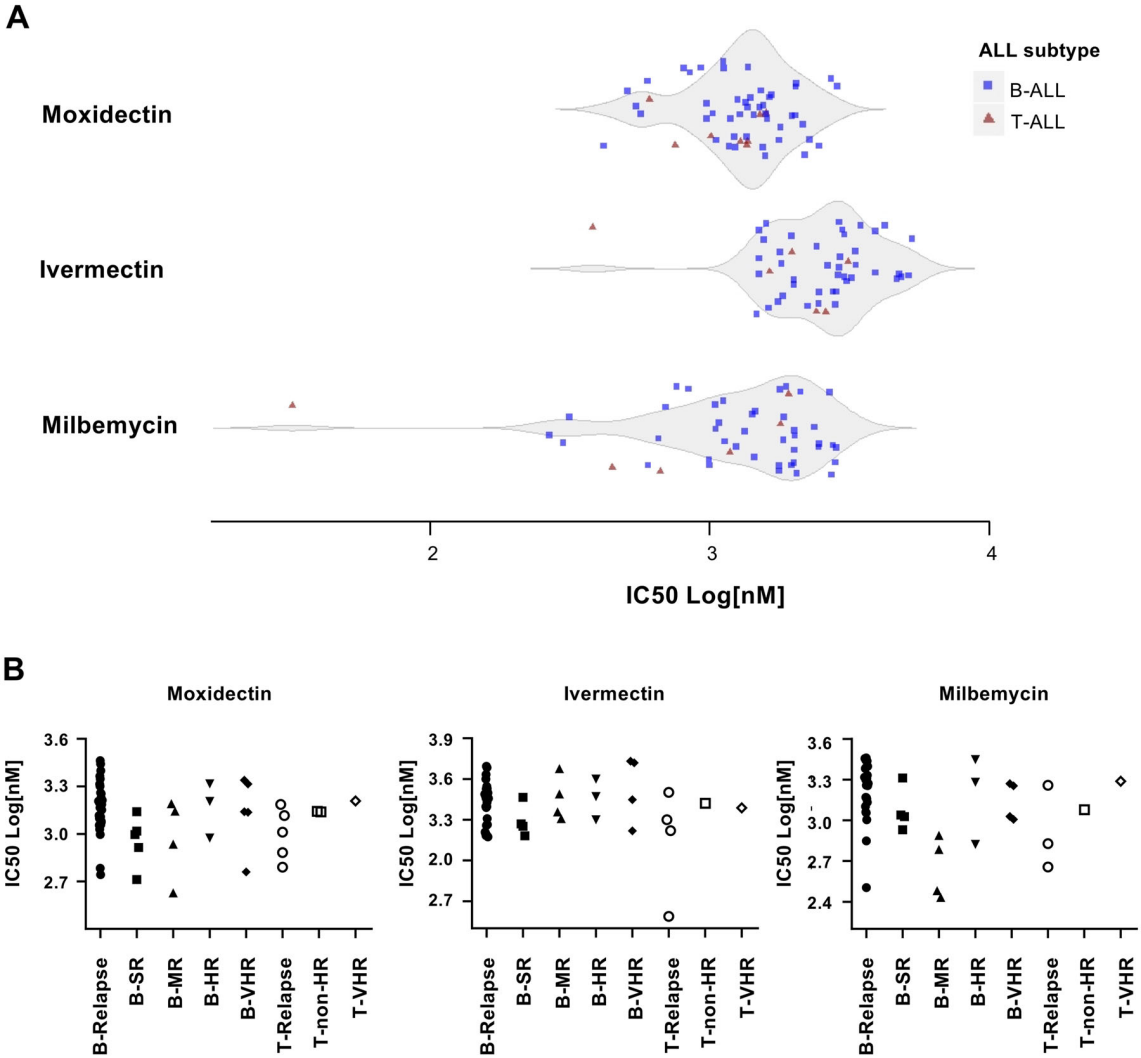
Primary B- and T-ALL samples are sensitive towards anthelmintic compounds. **a** Violin plot indicating the response of B-ALL (*n* = 47) and T-ALL (*n* = 8) samples to moxidectin, ivermectin, and milbemycin, as indicated by IC_50_ values. On the ordinate, the three drugs are indicated with the respective IC_50_ (Log nM) distribution. Blue squares represent precursor B-ALL and red triangles represent T-ALL samples. **b** Distribution of IC_50_ values in primary ALL with respect to their risk classification. IC_50_ values are given in Log (nM) of moxidetin, ivermectin, and milbemycin, and classification of primary ALL samples in standard risk (B-SR; *n* = 5), medium risk (B-MR; *n* = 4), high risk (B-HR; *n* = 3), very high risk (B-VHR; *n* = 5), and relapse (B-Relapse; *n* = 30). Classification of T-ALL samples in non-high risk (T-non-HR; *n* = 2), very high risk (T-VHR; *n* = 1), and relapse (T-Relapse; *n* = 5). Classification of samples at diagnosis in accordance to the criteria used in the ALL-BFM study^16^.

Having identified ivermectin, moxidectin, and milbemycin as potential candidates with anti-leukemia activity, we first validated the activity of these three drugs by generating dose response curves on the same ALL sample used for the screen. Interestingly, all three drugs showed a strong cytotoxic effect at low micromolar range concentration with IC_50_ values between 1.2 and 1.5 µM (Fig. 1d), with moxidectin showing a slightly higher activity as compared to ivermectin and milbemycin. To evaluate the potential of these anthelmintic compounds, we generated dose response curves for these three agents in 47 precursor B-ALL and 8 T-ALL PDXs (Fig. 2a). The tested T- and B-ALL samples responded to the three anthelmintic drugs with comparable sensitivities among the two leukemia types, with IC_50_ values between 300 nM and 2.5 µM for milbemycin and moxidectin, and a somewhat lower ivermectin sensitivity, with some of the tested cases showing IC_50_ values higher than 3 µM (Fig. 2a, Supplementary Table S3). Interestingly, most of the screened samples responded to moxidectin at IC_50_ values of 1–1.5 µM, while the responses to milbemycin were more spread among the samples (Fig. 2a, Supplementary Table S3). We next addressed whether we could identify differential sensitivities in samples from different risk groups, including SR, MR, HR, and VHR, according to the classification used in the ALL-BFM 2000 study as well as relapse samples^16^. With the exception of one relapsed T-ALL sample that showed very high sensitivity to ivermectin and milbemycin, we could not detect relevant differences in the sensitivity to all three anthelmintic agents across the samples. (Fig. 2b). It is worth noting though that most of the relapse samples responded to anthelmintic agents in the same sensitivity range as compared to the samples collected at diagnosis (Fig. 2b), suggesting that these agents target a mechanism that is not being selected during progression from diagnosis to relapse.

### Anthelmintic drugs induce cell death in absence of effector caspases and RIPK1

**Fig. 3:**
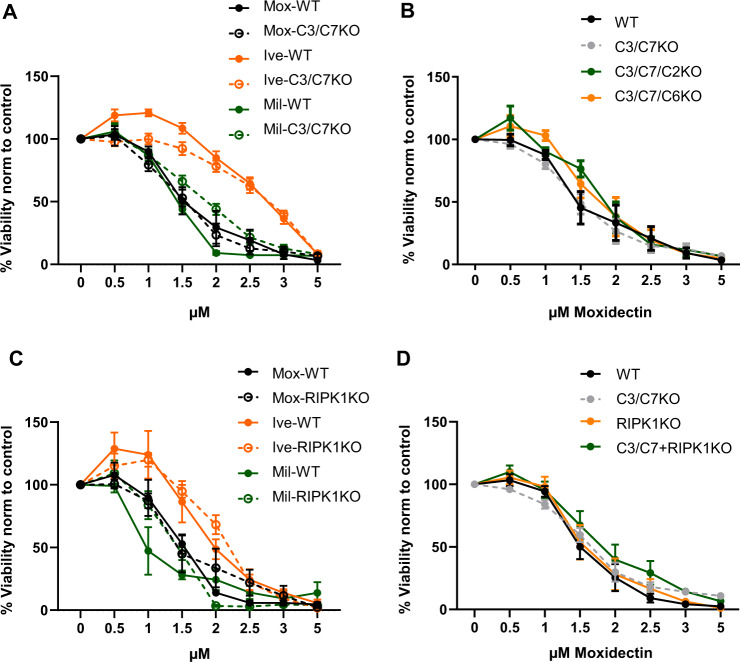
Anthelmintic agents induce cell death independent of caspases and RIPK1. Dose response curves of B-R-03 wild type (WT) and indicated gene knockout (KO) ALL are given. **a** Moxidectin (Mox; black), ivermectin (Ive; orange), and milbemycin (Mil; green) induce cell death in WT (continuous lines) and Caspases-3 and -7 KO (C3/C7KO; dotted lines) ALL. **b** Moxidectin induces cell death in WT (black), C3/C7KO (gray), Caspases-3/-7/-2 triple knockout (C3/C7/C2KO; green), and Caspases-3/-7/-6 triple knockout (C3/C7/C6KO; orange) ALL. **c** Moxidectin (black), ivermectin (orange), and milbemycin (green) induce cell death in WT (continuous lines) and RIPK1KO (dotted lines) ALL. **d** Moxidectin induces cell death in WT (black), C3/C7KO (gray), RIPK1KO (orange), and combination of C3/C7 and RIPK1 knockout (C3/C7+RIPK1KO; green). All the dose response curves were normalized to vehicle control, and performed in *N* = 3 independent experiments. Quantifications represent mean ± s.e.m.

Given the potential of anthelmintic agents to induce apoptotic cell death^26^ but also to activate other cell death pathways in other cellular systems^27,28^, we next addressed which cell death mechanism these drugs trigger in primary ALL. We tested moxidectin, ivermectin, and milbemycin in PDX samples that were depleted for the effector caspases-3 and -7 using CRISPR mediated gene editing (C3/C7KO; Supplementary Fig. 2A, E). Under apoptosis-incompetent conditions the cells were equally sensitive to moxidectin, the most potent anthelmintic agent in ALL (Fig. 2a), with no differences to wild-type (WT) cells (Fig. 3a, Supplementary Fig. S3A). Further suggesting a caspase-independent mechanism of cell death, triple knockout of Caspases-3/-7 and -6 (C3/C7/C2KO; Supplementary Fig. S2B) or Caspase-2 (C3/C7/C6KO; Supplementary Fig. S2C) did not rescue the cells upon moxidectin treatment (Fig. 3b). Given the potential of primary ALL cells to mount a necroptosis response upon depletion of SMAC mimetics using birinapant^11^, we also tested anthelmintic agents in ALL deficient for RIPK1 (RIPK1KO, Supplementary Fig. S2D), the key mediator of necroptosis. In several different primary RIPK1KO ALL samples, we could not detect any difference in the sensitivity to anthelmintic agents compared to WT cells (Fig. 3c, Supplementary Fig. S3B). Even upon simultaneous inactivation of both cell death pathways, either genetically upon knockout of caspases-3 and -7 and RIPK1 (C3/C7 + RIPK1KO), or upon pharmacological inhibitors, we could not detect any difference in the sensitivity (Fig. 3d, Supplementary Fig. S3C). We could also excluded the possibility that moxidectin was triggering autophagy (Supplementary Fig. S3D). Thus, anthelmintic agents eradicate ALL through an alternative, caspase-independent, non-necroptotic cell death mechanism.

### Moxidectin increases the intracellular chloride concentration and induces depolarization of the mitochondrial membrane in ALL cells

**Fig. 4:**
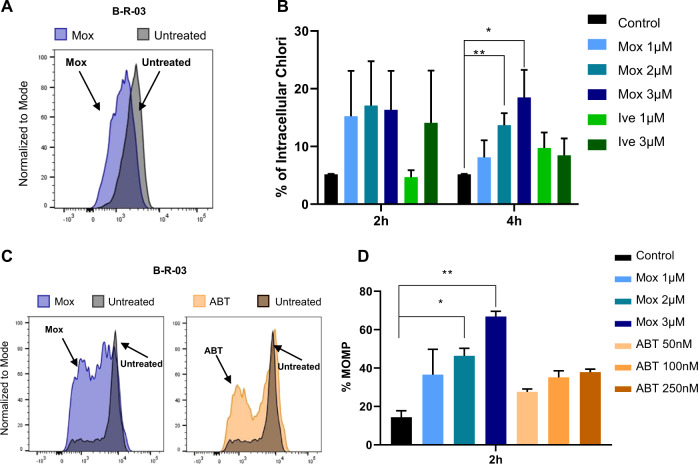
Moxidectin induces leukemia cell death by increasing intracellular chloride and inducing mitochondrial outer membrane permeabilization (MOMP). **a** Levels of intracellular chloride in B-R-03 PDX cells as measured by quenching of the MQAE fluorescent signal upon treatment with 3 µM moxidectin (Mox, blue), compared to vehicle control (black). Representation of one experiment after 4 h treatment. **b** Quantification of intracellular chloride in vehicle treated B-R-03 (black) compared to cells treated with moxidectin (Mox) 1 µM (light blue), 2 µM (blue), and 3 µM (dark blue) or ivermectin (Ive) 1 µM (light green) and 3 µM (green) for 2 or 4 h. Quantifications of *N* = 6 independent experiments representing mean ± s.e.m., Paired *t*-test, *p value ≤ 0.008; **p value ≤ 0.002. **c** MOMP levels in B-R-03 PDX cells. Light color histograms represent cells treated with 2 µM Moxidectin (Mox; blue) or 100 nM ABT-263 (ABT; orange) compared to control cells (black). Representation of one experiment after 2 h treatment. **d** MOMP quantification in control B-R-03 (black) ALL cells compared to cells treated with moxidectin (Mox) 1 µM (light blue), 2 µM (blue), and 3 µM (dark blue) or ABT-263 (ABT) 50 nM (light pink), 100 nM (orange), and 250 nM (brown) for 2 h. Quantifications of *N* = 3 independent experiments representing mean ± s.e.m. Paired t-test, *p value ≤ 0.02; **p value ≤ 0.01.

In nematodes, anthelmintic agents induce chloride influx by selectively binding GABA-A and glutamate-gated chloride channels increasing intracellular chloride concentrations^29–31^. In mammalian cells, which are lacking such glutamate-gated chloride channels, anthelmxintic agents may bind and modulate other ionotropic, ligand-gated transmembrane receptors including γ-aminobutyric acid type A (GABA_A_) receptors^32^, glycine receptors^33^, neuronal α_7_-nicotinic receptors^34^, and purinergic P2X(4) receptors^35^. Based on these data, we investigated the potential of anthelmintic agents to increase intracellular chloride in primary ALL. Indeed, moxidectin induced a rapid decrease of MQAE fluorescence indicative of increased intracellular chloride (Fig. 4a), already at 2 h of treatment. At 4 h of moxidectin treatment, this increase in intracellular chloride was even more dramatic (Fig. 4b, Supplementary Fig. S4A). Likewise, ivermectin treatment also increased intracellular chloride, albeit to a smaller extent than moxidectin. Further downstream, an increase in intracellular chloride may influence the mitochondria integrity as a gatekeeper for cell death and survival^36^. Notably, MOMP is considered a point of no return in cell death and is able to kill cells even in absence of caspase activity in a phenomenon known as MOMP-induced caspase-independent cell death^37^. Thus, we used TMRE to assess whether our anthelmintic agents destabilized the mitochondrial outer membrane potential. Indeed, moxidectin decreased the mitochondrial outer membrane potential of primary leukemia cells, indicating a strong destabilization of the mitochondria (Fig. 4c). Interestingly, the mitochondrial depolarization upon moxidectin treatment was even higher than upon treatment with the BCL-2-antagonist ABT-263, a potent inducer of MOMP^38^ (Fig. 4d, Supplementary Fig. S4B). Thus, our data suggest that moxidectin induces a strong mitochondrial permeabilization in primary leukemia cells that leads them to a caspase-independent cell death.

### Moxidectin synergizes with ABT-263 and dexamethasone

**Fig. 5:**
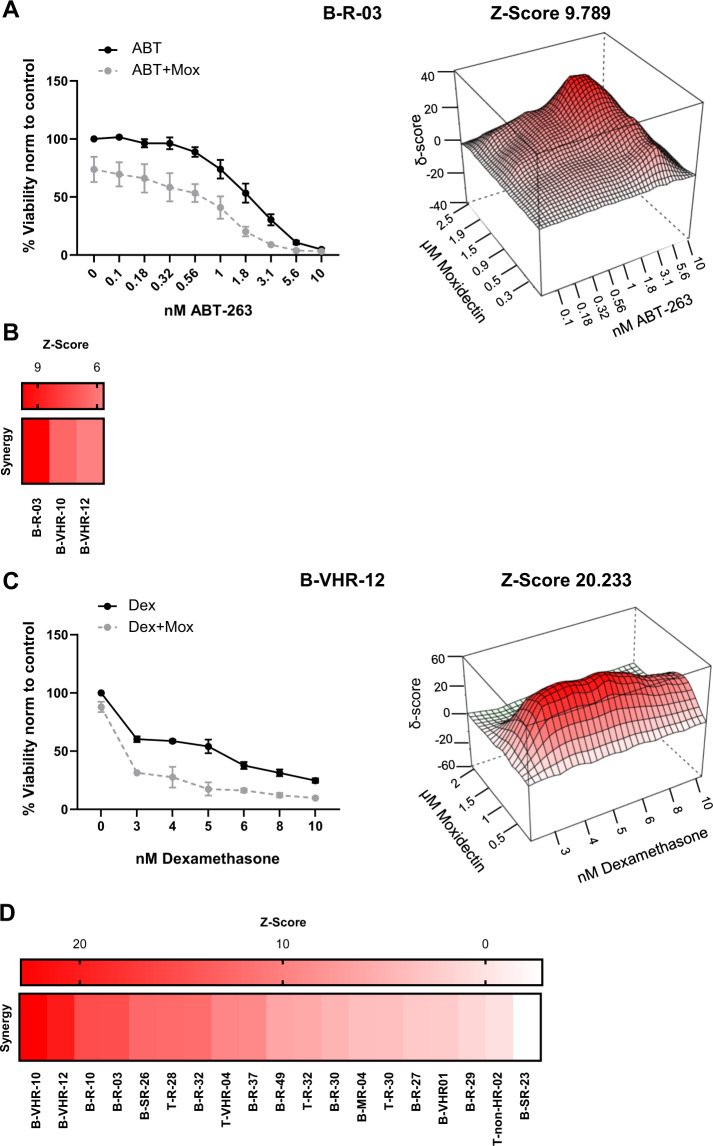
Synergic activity of moxidectin and ABT-263 or dexamethasone. **a** On the left, the viability curve of the B-R-03 sample treated with ABT-263 (ABT; black) or ABT-263 in combination with 0.9 µM moxidectin (ABT+Mox; gray). The dose response curve was performed in *N* = 3 independent experiments, and quantifications represent mean ± s.e.m. On the right, a 3D representation map of the B-R-03 sample with the calculated synergy (*Z*-score = 9.789) between moxidectin and ABT-263. **b** Heatmap representing the synergy between moxidectin and ABT-263 of B-ALL samples (*n* = 3). The samples (columns) were ordered, from the left to the right, according to their decreasing *Z*-score values. **c** On the left, the viability curve of the B-VHR-12 sample treated with dexamethasone (Dex; black) or dexamethasone in combination with 0.5 µM moxidectin (Dex+Mox; gray). The dose response curve was performed in *N* = 3 independent experiments, and quantifications represent mean ± s.e.m. On the right, a 3D representation map of the B-VHR-12 sample with the calculated synergy (*Z*-score = 20.233) between moxidectin and dexamethasone. **d** Heatmap representing the synergy between moxidectin and dexamethasone of B-ALL (*n* = 14) and T-ALL (*n* = 5) samples. The samples (columns) were ordered, from the left to the right, according to their decreasing *Z*-score values.

Given the diverging molecular mechanism of MOMP induction by ABT-263 and moxidectin, we evaluated the effects of the combination of moxidectin with ABT-263 using increasing concentrations of both agents simultaneously. In all the PDXs tested, sublethal concentrations of moxidectin enhanced the effect of ABT-263, resulting in synergistic drug activity with ZIP score values of 6.4, 5.6, and 9.7 (Fig. 5a, b, Supplementary Fig. S5A).

Further following the hypothesis that activation of cell death through different mechanisms may enhance killing in primary ALL, we investigated whether moxidectin may also sensitize to dexamethasone, a key component of standard induction chemotherapy in ALL^39^. Indeed, sublethal moxidectin increased dexamethasone-mediated leukemia cell death (Fig. 5c), and we observed a striking synergistic effect of moxidectin and dexamethasone in primary ALL samples, with a ZIP score ≥ 2.4 for 16 out of 19 primary ALL cases tested (Fig. 5d, Supplementary Fig. S5B, Supplementary Table S4). Thus, anthelmintic agents do not only possess attractive anti-leukemic potential as single agents, but also synergize with apoptosis inducers and standard chemotherapy to eradicate resistant leukemia cells.

## Discussion

Different approaches may be applied to identify new treatment strategies for resistant disease. While direct targeting of oncogenic lesions represents an appealing approach, its clinical translation has remained challenging frequently leading to only transient responses. By contrast, targeting specific pathways, on which leukemia cells rely, has been more promising for drug development, exemplified by venetoclax targeting BCL-2 or PI3K/AKT/mTOR inhibitors^7,8,10^. We here report on a drug repurposing screen that we conducted in refractory primary human ALL cells, using an extended FDA-approved drug library. We identified the anthelmintic agents, ivermectin, moxidectin, and milbemycin, as novel agents with high anti-leukemic potential for refractory ALL. These compounds had a strong anti-leukemic effect ex vivo in primary leukemia cells at low micromolar concentrations, extending earlier studies that described activity of ivermectin in acute myeloid leukemia (AML) and other primarily solid tumors^26,28,40,41^. Next to anthelmintic agents, we also identified other compound families with potential anti-leukemic agents, such as bactericidal and fungicidal agents. However, these compounds were active in concentrations that could never be reached in vivo, while the vast experience with anthelmintic agents indicates that these could be applied at active anti-leukemic concentrations in vivo (NCT03012828 (ref. ^42^)). The anthelmintic agent ivermectin has been widely used to eliminate ectoparasites^43^, while moxidectin has been recently approved by the FDA to treat onchocerciasis in humans^44^. Among the anthelmintic agents, moxidectin has earlier been shown to have a safer profile and a better efficiency when applied as antiparasitic treatment compared to ivermectin^44,45^. Furthermore, several clinical trials involving moxidectin did not report severe adverse effects at doses four time higher than the one used to treat human onchocerciasis (NCT03012828 (ref. ^46^)). Moreover, the chemical structure of moxidectin with high lipophilicity increases the half-life of the drug^47^, an observation that may be linked to its higher drug efficiency, as compared to other anthelmintic agents. Ivermectin on the other hand has been safely used in immunocompromised patients to treat scabies^48,49^. This attractive safety profile, together with the sensitizing activity towards steroids, warrants further clinical development of anthelmintics, possibly not as single agents but in combination with standard chemotherapy to eradicate minimal residual disease and to prevent relapse. Yet another aspect supporting clinical development arises from activity of anthelmintics across samples from different risk groups including SR ALL. The potential of these agents to improve the response to steroids also in good responding cases and potentially to decrease toxicity could be tested in a clinical trial.

In nematodes, anthelmintic agents target glutamate-gated chloride channels to increase intracellular chloride concentrations, which is lethal to the parasites^47^. Since these chloride channels are lacking in humans, anthelmintics must have alternative targets to activate cell death. While GABA-gated chloride channels have been shown to act as potential mediators to increase intracellular chloride upon ivermectin^50^, targeting the Ca^2+^-activated chloride channel TMEM16A by anthelmintics was recently shown to confer susceptibility to these agents^51^. TMEM16A is indeed widely expressed in human cancers^52^, but also in human leukemia (https://pecan.stjude.cloud/home), and an increase in intracellular chloride upon expression of synthetic chloride transporters has been reported to induce mitochondrial alterations and cell death^36,53^. Further supporting a role for TMEM16A in tumor biology, its expression also increased BCL-2 with subsequent breast tumor progression^54^, and its downregulation induced apoptosis^55^. Future functional experiments including for instance CRISPR-based loss of function studies will shed light on the mechanistic requirement of chloride channels for moxidectin action, taking into consideration that several different mechanisms may contribute to chloride homeostasis in ALL. Irrespective of the underlying control mechanism, intracellular chloride is increased upon treatment with moxidectin, which leads to depolarization of the mitochondrial outer membrane and cell death, corroborating the results of a previous study in AML^40^. The balance of intracellular chloride is required for cellular homeostasis^56^, and alteration of its intracellular concentrations can destabilize mitochondria and trigger cell death. Susceptibility to increased intracellular chloride thus appears to represent a distinct vulnerability in leukemia cells, which does neither depend on presence of the apoptosis executioner caspases-3 and -7 nor on activation of the alternate necroptosis cell death mechanism. Rather, osmotic stress may underlie the potent anti-leukemia activity of anthelmintic agents. Underscoring the importance of mitochondrial integrity for survival and cell death in leukemia, synergistic effect of increasing intracellular chloride using moxidectin and antagonizing BCL-2 using navitoclax enhanced the cell death mechanism. Such dual induction of mitochondrial damage may also underlie the synergistic activity of moxidectin with dexamethasone, which has been shown to upregulate BCL-2 family members to reactivate apoptosis^57^. Thus, we envisage that single agent activity of anthelmintics will not be a solution to treat resistant disease. Rather, combination with standard chemotherapy should be tested in a clinical trial in order to assess their anti-leukemic potential.

Taken together, our data show that repurposing screens do identify novel agents with anti-cancer activity. While such phenotypic screens, as opposed to CRISPR-based genomic screens, may not identify molecular mechanisms that confer sensitivity or resistance to given approaches, they do provide the basis for the identification of new agents with so far unappreciated anti-cancer activity. Based on the knowledge obtained on the agents so far, it will be possible to develop and validate biomarkers to identify vulnerabilities and potential responses. The ability of anthelmintics to induce cell death independent on apoptosis and necroptosis underscores their potential as novel anti-leukemic agents, supporting the notion that simultaneous activation of several different cell death pathways represents a powerful approach to develop novel treatment strategies for refractory disease.

## Supplementary information

checklist

supplementary table 1

supplementary table 2

supplementary table 3

supplementary table 4

supplemental figures
