# Supplementary material for: Repurposing anthelmintic agents to eradicate resistant leukemia
Source: Blood Cancer J. 2020 Jun 26;10(6):72. doi: 10.1038/s41408-020-0339-9 (PMC7320149; doi:10.1038/s41408-020-0339-9)
Supplement: Supplementary file 2 — supplementary table 1 [file 41408_2020_339_MOESM2_ESM.pdf]

Supplementary Table S1: Antibodies, chemicals, and reagents

REAGENT or RESOURCE	SOURCE	IDENTIFIER
**Antibodies**		
rabbit anti-Caspase 3	Cell Signaling	9662
rabbit anti-Caspase 7	Cell Signaling	9492
mouse anti-Caspase 2	Cell Signaling	2224
rabbit anti-Caspase 6	Cell Signaling	9762
mouse anti-Tubulin	Sigma Aldrich	T9026
mouse anti-RIPK1	BD Pharmingen	51-6559GR
hCD19-PE-Cy7	Biolegend	302208
hCD45-Alexa Fluor 647	Biolegend	304018
anti-mouse IgG HRP	Cell Signaling	7076
anti-rabbit IgG HRP	Cell Signaling	7074
**Chemicals and reagents**		
Drug library	NEXUS platform ETH	
Z-VAD-FMK	Selleckchem	S7023
Necrostatin-1s	Bio Vision	2263-1
Chloroquine	Sigma Aldrich	C1650000
Bafilomycin	Selleckchem	S1413
Moxidectin	Sigma Aldrich	33746
Ivermectin	Selleckchem	S1351
Milbemycin	Sigma Aldrich	Y0001882
ABT-263	Selleckchem	S1001
Dexamethasone	Selleckchem	S1322
TMRE	Thermo Fisher Scientific	T669
MQAE	Thermo Fisher Scientific	E3101
CyQUANT	Thermo Fisher Scientific	C35012
AIM-V	Thermo Fisher Scientific	12055083
