# Supplementary material for: Repurposing anthelmintic agents to eradicate resistant leukemia
Source: Blood Cancer J. 2020 Jun 26;10(6):72. doi: 10.1038/s41408-020-0339-9 (PMC7320149; doi:10.1038/s41408-020-0339-9)
Supplement: Supplementary file 3 — supplementary table 2 [file 41408_2020_339_MOESM3_ESM.pdf]

Supplementary Table S2: Statistical analysis and drug list of the 2487 FDA-approved compounds screened

Raw	Norm	Cor.loess	% Cor.loess	Z	Pval	Padj	Drug.name
5.759668	1.205661	1.451110951	145.1110951	27.44886	0	5.60E-15	DOXORUBICIN
5.78746	1.159104	1.43015272	143.015272	112.0519	0	5.60E-15	BENZETHONIUM CHLORIDE
5.777427	1.124441	1.35592802	135.592802	37.1658	0	5.60E-15	MITOXANTRONE HYDROCHLORIDE
5.819544	1.090739	1.330715993	133.0715993	104.2611	0	5.60E-15	DAUNORUBICIN HYDROCHLORIDE
5.893762	0.938997	1.25268621	125.268621	21.07088	0	5.60E-15	Idarubicin·HCl
5.835056	1.053002	1.246864123	124.6864123	23.58538	0	5.60E-15	DACTINOMYCIN
5.960946	0.788341	1.019427228	101.9427228	39.17386	0	5.60E-15	OUABAIN OCTAHYDRATE
5.97635	0.756611	1.017156375	101.7156375	79.69378	0	5.60E-15	COLCHICINE
5.937016	0.832605	1.015791721	101.5791721	22.17391	0	5.60E-15	Dinaciclib (SCH727965)
5.972203	0.765447	1.014244323	101.4244323	79.46563	0	5.60E-15	DIGOXIN
6.017033	0.674257	0.992634967	99.26349667	21.99658	0	5.60E-15	MLN9708
5.960946	0.78505	0.982834237	98.28342375	21.45447	0	5.60E-15	Flavopiridol (Alvocidib)
5.992111	0.723117	0.961519902	96.15199018	20.9892	0	5.60E-15	Carfilzomib (PR-171)
5.928908	0.863891	0.960692702	96.06927022	21.28875	0	5.60E-15	Romidepsin (FK228, Depsipeptide)
5.992554	0.703559	0.924315502	92.43155018	25.33536	0	5.60E-15	SELAMECTIN
6.089905	0.517448	0.895854365	89.58543653	19.85194	0	5.60E-15	Octenidine
6.029384	0.659496	0.860263327	86.02633273	16.27253	0	5.60E-15	VINCRISTINE SULFATE
5.966142	0.774725	0.845579913	84.55799127	18.45832	0	5.60E-15	Bortezomib (PS-341)
6.071882	0.563695	0.839635375	83.96353749	32.26494	0	5.60E-15	CETRIMONIUM BROMIDE
6.093422	0.50962	0.82872777	82.87277699	13.93966	0	5.60E-15	Fosbretabulin (Combretastatin A4 Phosphate (CA4P)) Disodium
6.060698	0.596086	0.815174089	81.51740895	15.41963	0	5.60E-15	IVERMECTIN
6.071882	0.564592	0.80746282	80.74628203	17.62626	0	5.60E-15	CEP-18770 (Delanzomib)
5.977266	0.752618	0.803035687	80.30356866	17.52962	0	5.60E-15	MLN2238
6.064458	0.588472	0.801376916	80.13769162	15.15865	0	5.60E-15	PODOFILOX
6.089905	0.514643	0.781335047	78.13350465	61.21728	0	5.60E-15	CETYLPYRIDINIUM CHLORIDE
6.093422	0.532859	0.765995725	76.59957245	16.33613	0	5.60E-15	PUROMYCIN DIHYDROCHLORIDE
6.045323	0.630458	0.732303199	73.23031993	15.61758	0	5.60E-15	VINBLASTINE SULFATE
6.100371	0.507978	0.723091846	72.30918458	15.78451	0	5.60E-15	Bardoxolone Methyl
6.049218	0.609631	0.718040585	71.8040585	15.67424	0	5.60E-15	Vinorelbine Tartrate
6.11059	0.48767	0.704435756	70.44357559	15.37726	0	5.60E-15	Quisinostat (JNJ-26481585)
6.133539	0.421666	0.669922183	66.99221827	52.48813	0	5.60E-15	GENTIAN VIOLET
6.133539	0.423554	0.651510268	65.15102679	14.43733	0	5.60E-15	Homoharringtonine
6.0086	0.690349	0.643561298	64.35612977	14.04842	0	5.60E-15	ABT-263 (Navitoclax)
6.155336	0.407227	0.642100705	64.21007049	13.69386	0	5.60E-15	THIMEROSAL
6.155336	0.37522	0.629812773	62.98127734	49.34557	0	5.60E-15	DIGITOXIN
6.190332	0.329202	0.583973851	58.39738514	12.74767	0	5.60E-15	ABT-199 (GDC-0199)
6.190332	0.333583	0.536040796	53.60407958	10.13962	0	5.60E-15	MOXIDECTIN
6.255273	0.161601	0.527174052	52.71740516	11.68207	0	5.60E-15	Pixantrone dimaleate
6.181844	0.318736	0.526737437	52.67374374	41.26966	0	5.60E-15	GRAMICIDIN (gramicidin A shown)
6.217484	0.28112	0.515946929	51.59469292	11.00342	0	5.60E-15	LANATOSIDE C
6.220108	0.237201	0.506032072	50.60320717	39.64741	0	5.60E-15	EMETINE DIHYDROCHLORIDE
6.220108	0.258362	0.494729901	49.47299007	13.56048	0	5.60E-15	AMSACRINE
6.190332	0.316618	0.494051791	49.40517907	13.54189	0	5.60E-15	CHLOROXINE
6.149219	0.389813	0.48484023	48.48402296	10.74396	0	5.60E-15	10-Hydroxycamptothecin
6.267172	0.135964	0.478914123	47.89141235	8.055601	8.88E-16	2.22E-14	CHLOROTHALONIL
6.209515	0.29729	0.477479943	47.74799432	10.18305	0	5.60E-15	PHENYLMERCURIC ACETATE
6.232996	0.244417	0.474998632	47.49986321	10.36883	0	5.60E-15	YM155 (Sepantronium Bromide)
6.260071	0.151233	0.462760435	46.27604354	7.783887	7.11E-15	1.68E-13	Domiphen Bromide
6.240549	0.193644	0.453255041	45.32550414	35.51235	0	5.60E-15	PYRITHIONE ZINC
6.243038	0.213501	0.43867357	43.86735701	12.02398	0	5.60E-15	METHYLBENZETHONIUM CHLORIDE
6.227887	0.243144	0.436934812	43.69348124	11.97632	0	5.60E-15	PENFLURIDOL
6.220108	0.270028	0.425281352	42.5281352	9.283546	0	5.60E-15	Panobinostat (LBH589)
6.262451	0.187544	0.397260322	39.7260322	7.514479	5.71E-14	1.25E-12	DI-O-DEMETHYLCURCUMIN
6.281033	0.148954	0.379073969	37.90739691	8.274876	2.22E-16	5.60E-15	Birinapant
6.292256	0.126652	0.365755471	36.57554707	7.984144	1.33E-15	3.26E-14	TG101348 (SAR302503)
6.285557	0.139964	0.344655084	34.46550838	7.52354	5.33E-14	1.18E-12	Pelitinib (EKB-569)
6.294466	0.112885	0.341617201	34.16172009	9.36368	0	5.60E-15	DRONEDARONE HYDROCHLORIDE
6.290035	0.131066	0.340285898	34.02858976	7.428164	1.10E-13	2.40E-12	Dacomitinib (PF299804, PF299)
6.322219	0.056759	0.333826341	33.38263414	12.82805	0	5.60E-15	TYLOXAPOL
6.238046	0.19867	0.33062738	33.06273801	7.326633	2.36E-13	4.93E-12	Milbemycin
6.322219	0.056759	0.311805903	31.18059027	11.98187	0	5.60E-15	OXALIPLATIN
6.338456	0.023878	0.292442098	29.24420981	11.23777	0	5.60E-15	TOLONIUM CHLORIDE
6.352183	-0.00392	0.289602735	28.96027347	11.12866	0	5.60E-15	ANCITABINE HYDROCHLORIDE
6.227887	0.25457	0.28140822	28.14082203	6.142913	8.10E-10	1.28E-08	Napabucasin
6.340444	0.03089	0.259198563	25.9198563	5.658094	1.53E-08	2.08E-07	AT13387
6.252853	0.20698	0.257881287	25.78812866	4.87802	1.07E-06	1.16E-05	TRAMADOL HYDROCHLORIDE
6.348305	0.015268	0.254767996	25.47679956	5.561378	2.68E-08	3.49E-07	Ganetespib (STA-9090)
6.318063	0.065174	0.254342317	25.43423165	9.773695	0	5.60E-15	SALINOMYCIN, SODIUM
6.257679	0.195366	0.245840378	24.58403782	5.366496	8.03E-08	9.93E-07	Nintedanib (BIBF 1120)
6.305351	0.091589	0.23186097	23.18609701	6.355277	2.08E-10	3.44E-09	BENZALKONIUM CHLORIDE HYDRATE
6.383815	-0.11488	0.230700566	23.07005659	3.880512	0.000104	0.000787	Misoprostol
6.334454	0.043773	0.230281609	23.02816087	4.911137	9.05E-07	9.93E-06	PYRVINIUM PAMOATE
6.361728	-0.01141	0.225194594	22.51945944	4.915815	8.84E-07	9.71E-06	GSK2126458 (GSK458)
6.399674	-0.14899	0.223770529	22.37705294	3.763944	0.000167	0.00122	GEMCITABINE HYDROCHLORIDE
6.294466	0.077266	0.221962789	22.19627885	3.733537	0.000189	0.001369	THYMOPENTIN
6.359835	-0.00966	0.218982718	21.89827175	4.142224	3.44E-05	0.00029	EPIRUBICIN HYDROCHLORIDE
6.357935	-0.01557	0.21237237	21.23723699	8.160902	2.22E-16	5.60E-15	HOMIDIUM BROMIDE
6.354108	-0.00782	0.199775383	19.97753828	7.676833	1.62E-14	3.70E-13	ETHACRIDINE LACTATE
6.365488	-0.07258	0.195694479	19.56944787	15.33258	0	5.60E-15	CYTARABINE
6.334454	0.042794	0.195120631	19.51206307	4.259325	2.05E-05	0.000182	Volasertib (BI 6727)
6.354108	0.003735	0.19369457	19.36945697	4.228195	2.36E-05	0.000207	GDC-0941
6.340444	-0.02161	0.193284852	19.3284852	3.251158	0.001149	0.007031	PIRFENIDONE
6.38739	-0.06891	0.192164774	19.21647737	5.26721	1.39E-07	1.67E-06	TOPOTECAN HYDROCHLORIDE
6.307496	0.096366	0.189434423	18.94344228	4.135199	3.55E-05	0.000298	Rigosertib (ON-01910)
6.342423	0.025605	0.18918344	18.91834399	3.578548	0.000346	0.002362	PARAMETHADIONE
6.399674	-0.09033	0.186264266	18.62642661	3.523329	0.000426	0.002863	ACRIFLAVINIUM HYDROCHLORIDE
6.385606	-0.0716	0.18288642	18.28864195	7.027836	2.10E-12	4.02E-11	TRICHLORMETHINE HYDROCHLORIDE
6.348305	-0.03597	0.18027819	18.02781903	14.12472	0	5.60E-15	AMODIAQUINE DIHYDROCHLORIDE
6.357935	-0.05649	0.179785362	17.9785362	14.08611	0	5.60E-15	MITOMYCIN
6.390935	-0.07585	0.177481692	17.74816919	4.864748	1.15E-06	1.24E-05	HYDROQUINONE
6.365488	-0.03086	0.176496109	17.64961095	6.782273	1.18E-11	2.11E-10	URAPIDIL HYDROCHLORIDE
6.40824	-0.10384	0.176374332	17.63743315	3.850108	0.000118	0.000886	Neratinib (HKI-272)
6.4133	-0.11792	0.176345754	17.63457536	3.335713	0.000851	0.00538	EVANS BLUE
6.385606	-0.05886	0.17031107	17.03110698	3.717752	0.000201	0.001452	Sotagliflozin (LX4211)
6.385606	-0.05886	0.168288745	16.82887452	3.673606	0.000239	0.001696	Crenolanib (CP-868596)
6.352183	0.007562	0.165710461	16.57104606	3.617325	0.000298	0.002074	Dovitinib (TKI-258, CHIR-258)
6.409933	-0.1111	0.16385167	16.38516697	3.099378	0.001939	0.011045	PHTHALYLSULFACETAMIDE
6.392697	-0.0762	0.163409719	16.34097188	3.091018	0.001995	0.011323	METHYLENE BLUE
6.374748	-0.04418	0.16232216	16.23221601	4.449228	8.62E-06	8.10E-05	THIRAM
6.437751	-0.17719	0.161031961	16.10319605	6.188027	6.09E-10	9.81E-09	TIRATRICOL
6.382017	-0.05173	0.15980301	15.98030098	3.48837	0.000486	0.003215	PCI-24781 (Abexinostat)
6.369216	-0.03336	0.159763348	15.97633479	4.379091	1.19E-05	0.00011	PERHEXILINE MALEATE
6.376577	-0.04092	0.159421248	15.94212482	3.480036	0.000501	0.003295	Cladribine
6.374748	-0.09549	0.15675418	15.67541797	3.473639	0.000513	0.003364	Revaprazan hydrochloride
6.385606	-0.06543	0.154341138	15.43411383	4.230469	2.33E-05	0.000205	ACEDAPSONE
6.421604	-0.19615	0.153635071	15.36350707	2.584227	0.00976	0.042276	Salmeterol
6.371068	-0.08447	0.150904404	15.09044038	11.8233	0	5.60E-15	BITHIONOL
6.32838	0.054041	0.149603336	14.96033361	2.82986	0.004657	0.023042	BENZOXIQUINE
6.437751	-0.17719	0.148406311	14.84063112	5.702857	1.18E-08	1.62E-07	DIBUTYL PHTHALATE
6.32838	0.054865	0.148342299	14.83422994	3.238192	0.001203	0.007306	LY2784544
6.369216	-0.03841	0.146796462	14.6796462	5.640995	1.69E-08	2.27E-07	HEXESTROL
6.367356	-0.02488	0.146024627	14.60246268	2.762166	0.005742	0.027348	FLUDARABINE PHOSPHATE
6.389166	-0.06725	0.145641453	14.56414534	3.106046	0.001896	0.010814	MENADIONE
6.372912	-0.03426	0.143788626	14.37886257	3.066531	0.002166	0.012078	SULFAMERAZINE
6.390935	-0.07084	0.14137143	14.13714303	3.01498	0.00257	0.013983	BENZYL BENZOATE
6.382017	-0.11113	0.140624696	14.06246963	3.116213	0.001832	0.010527	Carvedilol EP IMpurity E
6.356026	-0.05512	0.139981697	13.99816973	2.35457	0.018544	0.070203	OLOPATADINE HYDROCHLORIDE
6.40824	-0.10384	0.139936751	13.99367512	3.054705	0.002253	0.012456	Pracinostat (SB939)
6.404834	-0.09904	0.138363089	13.83630888	2.950822	0.003169	0.016694	LEVONORGESTREL
6.385606	-0.06543	0.137761032	13.77610323	3.776011	0.000159	0.001171	METARAMINOL BITARTRATE
6.392697	-0.07441	0.137006026	13.70060256	2.921881	0.003479	0.018067	VANILLIN
6.380211	-0.05092	0.136517215	13.6517215	2.582326	0.009814	0.042473	OLANZAPINE
6.357935	-0.01557	0.136209396	13.62093962	5.234163	1.66E-07	1.99E-06	RIBOSTAMYCIN SULFATE
6.378398	-0.10009	0.135532563	13.55325635	10.61892	0	5.60E-15	CHLORAMPHENICOL SODIUM SUCCINATE
6.356026	-0.05512	0.134339858	13.43398584	2.259671	0.023842	0.084791	VOGLIBOSE
6.352183	-0.04686	0.133764154	13.3764154	2.249987	0.02445	0.086512	ABAMECTIN (avermectin B1a shown)
6.389166	-0.06725	0.133635717	13.36357175	2.850003	0.004372	0.021967	TAMOXIFEN CITRATE

Raw: raw signal
Norm: normlized signal
Cor.loess: normalized and geometry-corrected signal
% Cor.loess: cor.loss in percentage to positive control
Z: z-score
Pval: two-sided raw p-value
Padj: two-sided adjusted p-value (=FDR)

6.389166	-0.06725	0.133129332	13.31293316	2.839204	0.004523	0.022547	TRIPELENNAMINE CITRATE
6.378398	-0.04724	0.133031954	13.30319539	2.5164	0.011856	0.049513	FESOTERODINE FUMARATE
6.431364	-0.21714	0.132757069	13.27570694	2.233048	0.025546	0.089524	Voxtalisib (XL765, SAR245409)
6.432969	-0.15775	0.132614576	13.26145757	2.508505	0.012124	0.050346	NALBUPHINE HYDROCHLORIDE
6.40654	-0.16006	0.132394244	13.23942438	10.37303	0	5.60E-15	PROMAZINE HYDROCHLORIDE
6.356026	-0.00194	0.13230206	13.23020604	2.502594	0.012329	0.051112	CARAZOLOL
6.41162	-0.11632	0.132087457	13.20874568	3.620499	0.000294	0.002054	CILOSTAZOL
6.361728	-0.01349	0.132009849	13.20098489	2.497066	0.012523	0.051748	ANTIMONY POTASSIUM TARTRATE TRIHYDRATE
6.369216	-0.08052	0.129484521	12.94845212	10.14506	0	5.60E-15	SANGUINARIUM CHLORIDE
6.401401	-0.09207	0.129337528	12.93375282	2.758337	0.00581	0.027621	FLUNARIZINE HYDROCHLORIDE
6.396199	-0.08152	0.129336419	12.93364185	2.758314	0.00581	0.027621	IODOFORM
6.352183	-0.04423	0.129114271	12.91142711	10.11605	0	5.60E-15	CHLORTETRACYCLINE HYDROCHLORIDE
6.4133	-0.11622	0.129087724	12.90877239	2.75301	0.005905	0.02793	IDOXURIDINE
6.424882	-0.13691	0.128524115	12.85241152	2.805577	0.005023	0.024468	S-Ruxolitinib (INCB018424)
6.41162	-0.11281	0.128017683	12.80176833	2.73019	0.00633	0.029624	ECONAZOLE NITRATE
6.432969	-0.16751	0.127967743	12.79677431	4.917458	8.77E-07	9.65E-06	CAMYLOFINE DIHYDROCHLORIDE
6.40824	-0.16741	0.127141193	12.71411929	2.138585	0.032469	0.10788	ADEFOVIR DIPIVOXYL
6.378398	-0.10323	0.126604023	12.66040231	2.12955	0.033209	0.109767	TAZOBACTAM
6.399674	-0.08857	0.126363	12.63630003	2.694901	0.007041	0.032303	CYCLOSPORINE
6.399674	-0.08857	0.126290357	12.62903567	2.693351	0.007074	0.032407	CINTRIAMIDE
6.385606	-0.06184	0.126282085	12.62820847	2.388721	0.016907	0.065679	UNDECYLENIC ACID
6.429752	-0.21367	0.125197388	12.51973881	2.10589	0.035214	0.114838	Sunitinib
6.424882	-0.13691	0.123103489	12.3103489	2.687249	0.007204	0.032907	Defactinib (VS-6063, PF-04554878)
6.431364	-0.21732	0.122761903	12.27619025	2.720378	0.006521	0.030296	Irinotecan·HCl
6.403121	-0.10707	0.121733183	12.17331833	4.677881	2.90E-06	2.92E-05	MEPARFYLON
6.447158	-0.2511	0.121707624	12.17076245	2.04719	0.040639	0.129319	Dacarbazine
6.40654	-0.1025	0.121512638	12.15126376	2.591459	0.009557	0.041538	DIPERODON HYDROCHLORIDE
6.4133	-0.17829	0.121319724	12.13197239	2.040665	0.041284	0.13072	PHENYLALANINE (L) HYDROCHLORIDE
6.396199	-0.08615	0.121164657	12.1164657	3.321106	0.000897	0.005628	TENIPOSIDE
6.390935	-0.13032	0.120911044	12.09110437	2.679363	0.007376	0.03357	Abiraterone Acetate
6.444045	-0.24441	0.120424856	12.04248557	2.025613	0.042804	0.134536	Palbociclib (PD-0332991) HCl
6.414973	-0.18205	0.120289782	12.02897819	2.665596	0.007685	0.034656	Efonidipine(NZ-105)
6.409933	-0.10939	0.119446536	11.94465363	2.547396	0.010853	0.046384	AMINACRINE
6.38739	-0.06364	0.118377536	11.83775357	2.524597	0.011583	0.04867	YOHIMBINE HYDROCHLORIDE
6.348305	-0.03597	0.117668046	11.76680462	9.219243	0	5.60E-15	GRISEOFULVIN
6.403121	-0.09969	0.117204952	11.72049517	3.212571	0.001316	0.007882	OCTINOXATE
6.453318	-0.26456	0.116992795	11.69927952	2.592536	0.009527	0.041488	Mexiletine·HCl
6.416641	-0.123	0.115962125	11.59621253	2.473085	0.013395	0.054475	EPRINOMECTIN
6.457882	-0.21796	0.11593197	11.59319696	4.454955	8.39E-06	7.91E-05	ARTENIMOL
6.403121	-0.09969	0.115928426	11.59284262	3.177582	0.001485	0.00877	OXIBENDAZOLE
6.454845	-0.26763	0.115347138	11.53471384	1.940203	0.052355	0.155306	Miglustat (N-Butyldeoxynojirimycin·HCl)
6.403121	-0.09556	0.115009108	11.50091084	2.45276	0.014176	0.057217	PROPARACAINE HYDROCHLORIDE
6.394452	-0.13789	0.114023076	11.40230757	2.526727	0.011513	0.048436	Disopyramide
6.389166	-0.07881	0.113642141	11.36421411	4.366963	1.26E-05	0.000115	CINCHONIDINE
6.456366	-0.27091	0.113106276	11.31062763	1.90251	0.057104	0.164811	Citalopram·HBr
6.41162	-0.17484	0.11233449	11.23344899	2.489308	0.012799	0.052678	Etofylline
6.418301	-0.12637	0.111770197	11.17701971	2.383685	0.01714	0.066283	ETOPOSIDE
6.376577	-0.04092	0.111213469	11.12134694	2.4277	0.015195	0.060309	Dasatinib
6.414973	-0.17803	0.110916062	11.0916062	8.690228	0	5.60E-15	METHOTREXATE HYDRATE
6.378398	-0.05132	0.110778184	11.07781841	3.036415	0.002394	0.013138	MONOBENZONE
6.344392	0.023044	0.110225135	11.02251347	2.406125	0.016123	0.063401	Selumetinib (AZD6244)
6.404834	-0.15642	0.110135064	11.01350642	8.629037	0	5.60E-15	DYCLONINE HYDROCHLORIDE
6.385606	-0.11886	0.110062181	11.00621811	2.438955	0.01473	0.058919	Sulfalen
6.416641	-0.12614	0.109571722	10.95717216	3.003346	0.00267	0.014437	PRAMIPEXOLE DIHYDROCHLORIDE
6.426511	-0.14545	0.109374653	10.9374653	2.997944	0.002718	0.014634	APRAMYCIN SULFATE
6.380211	-0.04814	0.109164222	10.91642221	2.382966	0.017174	0.066312	KPT-330
6.38739	-0.11925	0.109082408	10.90824081	8.546562	0	5.60E-15	BACITRACIN
6.389166	-0.07881	0.10893608	10.893608	4.186122	2.84E-05	0.000243	CINOCTRAMIDE
6.41162	-0.11281	0.108867815	10.88678149	2.321787	0.020244	0.075254	LOFEXIDINE HYDROCHLORIDE
6.40824	-0.10971	0.108474843	10.84748435	2.97328	0.002946	0.015649	ANETHOLE
6.416641	-0.12614	0.108225802	10.82258023	2.966454	0.003013	0.015967	PIMOBENDAN
6.409933	-0.17121	0.107936774	10.7936774	2.391856	0.016763	0.06532	Ubenimex
6.403121	-0.15277	0.107906914	10.79069138	8.454462	0	5.60E-15	MECHLORETHAMINE
6.40824	-0.10595	0.107488576	10.74885755	2.292372	0.021884	0.079806	CANRENOIC ACID, POTASSIUM SALT
6.404834	-0.10304	0.107376382	10.73763817	2.943172	0.003249	0.017077	SUMATRIPTAN
6.385606	-0.0716	0.107187716	10.71877156	4.118937	3.81E-05	0.000318	SULFAPHENAZOLE
6.429752	-0.14659	0.107053413	10.70534131	2.336889	0.019445	0.072883	CUDC-101
6.424882	-0.13691	0.106353432	10.63534319	2.321609	0.020254	0.075254	Molidustat (BAY 85-3934)
6.414973	-0.11961	0.105922479	10.59224789	2.258973	0.023885	0.084807	NIFURSOL
6.394452	-0.1343	0.105493413	10.54934132	8.265366	2.22E-16	5.60E-15	OXYBENZONE
6.419956	-0.12712	0.105261716	10.5261716	2.297778	0.021574	0.078901	Ponatinib (AP24534)
6.450249	-0.2025	0.105258396	10.52583959	4.044799	5.24E-05	0.000423	NIFUROXAZIDE
6.409933	-0.12086	0.105153261	10.51532607	4.040758	5.33E-05	0.000429	FAMPROFAZONE
6.40824	-0.16368	0.105053518	10.50535183	8.2309	2.22E-16	5.60E-15	PROCAINE HYDROCHLORIDE
6.444045	-0.24461	0.10467894	10.46789398	2.319663	0.020359	0.075532	Metaproterenol Hemisulfate (Orciprenaline)
6.4133	-0.11389	0.104642645	10.46426445	2.284264	0.022356	0.081237	OSI-906 (Linsitinib)
6.403121	-0.09556	0.103840795	10.38407949	2.214577	0.026789	0.093179	LORNOXICAM
6.372912	-0.09143	0.103668811	10.36688114	1.743767	0.0812	0.212005	MIFEPRISTONE
6.371068	-0.02997	0.103611513	10.36115132	2.261755	0.023713	0.084546	Ispinesib (SB-715992)
6.431364	-0.21732	0.103573285	10.35732854	2.295162	0.021724	0.079334	Cephalomannine
6.440909	-0.23767	0.102669464	10.26694637	1.726957	0.084175	0.217193	Calcitriol
6.4133	-0.12768	0.102367422	10.23674216	3.933706	8.36E-05	0.000647	MENBUTONE
6.382017	-0.05457	0.102227275	10.22272747	1.933706	0.053149	0.156934	DECOQUINATE [5mM]
6.344392	0.011858	0.101985093	10.1985093	3.919014	8.89E-05	0.000685	SULFACARBAMIDE
6.414973	-0.11961	0.101932627	10.19326272	2.173882	0.029714	0.100886	MINAPRINE HYDROCHLORIDE
6.361728	-0.01141	0.101653229	10.16532294	2.219007	0.026486	0.092189	Saracatinib (AZD0530)
6.399674	-0.14899	0.1014194	10.14194002	1.705931	0.088021	0.224324	SPAGLUMIC ACID
6.40654	-0.10638	0.101282187	10.12821866	2.776131	0.005501	0.026521	PAROMOMYCIN SULFATE
6.40824	-0.10595	0.100878317	10.08783169	2.151397	0.031445	0.105572	DROPERIDOL
6.378398	-0.04453	0.100612331	10.06123309	2.196285	0.028072	0.096655	VX-680 (Tozasertib, MK-0457)
6.421604	-0.13307	0.100248649	10.02486493	2.137969	0.032519	0.107906	LINEZOLID
6.401401	-0.09382	0.100187364	10.01873641	1.89512	0.058077	0.166771	LAMOTRIGINE
6.41162	-0.17484	0.099410081	9.941008091	2.202906	0.027601	0.095507	Ligustrazine hydrochloride
6.40824	-0.16368	0.099229433	9.922943251	7.774585	7.55E-15	1.78E-13	1S,2R-PHENYLPROPANOLAMINE HYDROCHLORIDE
6.421604	-0.13473	0.099179862	9.917986219	1.876062	0.060647	0.171553	PINACIDIL
6.416641	-0.18158	0.098736683	9.873668279	7.735979	1.02E-14	2.38E-13	PROCYCLIDINE HYDROCHLORIDE
6.459392	-0.27764	0.09861675	9.861674962	2.185326	0.028865	0.098657	Methotrexate
6.439333	-0.16563	0.098424981	9.842498063	2.148537	0.031671	0.106123	AZD9291
6.378398	-0.04453	0.098341658	9.834165788	2.146718	0.031816	0.106468	Foretinib (GSK1363089)
6.354108	0.003891	0.097884886	9.788488555	2.087558	0.036838	0.119072	QUINIDINE GLUCONATE
6.382017	-0.11102	0.097661886	9.766188572	1.642727	0.100439	0.24617	ROFLUMILAST
6.450249	-0.19274	0.097459486	9.745948561	1.84352	0.065253	0.181559	SELEGILINE HYDROCHLORIDE
6.390935	-0.07263	0.097134559	9.713455906	1.837374	0.066155	0.183187	FLUVOXAMINE MALEATE
6.431364	-0.21714	0.096335158	9.633515759	1.620411	0.105144	0.252978	Afuresertib (GSK2110183)
6.419956	-0.13263	0.09617757	9.617757008	2.636214	0.008384	0.037164	QUETIAPINE FUMARATE
6.40824	-0.16368	0.096168983	9.616898258	7.5348	4.88E-14	1.09E-12	APIXABAN
6.401401	-0.1491	0.096058533	9.605853348	7.526147	5.22E-14	1.16E-12	DIMERCAPROL
6.454845	-0.21181	0.096031478	9.603147839	3.690233	0.000224	0.001602	METACETAMOL
6.330414	-9.18E-05	0.095585006	9.558500568	2.118143	0.034163	0.11234	Gastrodin
6.431364	-0.15287	0.094334815	9.433481537	2.011846	0.044236	0.137767	CYANOCOBALAMIN
6.428135	-0.14863	0.093720592	9.372059176	2.568868	0.010203	0.043936	ESCITALOPRAM OXALATE
6.421604	-0.13307	0.093676741	9.367674148	1.997812	0.045737	0.141666	AZACITIDINE
6.378398	-0.057	0.093417132	9.341713184	3.58977	0.000331	0.002268	PIDOTIMOD
6.401401	-0.1491	0.093400689	9.340068915	7.317906	2.52E-13	5.24E-12	ERGONOVINE MALEATE
6.363612	-0.01515	0.093394875	9.339487548	2.038734	0.041477	0.131167	FG-4592
6.418301	-0.12383	0.092976902	9.297690158	2.02961	0.042396	0.133498	Barasertib (AZD1152-HQPA)
6.40824	-0.11743	0.092266394	9.226639436	3.545551	0.000392	0.00265	BUCLADESINE SODIUM
6.429752	-0.15179	0.092100391	9.210039084	2.524459	0.011588	0.04867	CLOPIDOL
6.342423	-0.02587	0.091820709	9.182070943	1.544475	0.122473	0.282588	SODIUM PHENYLBUTYRATE
6.38739	-0.06545	0.091719496	9.171949594	1.734944	0.082751	0.21485	ETHOXZOLAMIDE
6.385606	-0.11873	0.091700355	9.170035464	1.542451	0.122964	0.28321	ZANAMIVIR
6.434569	-0.16099	0.09161097	9.16109697	1.732891	0.083115	0.215577	AMIODARONE HYDROCHLORIDE
6.40824	-0.16741	0.091447142	9.14471419	1.538192	0.124002	0.284321	FELODIPINE
6.403121	-0.15277	0.090139799	9.013979944	7.062416	1.64E-12	3.15E-11	NEOSTIGMINE BROMIDE
6.404834	-0.09707	0.089767754	8.976775353	1.959557	0.050048	0.150378	Pramiracetam
6.445604	-0.24776	0.089626816	8.962681628	1.507573	0.131664	0.295666	Delavirdine Mesylate
6.4133	-0.17446	0.089369618	8.936961845	7.002073	2.52E-12	4.80E-11	CHLORHEXIDINE DIHYDROCHLORIDE
6.39794	-0.09658	0.089073275	8.90732749	3.422848	0.00062	0.004011	DEQUALINIUM CHLORIDE
6.416641	-0.12468	0.088891421	8.889142109	1.681448	0.092676	0.232267	BENZOYLPAS
6.439333	-0.17063	0.088279332	8.827933185	1.66987	0.094945	0.236554	RIBOFLAVIN 5-PHOSPHATE SODIUM
6.462398	-0.22711	0.088049833	8.804983305	3.38352	0.000716	0.00458	2-THIOURACIL

6.380211	-0.10395	0.087920663	8.79206634	6.888548	5.64E-12	1.04E-10	SALSALATE
6.459392	-0.21125	0.087224051	8.722405077	1.649909	0.098962	0.244185	NOMIFENSINE MALEATE
6.403121	-0.10707	0.087154509	8.715450925	3.349115	0.000811	0.005137	DIBEKACIN
6.416641	-0.18158	0.087053945	8.705394524	6.820641	9.06E-12	1.64E-10	PHENOXYBENZAMINE HYDROCHLORIDE
6.414973	-0.12288	0.086992785	8.69927853	2.38446	0.017104	0.066193	PHYTONADIONE [5mM]
6.414973	-0.13107	0.086803043	8.680304318	3.335609	0.000851	0.00538	DIACERIN
6.404834	-0.16008	0.086797093	8.679709266	1.459975	0.144297	0.314379	CEFTEZOLE
6.367356	-0.07959	0.086601852	8.660185238	1.919079	0.054974	0.160107	Celiprolol hydrochloride
6.38739	-0.06891	0.086453341	8.645334102	2.369674	0.017804	0.068026	BROMHEXINE HYDROCHLORIDE
6.416641	-0.18158	0.086341311	8.634131081	6.764806	1.33E-11	2.37E-10	FIROCOXIB
6.409933	-0.17105	0.085772462	8.577246159	1.44274	0.149094	0.321176	Tolvaptan
6.462398	-0.28388	0.0856815	8.568150012	1.44121	0.149525	0.321803	Succinylcholine Chloride·2H2O
6.330414	0.05197	0.085627165	8.562716523	1.826141	0.067829	0.186456	PROPANTHELINE BROMIDE
6.40654	-0.10046	0.084945489	8.494548939	1.854291	0.063698	0.178214	Triciribine
6.419956	-0.12972	0.084520739	8.452073908	1.802545	0.07146	0.19359	ASCORBIC ACID
6.385606	-0.11886	0.084497224	8.44972239	1.872441	0.061146	0.172678	Acetylspiramycin
6.426511	-0.14467	0.084150131	8.415013056	1.591763	0.111438	0.265254	DEHYDROACETIC ACID
6.389166	-0.12639	0.083828049	8.38280488	1.410034	0.15853	0.335786	CARBAZOCHROME
6.4133	-0.12768	0.083299129	8.329912924	3.200963	0.00137	0.008164	BRONOPOL
6.419956	-0.1314	0.083113208	8.311320825	1.572149	0.115916	0.272618	PENBUTOLOL SULFATE
6.416641	-0.123	0.082933263	8.293326269	1.768689	0.076946	0.203809	MEBEVERINE HYDROCHLORIDE
6.466868	-0.23616	0.08284214	8.284213986	3.183402	0.001456	0.008616	TIOCONAZOLE
6.356026	-0.00755	0.081394099	8.13940993	2.231001	0.025681	0.089875	BETAINE HYDROCHLORIDE
6.445604	-0.18333	0.081350807	8.135080674	1.538812	0.12385	0.284228	TACRINE HYDROCHLORIDE
6.414973	-0.17803	0.081202368	8.120236772	6.362172	1.99E-10	3.30E-09	PROBENECID
6.409933	-0.17121	0.081149653	8.114965341	1.798259	0.072136	0.194798	Baicalein
6.424882	-0.2032	0.080577554	8.057755372	1.355359	0.175303	0.358511	Warfarin·Na
6.419956	-0.18864	0.080357232	8.035723218	6.295956	3.06E-10	5.03E-09	PHENYLBUTAZONE
6.40654	-0.16006	0.079750066	7.975006618	6.248385	4.15E-10	6.74E-09	DICLOXACILLIN SODIUM
6.418301	-0.12805	0.079381559	7.938155927	1.501562	0.13321	0.297702	ZINC UNDECYLENATE [4mM]
6.409933	-0.16729	0.079337708	7.933770799	6.216077	5.10E-10	8.23E-09	CLOXYQUIN
6.436163	-0.16261	0.079007795	7.900779495	1.684972	0.091994	0.23143	MARBOFLOXACIN
6.426511	-0.14302	0.078064064	7.806406374	1.664846	0.095944	0.23823	TRYPTOPHAN (L)
6.394452	-0.13776	0.078050789	7.805078901	1.312858	0.189231	0.37655	Azelnidipine
6.367356	-0.03464	0.077957908	7.795790849	2.995714	0.002738	0.014726	CHLORMIDAZOLE
6.419956	-0.12712	0.077939433	7.793943252	1.701354	0.088876	0.225718	Poziotinib (HM781-36B)
6.40654	-0.16006	0.077692091	7.769209119	6.087143	1.15E-09	1.78E-08	CEFADROXIL
6.414973	-0.12131	0.077555096	7.755509607	1.467013	0.142372	0.311894	DISOPYRAMIDE PHOSPHATE
6.454845	-0.19645	0.077547761	7.754776083	1.692805	0.090493	0.228801	Prucalopride
6.431364	-0.21732	0.077207583	7.720758273	1.710904	0.087099	0.223085	Clinofibrate
6.423246	-0.1364	0.077124572	7.712457221	1.644809	0.100009	0.245771	BENZOIC ACID
6.409933	-0.12086	0.076342918	7.634291822	2.933654	0.00335	0.017538	CLOFIBRIC ACID
6.434569	-0.21978	0.076069295	7.606929517	5.959998	2.52E-09	3.72E-08	METHOXAMINE HYDROCHLORIDE
6.394452	-0.08951	0.074988678	7.498867766	2.881614	0.003956	0.020136	ACEGLUTAMIDE
6.450249	-0.25796	0.074834977	7.48349765	1.658327	0.097251	0.240428	Phenylephrine
6.414973	-0.17803	0.074825221	7.482522137	5.862525	4.56E-09	6.48E-08	BROMOCRIPTINE MESYLATE
6.403121	-0.10707	0.074274595	7.427459469	2.854174	0.004315	0.021702	SODIUM CYCLAMATE
6.392697	-0.13398	0.073742255	7.374225515	1.240386	0.214833	0.410274	ASENAPINE MALEATE
6.426511	-0.2067	0.073438121	7.343812082	1.23527	0.21673	0.412028	Varenicline Tartrate
6.436163	-0.16434	0.072638303	7.263830335	1.991006	0.04648	0.143274	NITAZOXANIDE
6.421604	-0.13585	0.072325178	7.232517785	1.982423	0.047432	0.145594	LEVOCARNITINE
6.396199	-0.09305	0.072102622	7.210262189	2.770711	0.005593	0.02684	ALOIN
6.424882	-0.19914	0.071837115	7.183711499	5.628408	1.82E-08	2.44E-07	DIETHYLCARBAMAZINE CITRATE
6.426511	-0.14302	0.071555353	7.155535319	1.526037	0.127001	0.288741	ONDANSETRON HYDROCHLORIDE
6.432969	-0.15298	0.071478664	7.147866396	1.560321	0.118684	0.276129	Alisertib (MLN8237)
6.389166	-0.12303	0.071419836	7.141983588	5.595715	2.20E-08	2.91E-07	CLOTRIMAZOLE
6.416641	-0.18158	0.071381379	7.138137867	5.592702	2.24E-08	2.95E-07	OXACILLIN SODIUM
6.444045	-0.24441	0.070860625	7.086062501	1.191915	0.233295	0.430129	PRAZOSIN HYDROCHLORIDE [10mg]
6.429752	-0.15123	0.070644955	7.064495513	1.336303	0.18145	0.365183	PIRENPERONE
6.450249	-0.19274	0.070225841	7.022584093	1.328375	0.184054	0.368884	PENTYLENETETRAZOL
6.423246	-0.19565	0.069556004	6.955600411	5.449684	5.05E-08	6.38E-07	ESTRADIOL
6.546543	-0.33211	0.06948618	6.948618049	3.792441	0.000149	0.001107	Dacarbazine
6.434569	-0.15937	0.069239777	6.923977702	1.476653	0.139769	0.307263	FENBENDAZOLE
6.40824	-0.10384	0.069205868	6.92058679	1.510708	0.130863	0.295292	Uprosertib (GSK2141795)
6.419956	-0.1314	0.069083511	6.908351051	1.306767	0.191292	0.379618	CREATININE
6.450249	-0.2025	0.068926847	6.89268472	2.648674	0.008081	0.036009	gamma-AMINOBUTYRIC ACID HYDROCHLORIDE
6.41162	-0.17088	0.068811629	6.881162942	5.391363	6.99E-08	8.71E-07	PRIMAQUINE PHOSPHATE
6.451786	-0.19585	0.06852922	6.852921959	1.296282	0.194878	0.384796	OXANTEL PAMOATE
6.445604	-0.18333	0.068451159	6.845115918	1.294805	0.195387	0.385504	AMOXAPINE
6.401401	-0.09207	0.068117479	6.811747893	1.452718	0.146302	0.316998	ALBUTEROL
6.448706	-0.25464	0.06795027	6.795026986	1.505764	0.132128	0.296578	Tanshinone I
6.390935	-0.06945	0.067748911	6.774891143	1.478904	0.139166	0.306156	Eltrombopag
6.419956	-0.12972	0.067647177	6.764717732	1.442688	0.149108	0.321176	BUTAMBEN
6.4133	-0.11792	0.06691008	6.691008024	1.265655	0.205637	0.397756	IRINOTECAN HYDROCHLORIDE
6.432969	-0.15298	0.066531385	6.65313854	1.452326	0.146411	0.317002	Mecarbinate
6.418301	-0.18512	0.066432871	6.643287119	5.204988	1.94E-07	2.32E-06	NADIDE
6.457882	-0.21796	0.066195135	6.619513488	2.543702	0.010968	0.046799	PYRETHRINS
6.444045	-0.17976	0.066083533	6.608353294	1.81134	0.070088	0.191694	PEMIROLAST POTASSIUM
6.440909	-0.23786	0.066045249	6.60452492	1.463549	0.143317	0.313523	Psoralen
6.332438	0.046799	0.065947872	6.594787222	1.439588	0.149984	0.322519	Linifanib (ABT-869)
6.465383	-0.29053	0.065934591	6.593459073	1.461097	0.143989	0.314113	Tabersonine
6.4133	-0.11961	0.065685665	6.568566522	1.800435	0.071792	0.194073	CIPROFLOXACIN
6.456366	-0.27112	0.065670372	6.567037162	1.455242	0.145602	0.315883	Hydroxocobalamin·HCl
6.44248	-0.17543	0.065651096	6.565109643	1.400119	0.161478	0.338646	VORICONAZOLE
6.429752	-0.21367	0.065619906	6.561990612	1.103763	0.269696	0.471922	LUMEFANTRINE
6.451786	-0.19038	0.065560194	6.556019418	1.431126	0.152394	0.326331	BAF312 (Siponimod)
6.403121	-0.10707	0.065464461	6.546446121	2.515624	0.011882	0.049582	PYRONARIDINE TETRAPHOSPHATE
6.444045	-0.17976	0.06544685	6.544685002	1.793889	0.072831	0.196157	PACLITAXEL
6.414973	-0.12131	0.065440786	6.544078631	1.237862	0.215767	0.410832	RETINYL PALMITATE
6.447158	-0.18648	0.065126697	6.512669724	1.231921	0.217979	0.413404	ERYTHRITOL
6.419956	-0.13263	0.064651165	6.465116472	1.772079	0.076381	0.202839	CLAVULANATE LITHIUM
6.404834	-0.10304	0.064625733	6.462573322	1.771382	0.076497	0.202944	MEPHENYTOIN
6.463893	-0.22037	0.064550074	6.455007416	1.221013	0.222081	0.417579	ACETOHYDROXAMIC ACID
6.445604	-0.17809	0.064526483	6.452648343	1.408561	0.158965	0.336323	TH-302
6.429752	-0.14659	0.064308501	6.430850112	1.403802	0.160378	0.33736	Ledipasvir (GS5885)
6.459392	-0.21125	0.064271346	6.427134648	1.215741	0.224084	0.419338	SILDENAFIL CITRATE
6.4133	-0.17446	0.064213056	6.421305633	5.031067	4.88E-07	5.55E-06	MICONAZOLE NITRATE
6.389166	-0.12639	0.063734802	6.373480202	1.072055	0.283695	0.489064	NICOPHOLINE
6.394452	-0.13776	0.063692073	6.369207264	1.071336	0.284018	0.489347	ACEFYLLINE
6.421604	-0.19215	0.06238381	6.238380973	4.887746	1.02E-06	1.11E-05	SODIUM NITROPRUSSIDE DIHYDRATE
6.431364	-0.1545	0.062361329	6.236132903	1.179612	0.238155	0.435327	PRAVASTATIN SODIUM
6.424882	-0.13691	0.061594352	6.159435202	1.344555	0.178769	0.362207	LY2835219
6.429752	-0.15179	0.061457905	6.145790468	1.684553	0.092075	0.23143	KETOROLAC TROMETHAMINE
6.426511	-0.14015	0.061297437	6.129743659	1.338073	0.180873	0.364737	Lomitapide
6.440909	-0.23786	0.061027413	6.102741281	1.352355	0.176262	0.359721	Ulipristal acetate
6.450249	-0.25796	0.061004063	6.100406321	1.351837	0.176427	0.359721	Etofenamate
6.44248	-0.17543	0.060961346	6.09613458	1.300102	0.193566	0.383092	TRICHLORMETHIAZIDE
6.44248	-0.17669	0.060814916	6.081491604	1.666928	0.095529	0.23761	COLFORSIN
6.423246	-0.1364	0.060625356	6.062535607	1.292936	0.196033	0.386127	CEFMENOXIME HYDROCHLORIDE
6.409933	-0.1111	0.06050506	6.050505972	1.144499	0.252417	0.452638	ANIRACETAM
6.429752	-0.161	0.060426761	6.042676101	2.322039	0.020231	0.075254	ZOXAZOLAMINE
6.472756	-0.23832	0.060382449	6.038244929	1.14218	0.253379	0.453444	LOPERAMIDE HYDROCHLORIDE
6.424882	-0.14227	0.059920198	5.992019774	1.642404	0.100506	0.246216	LAMIVUDINE
6.401401	-0.09025	0.059781663	5.978166335	1.304985	0.191898	0.380443	Pimasertib (AS-703026)
6.40654	-0.1025	0.059687578	5.968757814	1.272937	0.203041	0.394383	XYLOMETAZOLINE HYDROCHLORIDE
6.428135	-0.21019	0.059675432	5.967543247	1.003774	0.315488	0.521568	TRIADIMEFON
6.431364	-0.15495	0.059039241	5.903924097	1.618257	0.105607	0.253616	SULFANITRAN
6.421604	-0.1304	0.05859508	5.85950803	1.279083	0.200868	0.391786	IPI-145 (INK1197)
6.432969	-0.22078	0.058585707	5.858570707	1.298247	0.194203	0.383992	Chloral hydrate
6.426511	-0.15443	0.058577804	5.857780355	2.250988	0.024386	0.086347	MEXENEONE
6.421604	-0.19632	0.058544284	5.854428394	1.297329	0.194518	0.384381	Estramustine Phosphate·Na
6.448706	-0.25443	0.058417912	5.841791223	0.982622	0.325794	0.531906	DETOMIDINE HYDROCHLORIDE
6.466868	-0.23616	0.058383438	5.838343807	2.243519	0.024863	0.087612	DOCONEXENT
6.421604	-0.19632	0.058172025	5.817202529	1.28908	0.19737	0.387624	Crocin
6.432969	-0.16751	0.05794634	5.794633954	2.226723	0.025966	0.090809	PRONETALOL HYDROCHLORIDE
6.451786	-0.19038	0.05793758	5.793758003	1.26473	0.205968	0.397796	Varlitinib
6.4133	-0.17829	0.057772859	5.777285858	0.971772	0.331164	0.53634	FLUPIRTINE MALEATE
6.468347	-0.29667	0.057462566	5.746256571	0.966552	0.333768	0.537727	Alosetron·HCl
6.38739	-0.07521	0.05734017	5.734016987	2.203429	0.027565	0.095487	RUTIN
6.429752	-0.21385	0.057264928	5.726492755	1.268979	0.204449	0.395907	Iopromide
6.474216	-0.24127	0.057253734	5.725373362	1.082998	0.278809	0.482754	BISOPROLOL FUMARATE

6.457882	-0.27438	0.057241041	5.724104091	1.268449	0.204638	0.395973	Bulleyaconi cine A
6.39794	-0.08505	0.057239468	5.723946794	1.220727	0.22219	0.417608	SCOPOLAMINE HYDROBROMIDE
6.456366	-0.20386	0.057234522	5.723452236	1.56879	0.116697	0.273325	NICLOSAMIDE
6.434569	-0.15937	0.057132568	5.713256817	1.218447	0.223054	0.418335	AVANAFIL
6.434569	-0.15616	0.056972866	5.697286599	1.243671	0.213621	0.408874	CO-1686 (AVL-301)
6.431364	-0.16426	0.056964995	5.696499492	2.189012	0.028596	0.097934	CINCHONINE
6.39794	-0.14173	0.056805805	5.680580475	4.450711	8.56E-06	8.06E-05	ALLOPURINOL
6.423246	-0.1364	0.05627471	5.627471025	1.200152	0.23008	0.426971	CEFACLOR
6.365488	-0.07557	0.056209012	5.620901184	1.24558	0.212919	0.408019	Daphnetin
6.439333	-0.23447	0.056159559	5.615955867	1.244484	0.213322	0.408607	Chitosan
6.489958	-0.28292	0.056079594	5.607959371	2.154989	0.031163	0.104831	ETEBENECID
6.428135	-0.20607	0.055927277	5.592727652	4.381879	1.18E-05	0.000109	METHACHOLINE CHLORIDE
6.444045	-0.24461	0.055908861	5.590886078	1.238928	0.215372	0.410577	Methyl salicylate
6.419956	-0.1926	0.055740582	5.574058212	0.937588	0.348456	0.550817	DIDANOSINE
6.41162	-0.17088	0.055713863	5.571386334	4.365158	1.27E-05	0.000116	ALVERINE CITRATE
6.428135	-0.20607	0.055638235	5.563823514	4.359233	1.31E-05	0.000119	ERYTHROMYCIN
6.436163	-0.22746	0.055471446	5.547144617	0.933061	0.350789	0.552916	BICALUTAMIDE
6.39794	-0.08682	0.055245372	5.524537163	1.045008	0.296019	0.500535	PANTHENOL (dl)
6.437751	-0.16744	0.055116528	5.511652795	1.510736	0.130856	0.295292	FLUVASTATIN SODIUM
6.451786	-0.1949	0.055008783	5.500878287	1.507783	0.13161	0.295666	SIMVASTATIN
6.456366	-0.20386	0.054907275	5.490727548	1.505001	0.132324	0.296758	NONOXYNOL-9
6.460898	-0.2143	0.05482908	5.48290802	1.037134	0.299674	0.50405	PRALIDOXIME MESYLATE
6.474216	-0.24127	0.054757257	5.475725699	1.035775	0.300307	0.504453	PENTETIC ACID
6.434569	-0.21978	0.054736851	5.473685145	4.28861	1.80E-05	0.000161	PROCHLORPERAZINE EDISYLATE
6.456366	-0.21489	0.054657362	5.465736186	2.100336	0.035699	0.116051	PROFLAVINE HEMISULFATE
6.403121	-0.09556	0.054607255	5.460725544	1.16459	0.244185	0.443076	beta-CAROTENE [2mM]
6.41162	-0.11055	0.054583015	5.458301465	1.191503	0.233456	0.430272	Amonafide
6.419956	-0.12712	0.05448603	5.448603008	1.189386	0.234288	0.431494	BYL719
6.356026	-0.05521	0.054484211	5.448421148	1.207359	0.227294	0.424106	Retigabine
6.436163	-0.16422	0.054354216	5.435421598	1.028151	0.303879	0.508119	PIPAMPERONE
6.432969	-0.15298	0.053981715	5.398171546	1.178377	0.238646	0.435825	Masitinib (AB1010)
6.421604	-0.19215	0.053851623	5.385162288	4.219253	2.45E-05	0.000214	PHENELZINE SULFATE
6.416641	-0.18158	0.053782451	5.378245103	4.213833	2.51E-05	0.000219	CHLORZOXAZONE
6.439333	-0.16904	0.053715003	5.371500278	1.145562	0.251977	0.45232	SULOCTIDIL
6.359835	-0.0634	0.053655702	5.3655702	1.188999	0.23444	0.431619	MK3102
6.488551	-0.28006	0.053552462	5.355246192	2.057878	0.039602	0.126554	TRICHLOROETHYLENE
6.4843	-0.27146	0.053508991	5.350899076	2.056207	0.039763	0.126844	DOCETAXEL
6.423246	-0.19985	0.05350621	5.350621007	1.185686	0.235746	0.433266	Fingolimod
6.423246	-0.19985	0.05350366	5.350365984	1.18563	0.235769	0.433266	Eflornithine·HCl
6.450249	-0.25796	0.053214005	5.321400461	1.179211	0.238314	0.435463	17-Methyltestosterone
6.462398	-0.22711	0.053082788	5.308278774	2.03983	0.041367	0.130903	RETINYL ACETATE
6.439333	-0.22993	0.05295062	5.295062004	4.148659	3.34E-05	0.000283	METHOCARBAMOL
6.436163	-0.15933	0.05277576	5.277575954	1.152052	0.2493	0.449284	Tianeptine sodium
6.436163	-0.16434	0.052696198	5.269619801	1.444395	0.148628	0.320545	BETAZOLE HYDROCHLORIDE
6.354108	-0.05108	0.052517842	5.251784232	1.163784	0.244511	0.443076	Osalmid
6.436163	-0.22765	0.052295706	5.229570565	1.158862	0.246513	0.445515	Dihydralazine sulphate
6.463893	-0.22037	0.052244286	5.224428577	0.98824	0.323035	0.529879	ACECAINIDE HYDROCHLORIDE
6.431364	-0.21295	0.052225387	5.222538725	4.091838	4.28E-05	0.000353	SODIUM OXYBATE
6.432969	-0.21637	0.052156352	5.215635156	4.086429	4.38E-05	0.00036	NYSTATIN
6.38739	-0.1227	0.051912175	5.191217486	1.150363	0.249994	0.45006	Dicurone
6.41162	-0.11055	0.051748948	5.174894846	1.129637	0.258629	0.458931	Crizotinib (PF-02341066)
6.4843	-0.33123	0.051695372	5.169537164	1.145558	0.251978	0.45232	Arctigenin
6.445604	-0.18281	0.051657701	5.165770056	1.41593	0.156796	0.332924	TYLOSIN TARTRATE
6.428135	-0.15772	0.051608625	5.160862547	1.983181	0.047347	0.14546	CYPERMETHRIN
6.453318	-0.26435	0.051391248	5.139124753	0.864429	0.387352	0.584773	Anagrelide
6.44248	-0.17543	0.051347108	5.134710767	1.095062	0.273489	0.476119	MEROPENEM
6.453318	-0.19895	0.051195039	5.119503907	0.968393	0.332848	0.537428	CLONAZEPAM
6.444045	-0.17976	0.051072862	5.107286193	1.3999	0.161543	0.338646	BIFONAZOLE
6.460898	-0.22407	0.050904032	5.090403158	1.956106	0.050453	0.151329	CLENBUTEROL HYDROCHLORIDE
6.423246	-0.19565	0.050654058	5.065405824	3.968725	7.23E-05	0.000566	MEQUINOL
6.440909	-0.17383	0.050597124	5.059712434	0.957083	0.338525	0.541979	GABOXADOL HYDROCHLORIDE
6.4133	-0.17829	0.050400654	5.040065411	0.847767	0.396568	0.593692	GLUTAMINE (L) HYDROCHLORIDE
6.477121	-0.31579	0.050272975	5.027297509	1.114038	0.265263	0.466556	Eupatilin
6.4133	-0.11961	0.049998142	4.999814218	1.370442	0.170549	0.352527	CLOFIBRATE
6.480007	-0.253	0.049945606	4.994560564	0.944759	0.344782	0.547105	ACTINOQUINOL SODIUM
6.429752	-0.20952	0.049821728	4.982172767	3.903512	9.48E-05	0.000723	NAPROXEN
6.423246	-0.14782	0.049757691	4.975769139	1.912055	0.055869	0.161976	HYDRASTININE HYDROCHLORIDE
6.477121	-0.31579	0.049752223	4.975222252	1.102499	0.270245	0.472721	Levothyroxine·Na
6.439333	-0.16563	0.049016912	4.901691174	1.069999	0.28462	0.489668	Tideglusib
6.385606	-0.11873	0.048998357	4.899835655	0.82418	0.409838	0.60559	ACENEURAMIC ACID
6.41162	-0.11632	0.048924123	4.892412332	1.341003	0.179919	0.363674	PENTOXIFYLLINE
6.450249	-0.19189	0.04878337	4.878337018	1.337145	0.181175	0.365086	LEVALBUTEROL HYDROCHLORIDE
6.352183	-0.04694	0.048551691	4.855169058	1.075895	0.281974	0.487152	Alvimopan
6.421604	-0.1445	0.048476674	4.847667401	1.862829	0.062486	0.175593	MECLOFENOXATE HYDROCHLORIDE
6.426511	-0.14467	0.048222133	4.822213292	0.912158	0.361685	0.562695	PREDNISOLONE TEBUTATE
6.488551	-0.34012	0.048171412	4.817141199	0.81027	0.417785	0.612385	Toremifene Base
6.454845	-0.21181	0.047740799	4.774079891	1.834551	0.066572	0.183768	PICOLAMINE
6.457882	-0.27438	0.047669789	4.766978925	1.056352	0.290807	0.494827	Gemifloxacin
6.462398	-0.21566	0.047518486	4.751848636	1.302475	0.192754	0.38178	CARBADOX
6.437751	-0.16583	0.047513432	4.751343206	1.013303	0.310916	0.516679	SULINDAC
6.39794	-0.08337	0.047369445	4.736944505	1.034036	0.301119	0.50532	Mosapride Citrate
6.40824	-0.16741	0.046814492	4.681449197	0.787446	0.431021	0.624633	CLINDAMYCIN PALMITATE HYDROCHLORIDE
6.419956	-0.13263	0.046723446	4.672344583	1.280683	0.200305	0.391448	TYROSINE HYDROCHLORIDE
6.424882	-0.15113	0.046596893	4.659689284	1.790594	0.073358	0.196956	DIMESNA
6.414973	-0.13107	0.046556267	4.655626662	1.789033	0.07361	0.197527	ACETYL-L-LEUCINE
6.40824	-0.10595	0.046540072	4.654007191	0.992544	0.320932	0.528293	DICUMAROL
6.448706	-0.25443	0.046463491	4.646349095	0.781542	0.434484	0.626732	ZONISAMIDE
6.475671	-0.31266	0.046423173	4.642317329	1.028728	0.303608	0.507842	Alectinib
6.471292	-0.23535	0.04619454	4.619453996	0.873805	0.382225	0.581745	SULFADOXINE
6.409933	-0.16729	0.046129346	4.612934606	3.614215	0.000301	0.00209	CYCLOPENTOLATE HYDROCHLORIDE
6.429752	-0.20952	0.045960955	4.596095465	3.601022	0.000317	0.002184	METAPROTERENOL
6.439333	-0.23447	0.045927264	4.592726427	1.017738	0.308802	0.513834	Cholic Acid
6.444045	-0.1786	0.045510175	4.551017509	0.97058	0.331757	0.536512	CYCLOHEXIMIDE
6.447158	-0.18585	0.045201126	4.52011256	1.238957	0.215362	0.410577	FLUCYTOSINE
6.424882	-0.14137	0.045148283	4.514828305	0.854014	0.393097	0.590569	BISMUTH SUBSALICYLATE
6.399674	-0.08681	0.045125878	4.512587833	0.985061	0.324594	0.531439	FT-207 (NSC 148958)
6.40824	-0.16368	0.044950949	4.495094891	3.521888	0.000428	0.002875	CHLORTHALIDONE
6.418301	-0.18905	0.044800093	4.480009338	0.753562	0.451112	0.639857	D-ERYTHOBIC ACID
6.416641	-0.12614	0.044611546	4.461154612	1.222796	0.221407	0.417157	ARTEMETHER
6.44248	-0.17543	0.044523275	4.452327491	0.949533	0.34235	0.545215	PROGLUMIDE
6.382017	-0.06433	0.044443097	4.444309737	1.707829	0.087668	0.223912	IOVERSOL
6.489958	-0.28292	0.04407698	4.407697953	1.69376	0.090311	0.228567	SULFANILAMIDE
6.454845	-0.20089	0.044055092	4.405509234	1.207544	0.227223	0.424106	TORSEMIDE
6.434569	-0.15937	0.044047225	4.404722511	0.93938	0.347536	0.550288	FLUBENDAZOLE
6.456366	-0.2036	0.043867214	4.386721423	0.935541	0.349509	0.55163	TETRACYCLINE HYDROCHLORIDE
6.456366	-0.21489	0.043837952	4.383795199	1.684575	0.092071	0.23143	HEXAMETHONIUM BROMIDE
6.437751	-0.16583	0.043710499	4.371049923	0.932199	0.351234	0.552988	TOLAZAMIDE
6.409933	-0.1111	0.043427215	4.342721471	0.821459	0.411385	0.607373	TERBINAFINE HYDROCHLORIDE
6.4133	-0.17829	0.043301835	4.330183513	0.728361	0.466393	0.652618	CILNIDIPINE
6.453318	-0.19742	0.043196159	4.319615877	0.92123	0.35693	0.557814	BUMETANIDE
6.445604	-0.17809	0.042671895	4.267189526	0.931493	0.351599	0.553392	Eliglustat Tartrate
6.437751	-0.16744	0.042602723	4.260272257	1.167735	0.242914	0.441818	METHAPYRILENE HYDROCHLORIDE
6.447158	-0.18492	0.042497637	4.249763707	0.906333	0.36476	0.565989	SULFABENZAMIDE
6.447158	-0.2511	0.042069805	4.206980515	0.707637	0.47917	0.664505	CLINAFOXACIN HYDROCHLORIDE
6.432969	-0.15775	0.041938305	4.193830528	0.793295	0.427606	0.62109	CHLOROPHYLLIDE Cu COMPLEX Na SALT
6.477121	-0.24072	0.041812647	4.181264659	0.912736	0.361381	0.562564	Bindarit
6.480007	-0.32199	0.041728545	4.172854467	0.924696	0.355124	0.556716	Nitisinone
6.447158	-0.18585	0.041721267	4.172126662	1.143574	0.2528	0.452638	PANTOPRAZOLE
6.445604	-0.18177	0.04171114	4.171114004	0.889559	0.373703	0.575151	TERBUTALINE HEMISULFATE
6.469822	-0.23237	0.041562946	4.156294625	0.786195	0.431754	0.624829	IOTHALAMIC ACID
6.457882	-0.2082	0.041464224	4.146422394	0.784327	0.432848	0.625687	VECURONIUM BROMIDE
6.448706	-0.18806	0.040849714	4.084971351	0.871188	0.383652	0.582262	TIMOLOL MALEATE
6.451786	-0.26127	0.0406103	4.061030015	0.899916	0.368165	0.569316	Genipin
6.440909	-0.18359	0.040432614	4.043261405	1.553717	0.120252	0.278719	ACEMETACIN
6.389166	-0.12652	0.040422104	4.042210404	0.895745	0.370389	0.571374	Isoliquiritigenin
6.439333	-0.23447	0.04027148	4.027148021	0.892407	0.372175	0.573438	Pancreatin
6.451786	-0.19431	0.040234954	4.02349545	0.858077	0.39085	0.587882	TRIFLUOPERAZINE HYDROCHLORIDE
6.468347	-0.23915	0.040093158	4.009315844	1.540673	0.123396	0.284028	CLIMBAZOLE
6.462398	-0.22711	0.040021177	4.002117725	1.537907	0.124071	0.284341	GLAFENINE
6.482874	-0.2588	0.039993935	3.999393488	0.756516	0.44934	0.638835	DULOXETINE HYDROCHLORIDE
6.450249	-0.19274	0.039485962	3.948596228	0.746907	0.45512	0.643172	GABAPENTIN

6.392697	-0.0793	0.039244464	3.924446448	1.075685	0.282068	0.487152	BIOTIN
6.494155	-0.29141	0.039148854	3.91488545	1.504386	0.132482	0.296983	BENFOTIAMINE
6.474216	-0.23495	0.039140907	3.914090698	0.854414	0.392876	0.590409	LDE225 (NVP-LDE225,Erismodegib)
6.447158	-0.18118	0.039007511	3.900751122	0.851502	0.39449	0.591793	Trelagliptin
6.453318	-0.26435	0.038981104	3.898110444	0.655684	0.512028	0.692624	Dinoprostone
6.385606	-0.06543	0.038861898	3.886189751	1.065199	0.286786	0.491196	SPIRAMYCIN
6.453318	-0.1979	0.038857937	3.885793708	1.065091	0.286835	0.491196	ENROFLOXACIN
6.478566	-0.25985	0.038729116	3.872911607	1.488256	0.136683	0.302036	ETHAVERINE HYDROCHLORIDE
6.457882	-0.20668	0.038295616	3.829561626	0.816718	0.41409	0.609411	LISINOPRIL
6.448706	-0.19938	0.038030111	3.803011053	1.461395	0.143907	0.314113	CHROMOCARB
6.448706	-0.18888	0.03796725	3.796724989	1.040677	0.298025	0.502256	ASPARTAME
6.444045	-0.1786	0.037398449	3.739844865	0.797584	0.425112	0.61852	SALICYL ALCOHOL
6.447158	-0.18585	0.037341819	3.734181934	1.023534	0.306055	0.510328	TELENZEPINE HYDROCHLORIDE
6.431364	-0.16426	0.036902538	3.690253841	1.418066	0.156172	0.332391	BORNYL ACETATE
6.357935	-0.05931	0.03645524	3.645524011	0.80784	0.419182	0.613536	Formononetin
6.44248	-0.17543	0.035925831	3.592583083	0.766178	0.44357	0.635064	SALICIN
6.424882	-0.2032	0.035912868	3.591286763	0.604074	0.545794	0.720781	Dexmedetomidine
6.451786	-0.26106	0.035867762	3.586776196	0.603316	0.546299	0.720889	Nedaplatin
6.440909	-0.23329	0.035827151	3.582715141	2.807043	0.005	0.024404	DIENESTROL
6.440909	-0.23786	0.035662095	3.566209456	0.790264	0.429373	0.62295	Solithromycin
6.445604	-0.18177	0.035508526	3.550852645	0.757278	0.448883	0.638767	PROPOXYCAINE HYDROCHLORIDE
6.4843	-0.33098	0.035377896	3.537789563	0.595076	0.551793	0.725669	Ticagrelor
6.440909	-0.23329	0.035153201	3.515320137	2.754239	0.005883	0.027928	PHENIRAMINE MALEATE
6.463893	-0.21859	0.035121225	3.51212252	0.962668	0.335714	0.539331	CROTAMITON
6.459392	-0.20974	0.035086718	3.508671767	0.748282	0.45429	0.64243	MONTELUKAST SODIUM
6.436163	-0.15933	0.035079143	3.50791429	0.765749	0.443826	0.635064	Lercanidipine (hydrochloride)
6.480007	-0.253	0.034976269	3.497626866	0.661603	0.508226	0.690217	TRIMETHADIONE
6.495544	-0.29423	0.034923274	3.492327364	1.342008	0.179593	0.363159	CEFALONIUM
6.44248	-0.23664	0.034918521	3.491852054	2.735852	0.006222	0.029172	METHICILLIN SODIUM
6.374748	-0.03728	0.034861002	3.486100234	0.760987	0.446665	0.637559	LEE011
6.426511	-0.14015	0.034823882	3.482388227	0.760177	0.447149	0.638072	Ziprasidone HCl
6.440909	-0.23786	0.034584955	3.458495472	0.766395	0.443441	0.635064	4-Aminoantipyrine
6.431364	-0.15287	0.034574284	3.457428427	0.737354	0.460907	0.647242	SULCONAZOLE NITRATE
6.456366	-0.21489	0.034545536	3.454553557	1.327493	0.184346	0.36898	ACETARSOL
6.451786	-0.1949	0.034188692	3.418869226	0.937107	0.348703	0.551037	ORLISTAT
6.423246	-0.13366	0.034102396	3.41023962	0.744428	0.456618	0.643455	Belinostat (PXD101)
6.462398	-0.2841	0.033963202	3.396320191	0.752617	0.45168	0.640255	Bedaquiline(TMC207; R207910)
6.451786	-0.19431	0.033907961	3.390796133	0.723144	0.469592	0.65584	SPIPERONE
6.44248	-0.23664	0.033882612	3.388261246	2.654689	0.007938	0.035586	DIPHENHYDRAMINE HYDROCHLORIDE
6.462398	-0.2841	0.033733424	3.373342395	0.747526	0.454746	0.642822	Salifungin
6.460898	-0.2128	0.033297992	3.329799153	0.710135	0.47762	0.663125	TOLAZOLINE HYDROCHLORIDE
6.447158	-0.18492	0.033295218	3.329521835	0.710076	0.477657	0.663125	FLUFENAMIC ACID
6.440909	-0.23329	0.033255189	3.325518901	2.60553	0.009173	0.040177	CHLOROCRESOL
6.469822	-0.24214	0.033181542	3.318154207	1.275078	0.202282	0.393347	IDEBENONE
6.445604	-0.24776	0.033171586	3.317158625	0.557964	0.576869	0.745098	TREHALOSE DIHYDRATE
6.495544	-0.29423	0.032862152	3.286215152	1.262805	0.206659	0.39853	NIMUSTINE HYDROCHLORIDE
6.494155	-0.29141	0.032786292	3.278629174	1.259889	0.207709	0.400102	BUFLOMEDIL HYDROCHLORIDE
6.451786	-0.19431	0.032632126	3.2632126	0.695934	0.48647	0.671174	SULFAMETHIZOLE
6.434569	-0.16122	0.032319804	3.231980386	0.885881	0.375681	0.57624	INAMRINONE
6.432969	-0.22078	0.032286593	3.228659285	0.715464	0.474322	0.66082	TAK-438(Vonoprazan fumarate)
6.465383	-0.2903	0.031964634	3.196463432	0.537663	0.59081	0.755104	NILVADIPINE
6.447158	-0.2511	0.031943059	3.194305938	0.5373	0.59106	0.755236	ZALTOPROFEN
6.454845	-0.26763	0.03188439	3.188439019	0.536313	0.591742	0.75573	Mirabegron
6.445604	-0.18281	0.031883139	3.188313889	0.873912	0.382166	0.581745	LANSOPRAZOLE
6.444045	-0.17976	0.031823258	3.18232578	0.872271	0.383061	0.582152	SODIUM GLUCONATE
6.481443	-0.25591	0.031778468	3.177846809	0.601114	0.547764	0.721707	ETHYL VANILLIN
6.44248	-0.18677	0.031557118	3.155711848	1.212656	0.225262	0.421234	LOMERIZINE HYDROCHLORIDE
6.477121	-0.25692	0.031339791	3.133979056	1.204304	0.228472	0.4251	PRASUGREL
6.448706	-0.18888	0.031297407	3.129740651	0.857858	0.390971	0.587892	BLEOMYCIN (bleomycin B2 shown)
6.460898	-0.22407	0.031251772	3.125177205	1.200922	0.229781	0.426725	MEVASTATIN
6.477121	-0.31579	0.03123065	3.123064983	0.692065	0.488897	0.672729	Nicarbazin
6.485721	-0.26457	0.031156924	3.115692422	0.589357	0.555622	0.727752	ENTACAPONE
6.444045	-0.23997	0.031138001	3.113800118	2.43965	0.014702	0.058852	PAPAVERINE HYDROCHLORIDE
6.469822	-0.23019	0.030967347	3.096734666	0.848811	0.395987	0.593516	DESVENLAFAXINE SUCCINATE
6.423246	-0.13806	0.030928009	3.092800937	0.585027	0.55853	0.730253	QUINAPRILAT
6.426511	-0.14545	0.030573964	3.057396394	0.838028	0.402015	0.598522	NICOTINYL ALCOHOL TARTRATE
6.426511	-0.14545	0.030438946	3.043894604	0.834327	0.404097	0.599703	AMIFOSTINE
6.382017	-0.11102	0.030100881	3.010088092	0.506314	0.612637	0.771446	ARTEMOTIL
6.428135	-0.14632	0.030097859	3.009785931	0.641887	0.520947	0.700065	SALICYLIC ACID
6.480007	-0.253	0.030050533	3.005053261	0.568429	0.569744	0.740002	CRYOFLURANE
6.469822	-0.24214	0.029839852	2.983985226	1.146666	0.25152	0.452012	LIPOAMIDE
6.498311	-0.29983	0.029788257	2.978825728	1.144683	0.25234	0.452638	NANOFIN
6.460898	-0.2128	0.029739048	2.973904751	0.634235	0.525928	0.703803	FAMOTIDINE
6.448706	-0.18962	0.029625547	2.962554677	0.56039	0.575214	0.744274	PERMETHRIN
6.447158	-0.18585	0.02962492	2.962491993	0.812015	0.416783	0.611266	METHAZOLAMIDE
6.383815	-0.05821	0.029538449	2.953844888	0.558742	0.576338	0.744837	NISOLDIPINE
6.424882	-0.2032	0.029410999	2.941099933	0.494709	0.620805	0.77756	CALCIUM CHLORIDE
6.444045	-0.23997	0.029356914	2.935691387	2.300102	0.021442	0.078523	FUSIDIC ACID
6.436163	-0.16422	0.02924057	2.924056963	0.553107	0.58019	0.748255	ASCORBYL PALMITATE
6.437751	-0.17719	0.029140334	2.914033377	1.119785	0.262805	0.463827	SULFAGUANIDINE
6.475671	-0.25398	0.028859056	2.885905622	1.108976	0.26744	0.469741	OCTOPAMINE HYDROCHLORIDE
6.444045	-0.18994	0.028816369	2.881636863	1.107336	0.268149	0.469857	NIFENAZONE
6.44248	-0.17701	0.028709848	2.87098479	0.543068	0.587083	0.752783	FOSINOPRIL SODIUM
6.409933	-0.11302	0.028670261	2.867026125	0.785848	0.431957	0.624927	TANNIC ACID
6.404834	-0.10304	0.028522217	2.852221746	0.78179	0.434338	0.626732	ESZOPICLONE
6.40654	-0.10046	0.028515999	2.851599866	0.622481	0.533626	0.710559	Manidipine
6.498311	-0.36111	0.028508186	2.85081865	0.479524	0.631566	0.78696	RILUZOLE
6.382017	-0.05274	0.02823858	2.823857968	0.602235	0.547018	0.721095	DEXPROPRANOLOL HYDROCHLORIDE [R(+)]
6.401401	-0.1491	0.027872374	2.787237374	2.183789	0.028978	0.098911	TESTOSTERONE
6.487138	-0.26744	0.027861649	2.78616489	0.527024	0.598177	0.76102	LOVASTATIN
6.482874	-0.26857	0.027520553	2.752055283	1.057541	0.290265	0.494232	PIPENZOLATE BROMIDE
6.456366	-0.27091	0.027502044	2.750204424	0.4626	0.643651	0.795821	Valnemulin HCl
6.363612	-0.07153	0.027228768	2.722876782	0.603384	0.546253	0.720889	Ferulic Acid
6.469822	-0.23019	0.027170568	2.717056783	0.744741	0.456428	0.643423	HYDROXYCHLOROQUINE SULFATE
6.399674	-0.08857	0.027023491	2.702349056	0.576321	0.564398	0.735298	STRYCHNINE SULFATE
6.451786	-0.1949	0.026943474	2.694347389	0.738517	0.4602	0.646427	MYCOPHENOLATE MOFETIL
6.487138	-0.26063	0.026911076	2.691107594	0.587447	0.556904	0.729245	Cabazitaxel
6.453318	-0.19895	0.02688022	2.688021969	0.50846	0.611131	0.770773	PHENSUCCIMIDE
6.482874	-0.2588	0.026863742	2.686374176	0.508148	0.61135	0.770773	METYRAPONE
6.403121	-0.09969	0.026431573	2.64315731	0.724486	0.468768	0.655404	QUINAPRIL HYDROCHLORIDE
6.462398	-0.22711	0.026281136	2.628113647	1.009914	0.312537	0.518814	TIOXOLONE
6.40654	-0.1025	0.026219916	2.621991561	0.559183	0.576037	0.744775	ISOCONAZOLE NITRATE
6.487138	-0.2772	0.026183574	2.61835741	1.006165	0.314336	0.520674	TRICLABENDAZOLE
6.472756	-0.24808	0.026080705	2.60807048	1.002212	0.316241	0.52214	METERGOLINE
6.436163	-0.22318	0.025988343	2.598834318	2.036176	0.041733	0.131815	BECLOMETHASONE DIPROPIONATE
6.424882	-0.2032	0.025939572	2.593957166	0.436318	0.662606	0.808904	TROXIPIDE
6.469822	-0.30008	0.02582681	2.58268102	0.572317	0.567107	0.738076	Sodium montmorillonite
6.462398	-0.21566	0.025775701	2.577570132	0.706508	0.479872	0.664892	TRIBROMOETHANOL
6.489958	-0.28292	0.025535225	2.553522499	0.98125	0.326469	0.532215	TULOBUTEROL HYDROCHLORIDE
6.463893	-0.28709	0.025426377	2.542637708	0.427686	0.66888	0.813699	EBASTINE
6.457882	-0.20683	0.025391323	2.539132282	0.695973	0.486446	0.671174	ROLIPRAM
6.44248	-0.23664	0.02528958	2.528958025	1.981428	0.047543	0.145761	LEVONORDEFRIN
6.40654	-0.10423	0.025231524	2.523152413	0.477273	0.633167	0.788336	DEBRISOQUIN SULFATE
6.436163	-0.22318	0.025196088	2.519608833	1.974103	0.04837	0.147151	CHLORAMPHENICOL
6.463893	-0.21859	0.025169078	2.516907796	0.689881	0.490269	0.673962	VALSARTAN
6.487138	-0.33708	0.025068138	2.50681383	0.42166	0.673273	0.816089	ARTESUNATE
6.44248	-0.24104	0.024765518	2.47655177	0.41657	0.676993	0.81866	BUTOPYRONOXYL
6.445604	-0.2433	0.024729766	2.472976598	1.937567	0.052676	0.155897	DYPHYLLINE
6.434569	-0.21978	0.024552602	2.455260246	1.923686	0.054394	0.159232	CLONIDINE HYDROCHLORIDE
6.39794	-0.08682	0.024547984	2.454798402	0.464344	0.642402	0.795236	ARSANILIC ACID
6.457882	-0.20668	0.024423175	2.442317547	0.520865	0.602461	0.763651	CEFAMANDOLE NAFATE
6.440909	-0.23329	0.02404319	2.404319035	1.883774	0.059596	0.169516	CLOMIPHENE CITRATE
6.463893	-0.21859	0.023966504	2.396650425	0.656919	0.511233	0.692285	DAPTOMYCIN (5 millimolar/DMSO)
6.421604	-0.19615	0.0239203	2.392030004	0.402353	0.687424	0.823364	OZAGREL HYDROCHLORIDE
6.494155	-0.35217	0.023513405	2.351340506	0.395509	0.692468	0.826337	Danofloxacin Mesylate
6.462398	-0.21566	0.022945252	2.294525153	0.628926	0.529397	0.706599	VALGANCICLOVIR HYDROCHLORIDE
6.471292	-0.30324	0.022858194	2.285819356	0.506533	0.612483	0.771442	Dalbavancin Impurity
6.421604	-0.1445	0.022748919	2.274891896	0.87418	0.38202	0.581745	NITROXOLINE
6.444045	-0.17976	0.02272604	2.272604048	0.622918	0.533339	0.710378	SIROLIMUS
6.44248	-0.24104	0.022718828	2.271882833	0.382143	0.702355	0.831983	DIMETHOATE
6.448706	-0.25464	0.02254934	2.254933954	0.499689	0.617294	0.775214	Hesperidin

6.434569	-0.16099	0.022444658	2.244465846	0.424558	0.671159	0.814877	VERAPAMIL HYDROCHLORIDE
6.409933	-0.12086	0.022366968	2.236696813	0.859503	0.390063	0.587466	TOXAPHENE
6.460898	-0.2128	0.022315733	2.231573315	0.47592	0.634131	0.788634	FLUPHENAZINE HYDROCHLORIDE
6.498311	-0.36111	0.022197029	2.219702901	0.373366	0.708876	0.8357	FROVATRIPTAN SUCCINATE
6.489958	-0.34315	0.022157449	2.215744881	0.372701	0.709371	0.835757	PEMETREXED
6.466868	-0.22491	0.022088417	2.208841669	0.471072	0.637589	0.791192	GANCICLOVIR HYDRATE
6.448706	-0.24991	0.022045321	2.204532051	1.727242	0.084124	0.217193	METHYLTHIOURACIL
6.475671	-0.24422	0.022043249	2.20432491	0.416965	0.676704	0.81866	PENICILLAMINE
6.506505	-0.31642	0.021970643	2.197064299	0.844273	0.398517	0.594697	ANISODAMINE HYDROBROMIDE
6.439333	-0.17063	0.021733095	2.173309469	0.411098	0.681001	0.820599	TOPIRAMATE
6.50515	-0.31368	0.021627311	2.162731096	0.83108	0.405929	0.60155	THREONINE (dl)
6.485721	-0.26457	0.021618316	2.161831593	0.408927	0.682593	0.821547	BUTYLATED HYDROXYTOLUENE
6.445604	-0.2433	0.021376316	2.137631552	1.674826	0.093968	0.234793	CYPROTERONE ACETATE
6.382017	-0.11113	0.021335179	2.133517879	0.472783	0.636368	0.790354	Sodium Aescinate
6.460898	-0.2128	0.021240149	2.124014946	0.452981	0.650562	0.800022	CARBENOXOLONE SODIUM
6.465383	-0.2219	0.021234913	2.123491313	0.45287	0.650643	0.800022	TOLBUTAMIDE
6.456366	-0.2036	0.021217723	2.121772332	0.452503	0.650907	0.800154	FENOFIBRATE
6.451786	-0.19038	0.021103107	2.110310727	0.460664	0.64504	0.796576	Olaparib (AZD2281, Ku-0059436)
6.489958	-0.34341	0.02093879	2.093878962	0.463999	0.642648	0.795301	Methenamine Hippurate
6.4843	-0.26169	0.020858667	2.085866703	0.394557	0.69317	0.82646	AUROTHIOGLUCOSE
6.491362	-0.34617	0.020782539	2.078253908	0.349574	0.726659	0.849516	Baclofen
6.480007	-0.253	0.020761856	2.076185572	0.392726	0.694522	0.827125	OMEPRAZOLE
6.463893	-0.21888	0.020675886	2.067588629	0.440948	0.659251	0.806908	SERATRODAST
6.466868	-0.22441	0.020382691	2.038269073	0.558687	0.576376	0.744837	ATOVAQUONE
6.457882	-0.27438	0.020371499	2.037149906	0.451428	0.651681	0.800722	Felypressin
6.439333	-0.23447	0.020236824	2.023682446	0.448444	0.653833	0.802403	Daidzin
6.460898	-0.22407	0.020178267	2.017826675	0.775397	0.438105	0.630084	COUMOPHOS
6.448706	-0.24991	0.019996413	1.999641314	1.566711	0.117182	0.274086	NYLIDRIN HYDROCHLORIDE
6.501059	-0.36702	0.019933864	1.993386382	0.335299	0.7374	0.855344	Amfenac Sodium Monohydrate
6.445604	-0.18177	0.019783953	1.978395326	0.421926	0.673079	0.816047	PIRACETAM
6.436163	-0.22746	0.019760674	1.976067426	0.332386	0.739598	0.857117	METHAMIDOPHOS
6.471292	-0.30324	0.019254821	1.925482103	0.426683	0.66961	0.813961	Rabeprazole Related Compound E
6.459392	-0.20974	0.019217627	1.921762723	0.409848	0.681918	0.821317	LUMIRACOXIB
6.503791	-0.31093	0.019164279	1.916427938	0.736432	0.461468	0.647852	ESTRADIOL PROPIONATE
6.469822	-0.29984	0.019131187	1.91311874	0.321797	0.747606	0.862493	Halobetasol Propionate
6.448706	-0.24991	0.019028329	1.902832924	1.490862	0.135998	0.301302	CINOXACIN
6.477121	-0.31579	0.018846646	1.884664645	0.417638	0.676212	0.818473	Apigenin
6.463893	-0.28732	0.018633562	1.863356247	0.412916	0.679668	0.820359	isoleucine
6.454845	-0.20052	0.018465888	1.846588821	0.393816	0.693717	0.826634	THIORIDAZINE HYDROCHLORIDE
6.450249	-0.25775	0.018457536	1.845753587	0.310466	0.756207	0.868527	DICHLORVOS
6.404834	-0.16008	0.018342015	1.834201525	0.308523	0.757684	0.869029	Proparacaine·HCl
6.459392	-0.20974	0.018260293	1.826029335	0.389431	0.696957	0.828702	TRIMETHOBENZAMIDE HYDROCHLORIDE
6.383815	-0.0553	0.018219374	1.821937413	0.397714	0.690841	0.82527	Lesinurad
6.478566	-0.31865	0.018165046	1.816504621	0.305546	0.75995	0.870458	SAXAGLIPTIN
6.462398	-0.21734	0.018138552	1.813855169	0.343104	0.73152	0.851998	BETAMETHASONE ACETATE
6.466868	-0.22035	0.018125718	1.812571766	0.39567	0.692349	0.826337	Salirasib
6.457882	-0.20668	0.018039544	1.803954401	0.384723	0.700442	0.830923	CANDESARTAN
6.472756	-0.30639	0.017998731	1.799873089	0.398848	0.690005	0.824538	Geniposidic Acid
6.447158	-0.18585	0.017948957	1.794895651	0.491978	0.622735	0.77956	LETROZOLE
6.475671	-0.24163	0.017927019	1.792701944	0.491377	0.62316	0.779902	LEVAMISOLE HYDROCHLORIDE
6.447158	-0.24661	0.01788103	1.788102985	1.400971	0.161223	0.338442	ISOXSUPRINE HYDROCHLORIDE
6.445604	-0.18281	0.017877918	1.787791776	0.490031	0.624112	0.780364	BACLOFEN HYDROCHLORIDE (+/-)
6.428135	-0.20607	0.017799384	1.77993843	1.394574	0.163144	0.341077	CLOXACILLIN SODIUM
6.453318	-0.25973	0.017749134	1.774913428	1.390637	0.164335	0.342868	METHIMAZOLE
6.49693	-0.35814	0.017730352	1.773035246	0.298234	0.765524	0.873716	Octreotide Acetate
6.453318	-0.19742	0.017717149	1.771714897	0.377848	0.705544	0.833884	CINROMIDE
6.453318	-0.26435	0.017618419	1.761841943	0.296352	0.766962	0.874553	PENCICLOVIR
6.474216	-0.23879	0.017386844	1.738684413	0.476571	0.633668	0.788619	OXCARBAZEPINE
6.454845	-0.19645	0.017373177	1.737317734	0.379242	0.704508	0.833429	Dovitinib (TKI-258) Dilactic Acid
6.394452	-0.07644	0.017291674	1.729167423	0.377463	0.705829	0.834029	Avagacestat (BMS-708163)
6.495544	-0.28446	0.017272888	1.727288806	0.32673	0.743872	0.860318	DIMETHYL FUMARATE
6.460898	-0.2143	0.017218134	1.721813439	0.325694	0.744656	0.860357	EDROPHONIUM CHLORIDE
6.475671	-0.31242	0.017207663	1.720766318	0.289442	0.772243	0.876702	CETILISAT
6.482874	-0.2588	0.017178785	1.717878527	0.32495	0.745219	0.860709	TOREMIFENE CITRATE
6.469822	-0.23019	0.017122673	1.712267335	0.46933	0.638834	0.79216	DOBUTAMINE HYDROCHLORIDE
6.414973	-0.12131	0.017045406	1.704540588	0.322427	0.747129	0.862332	FLORFENICOL
6.463893	-0.21888	0.017003185	1.700318454	0.362621	0.716888	0.841657	ADENINE
6.475671	-0.31242	0.016745504	1.674550394	0.281669	0.778198	0.879083	TIGECYCLINE
6.436163	-0.16422	0.01660231	1.660230973	0.314045	0.753487	0.867043	PREDNISOLONE HEMISUCCINATE
6.459392	-0.20978	0.016397443	1.639744294	0.449452	0.653106	0.802009	DOCUSATE SODIUM
6.456366	-0.2036	0.016345235	1.63452346	0.348589	0.727398	0.849516	TRIOXSALEN
6.434569	-0.21978	0.016309111	1.630911104	1.277812	0.201316	0.392213	HYOSCYAMINE
6.472756	-0.24808	0.016294375	1.629437484	0.626149	0.531217	0.708289	BENZONATATE
6.404834	-0.09707	0.016186398	1.618639779	0.353336	0.723837	0.847677	Lenalidomide (CC-5013)
6.481443	-0.32483	0.016128095	1.61280948	0.271284	0.786173	0.884078	ENDOSULFAN
6.466868	-0.29349	0.01607764	1.607763994	0.270435	0.786826	0.884249	METHOXYCHLOR
6.469822	-0.29984	0.016044095	1.604409452	0.269871	0.78726	0.884329	BENDIOCARB
6.471292	-0.23535	0.015905603	1.590560313	0.300867	0.763516	0.873175	BEPRIDIL HYDROCHLORIDE
6.414973	-0.18205	0.015846455	1.584645484	0.351154	0.725473	0.848816	Neomycin Sulphate B
6.437751	-0.16743	0.015780227	1.578022733	0.298495	0.765325	0.873716	DESLORATIDINE
6.509203	-0.32189	0.015662567	1.566256746	0.601871	0.54726	0.721229	MILTEFOSINE [5mM]
6.454845	-0.20052	0.015447115	1.544711494	0.329435	0.741827	0.858363	SULBACTAM
6.465383	-0.2215	0.015414165	1.541416516	0.4225	0.67266	0.815925	TENOXICAM
6.445604	-0.2433	0.015123699	1.512369916	1.184936	0.236043	0.433324	HEXACHLOROPHENE
6.463893	-0.23013	0.014923533	1.492353341	0.573471	0.566326	0.737246	NADIFLOXACIN
6.382017	-0.05457	0.014871945	1.487194466	0.281314	0.77847	0.879083	FLUOROURACIL
6.451786	-0.1949	0.014743658	1.474365838	0.404122	0.686123	0.822779	THIOTEPA
6.451786	-0.26127	0.014728606	1.472860571	0.326383	0.744135	0.860328	Lurasidone hydrochloride
6.445604	-0.2433	0.014394964	1.43949639	1.12784	0.259388	0.459538	CARBENICILLIN DISODIUM
6.502427	-0.37024	0.014156125	1.415612465	0.313697	0.753751	0.867043	Penicillin G Potassium (Benzylpenicillin)
6.447158	-0.18492	0.014117529	1.411752929	0.30108	0.763354	0.873175	SULFAMETER
6.437751	-0.16248	0.013977983	1.397798317	0.305128	0.760269	0.870628	Acitretin
6.447158	-0.2511	0.013730959	1.373095923	0.230962	0.817344	0.903122	METHYLMETHANE SULFONATE
6.487138	-0.2772	0.01370438	1.370437971	0.526623	0.598456	0.761076	BENZYL ISOTHIOCYANATE
6.460898	-0.28065	0.01363099	1.36309902	0.229281	0.818651	0.903533	PERGOLIDE MESYLATE
6.453318	-0.1979	0.013519744	1.351974414	0.370574	0.710955	0.836994	OXFENDAZOLE
6.491362	-0.28576	0.013471097	1.347109716	0.517658	0.604697	0.765261	PENTAMIDINE ISETHIONATE
6.436163	-0.22746	0.013350435	1.335043487	0.224562	0.82232	0.905436	GLIQUIDONE
6.495544	-0.35543	0.012774425	1.277442542	0.283079	0.777117	0.87833	Luteolin
6.469822	-0.23237	0.012773933	1.27739326	0.241629	0.809068	0.898044	RIMANTADINE HYDROCHLORIDE
6.488551	-0.2703	0.012706764	1.270676393	0.240358	0.810053	0.898135	ACADESINE
6.49693	-0.28727	0.012413229	1.241322943	0.234806	0.81436	0.900739	CITRIC ACID
6.440909	-0.23329	0.012332141	1.233214066	0.966218	0.333935	0.537826	MESTRANOL
6.471292	-0.23389	0.012284884	1.228488391	0.261996	0.793325	0.888607	TETRAHYDROZOLINE HYDROCHLORIDE
6.462398	-0.21584	0.011988107	1.198810727	0.255666	0.798209	0.891542	THIOGUANINE
6.468347	-0.2273	0.011845561	1.184556146	0.324685	0.745419	0.860746	ALBENDAZOLE
6.477121	-0.31579	0.011785752	1.178575173	0.26117	0.793962	0.889126	Methylcobalamin
6.453318	-0.19895	0.011656745	1.165674487	0.220496	0.825485	0.906776	ALLYLISOTHIOCYANATE
6.474216	-0.23879	0.011609357	1.160935721	0.318211	0.750325	0.8645	PHENFORMIN HYDROCHLORIDE
6.468347	-0.23915	0.011441338	1.144133833	0.43966	0.660183	0.806908	N-METHYL (-)EPHEDRINE [1R,2S]
6.503791	-0.37317	0.011360905	1.136090527	0.251755	0.80123	0.893163	Pentamidine Isethionate
6.507856	-0.31916	0.011286366	1.128636616	0.433705	0.664503	0.810833	PIMETHIXENE MALEATE
6.507856	-0.31916	0.011142232	1.114223216	0.428166	0.66853	0.813699	CARBARIL
6.447158	-0.24661	0.011078571	1.107857071	0.868001	0.385394	0.583446	DOXYLAMINE SUCCINATE
6.465383	-0.2219	0.011071944	1.107194369	0.236128	0.813334	0.90038	TRIAMTERENE
6.478566	-0.25008	0.010933275	1.093327465	0.206811	0.836157	0.912625	MEGLUMINE
6.468347	-0.23915	0.010931789	1.093178871	0.420079	0.674427	0.817101	BENFLUOREX HYDROCHLORIDE
6.471292	-0.23535	0.010653084	1.065308432	0.201511	0.840299	0.914028	AMIKACIN HYDRATE
6.511883	-0.32731	0.010476445	1.047644507	0.402582	0.687256	0.823364	PYRITHYLDIONE
6.463893	-0.21859	0.010465368	1.046536813	0.286854	0.774224	0.876824	DIXANTHOGEN
6.454845	-0.26785	0.010368322	1.036832167	0.22976	0.818278	0.903454	Demethyl
6.421604	-0.19632	0.010199729	1.019972857	0.226024	0.821183	0.904963	Puerarin (Kakonein)
6.459392	-0.20978	0.010070251	1.007025075	0.276024	0.78253	0.88142	ACRISORCIN
6.488551	-0.34038	0.009986755	0.998675508	0.221304	0.824855	0.906668	Eluxadoline
6.447158	-0.18585	0.009978949	0.997894922	0.273522	0.784452	0.882918	PERINDOPRIL ERBUMINE
6.475671	-0.24163	0.009798736	0.979873556	0.268582	0.788251	0.885054	LITHIUM CITRATE HYDRATE
6.445604	-0.18177	0.00952299	0.952298955	0.203094	0.839062	0.913071	THIAMINE HYDROCHLORIDE
6.510545	-0.3246	0.009513225	0.951322459	0.365568	0.714688	0.84023	TRIMEBUTINE MALEATE
6.444045	-0.23997	0.009451287	0.945128702	0.740504	0.458994	0.645618	HYDROXYUREA
6.468347	-0.2273	0.009361613	0.936161285	0.256601	0.797487	0.89152	DANTROLENE SODIUM
6.485721	-0.33404	0.009241816	0.924181557	0.155452	0.876465	0.932799	SYNEPHRINE TARTRATE

6.471292	-0.23306	0.009205579	0.920557903	0.252324	0.800791	0.893062	PERPHENAZINE
6.462398	-0.28388	0.009104865	0.910486486	0.153149	0.878281	0.933333	METHOMYL
6.463893	-0.21888	0.009069729	0.906972904	0.193427	0.846625	0.917183	TRIACETIN
6.451786	-0.19038	0.009030796	0.903079597	0.197135	0.843722	0.916389	Benidipine HCl
6.436163	-0.17398	0.008980455	0.898045535	0.345095	0.730023	0.851416	PRANOPROFEN
6.4133	-0.11792	0.008970978	0.897097802	0.169693	0.865252	0.926723	BENURESTAT
6.44248	-0.24104	0.008874366	0.887436569	0.149272	0.881339	0.934526	RIVAROXABAN
6.448706	-0.24991	0.008836744	0.883674444	0.692355	0.488714	0.672729	OXYQUINOLINE HEMISULFATE
6.469822	-0.23091	0.008545593	0.854559297	0.182249	0.855387	0.922795	CEFAMANDOLE SODIUM
6.463893	-0.21859	0.008378917	0.837891653	0.229665	0.818352	0.903454	CYCLOBENZAPRINE HYDROCHLORIDE
6.456366	-0.21489	0.008253321	0.825332097	0.317153	0.751127	0.865191	QUININE ETHYL CARBONATE
6.460898	-0.2128	0.008244206	0.824420606	0.175821	0.860434	0.924143	BEZAFIBRATE
6.454845	-0.20052	0.008229396	0.822939643	0.175506	0.860682	0.924143	RITODRINE HYDROCHLORIDE
6.457882	-0.26946	0.008183512	0.818351228	0.641175	0.521409	0.700319	PREDNISONE
6.487138	-0.26744	0.008102929	0.810292915	0.153273	0.878183	0.933333	AMINOREX
6.466868	-0.22639	0.007973068	0.797306755	0.150817	0.88012	0.933931	BENZBROMARONE
6.418301	-0.13781	0.007962734	0.796273351	0.305987	0.759615	0.870458	CEPHARANTHINE
6.463893	-0.22037	0.007955348	0.79553476	0.150481	0.880385	0.933955	IFOSFAMIDE
6.414973	-0.18189	0.007772011	0.777201096	0.13073	0.895989	0.942759	PROPARGITE
6.419956	-0.19277	0.007745192	0.774519159	0.171632	0.863727	0.925745	Scopolamine butylbromide
6.469822	-0.23237	0.00770624	0.770624005	0.145769	0.884103	0.935501	NEOSTIGMINE METHYLSULFATE
6.495544	-0.35516	0.007675947	0.767594707	0.129114	0.897268	0.943716	CETALKONIUM CHLORIDE
6.466868	-0.22491	0.007625981	0.762598096	0.162637	0.870804	0.930409	SULFINPYRAZONE
6.451786	-0.19431	0.007548343	0.754834349	0.160981	0.872108	0.931161	ACEBUTOLOL HYDROCHLORIDE
6.456366	-0.2036	0.007243217	0.724321675	0.154474	0.877236	0.933191	DOXOFYLLINE
6.494155	-0.35244	0.007171887	0.717188695	0.158927	0.873726	0.931817	Tauroursodeoxycholic Acid
6.454845	-0.20089	0.00710204	0.710203987	0.194666	0.845654	0.916946	OSELTAMIVIR PHOSPHATE
6.50515	-0.37582	0.007044169	0.704416941	0.118487	0.905682	0.948256	Esmolol
6.453318	-0.1979	0.006933002	0.693300162	0.190033	0.849284	0.919309	MOMETASONE FUROATE
6.485721	-0.33429	0.006891769	0.689176896	0.15272	0.878619	0.933498	CM 346(Obenoxazine)
6.454845	-0.26299	0.006891427	0.689142694	0.53994	0.589238	0.754268	SPARTEINE SULFATE
6.40654	-0.1639	0.006826061	0.682606052	0.151264	0.879767	0.933887	Valpramide
6.447158	-0.24661	0.006775369	0.677536923	0.530847	0.595525	0.758668	GUANABENZ ACETATE
6.421604	-0.1304	0.006644081	0.664408086	0.145035	0.884683	0.935863	Epothilone B (EPO906, Patupilone)
6.50515	-0.3761	0.006497447	0.649744651	0.143982	0.885515	0.936285	D-Stachyose tetrahydrate
6.482874	-0.26857	0.006344772	0.634477163	0.243813	0.807376	0.898044	PYRITINOL
6.454845	-0.21181	0.006277559	0.627755873	0.24123	0.809377	0.898135	BAMBUTEROL HYDROCHLORIDE
6.460898	-0.2128	0.006262456	0.626245578	0.133557	0.893753	0.941439	BEKANAMYCIN SULFATE
6.440909	-0.17383	0.0062246	0.622459993	0.117743	0.906271	0.948316	ALPRENOLOL HYDROCHLORIDE
6.474216	-0.23982	0.006009138	0.600913848	0.128155	0.898026	0.943739	CHOLESTEROL
6.454845	-0.26763	0.005890631	0.589063113	0.099084	0.921072	0.956564	TIOTROPIUM BROMIDE
6.511883	-0.32731	0.005791978	0.579197828	0.22257	0.82387	0.906169	GENISTEIN
6.409933	-0.12086	0.005704692	0.57046919	0.219216	0.826482	0.907526	GALLIC ACID
6.502427	-0.36996	0.00554544	0.554543957	0.093277	0.925683	0.959024	Flavoxate HCl
6.394452	-0.13789	0.005317332	0.531733239	0.117831	0.906202	0.948316	Mitiglinide calcium
6.503791	-0.37317	0.005091788	0.509178813	0.112833	0.910163	0.950058	Saikosaponin A
6.448706	-0.18806	0.00493506	0.493505987	0.105248	0.916179	0.954004	RESERPINE
6.421604	-0.1304	0.004788886	0.478888556	0.104537	0.916743	0.954182	Pacritinib (SB1518)
6.478566	-0.3189	0.004616013	0.461601321	0.10229	0.918527	0.954895	Sibutramine
6.481443	-0.32483	0.004588556	0.458855591	0.077182	0.938479	0.967582	TOSUFLOXACIN TOLUENESULFONATE HYDRATE
6.465383	-0.2215	0.004519842	0.451984205	0.123888	0.901404	0.945735	FAMCICLOVIR
6.465383	-0.2215	0.004397705	0.439770506	0.12054	0.904055	0.947352	RAMIPRIL
6.454845	-0.21181	0.004360861	0.436086072	0.167576	0.866917	0.927998	HEXETIDINE
6.454845	-0.20052	0.004336454	0.43364539	0.092482	0.926315	0.959291	SULFADIAZINE
6.454845	-0.26299	0.004314867	0.431486707	0.338068	0.735312	0.854076	L-PHENYLEPHRINE HYDROCHLORIDE
6.460898	-0.2128	0.00431113	0.431113038	0.091942	0.926744	0.959541	FENOPROFEN
6.478566	-0.2473	0.004277085	0.427708456	0.117234	0.906674	0.948402	LABETALOL HYDROCHLORIDE
6.454845	-0.26299	0.00427205	0.427204956	0.334713	0.737841	0.855662	CHLORAMPHENICOL PALMITATE
6.507856	-0.38164	0.004027579	0.402757912	0.067746	0.945988	0.972582	MOEXIPRIL HYDROCHLORIDE
6.456366	-0.20386	0.003900005	0.390000456	0.106899	0.914869	0.952834	TILMICOSIN
6.482874	-0.2588	0.003698871	0.369887067	0.069967	0.94422	0.971017	TRANEXAMIC ACID
6.468347	-0.22792	0.00369779	0.369779007	0.078862	0.937143	0.967031	NEPAFENAC
6.514548	-0.33271	0.003482341	0.348234056	0.133817	0.893547	0.941439	BRUCINE
6.480007	-0.25011	0.003464233	0.346423254	0.094954	0.924351	0.958226	TAGATOSE
6.478566	-0.2436	0.003327003	0.332700272	0.072626	0.942104	0.970342	Tivantinib (ARQ 197)
6.498311	-0.29006	0.003068106	0.306810555	0.058036	0.95372	0.976481	MINOXIDIL
6.469822	-0.30008	0.003033699	0.303369923	0.067226	0.946402	0.972617	Myclobutanil
6.514548	-0.33271	0.002999541	0.299954062	0.115264	0.908236	0.949207	ARTEMISININ
6.453318	-0.25973	0.002832097	0.283209726	0.221894	0.824397	0.906358	SALICYLANILIDE
6.49276	-0.27882	0.002817921	0.281792126	0.053303	0.95749	0.978895	ISOVALERAMIDE
6.475671	-0.31242	0.002757448	0.275744774	0.046382	0.963006	0.981342	Clorprenaline HCl
6.465383	-0.28544	0.002714646	0.271464604	0.212691	0.831568	0.909749	ETHAMBUTOL HYDROCHLORIDE
6.474216	-0.23879	0.002667479	0.266747941	0.073115	0.941714	0.970224	CEFDINIR
6.499687	-0.29285	0.002661206	0.26612062	0.050339	0.959852	0.979949	D-(+)-MALTOSE
6.477121	-0.25692	0.002502688	0.250268827	0.096172	0.923384	0.957805	CYACETACIDE
6.499687	-0.29285	0.002472672	0.247267248	0.046772	0.962695	0.981342	EPRODISATE DISODIUM
6.459392	-0.20978	0.002461241	0.246124066	0.067462	0.946214	0.972617	GLIMEPIRIDE
6.506505	-0.37901	0.002460235	0.246023528	0.054518	0.956522	0.978109	Alodan
6.460898	-0.2128	0.002334655	0.233465526	0.04979	0.960289	0.9802	FLEROXACIN
6.468347	-0.2273	0.002313848	0.231384784	0.063422	0.94943	0.974946	NILUTAMIDE
6.448706	-0.24991	0.002298385	0.229838452	0.180077	0.857092	0.923049	CEFAZOLIN SODIUM
6.480007	-0.25011	0.002218627	0.221862699	0.060812	0.951509	0.975843	CEFTIOFUR HYDROCHLORIDE
6.478566	-0.2473	0.002190949	0.219094943	0.060054	0.952113	0.975867	PROPRANOLOL HYDROCHLORIDE (+/-)
6.431364	-0.1545	0.002143812	0.214381191	0.040552	0.967653	0.983894	NICOTINE BITARTRATE
6.389166	-0.06593	0.002043832	0.204383213	0.044615	0.964414	0.982256	Veliparib (ABT-888)
6.471292	-0.23389	0.00196648	0.196648002	0.041938	0.966548	0.983536	FENSPIRIDE HYDROCHLORIDE
6.472756	-0.23686	0.001895665	0.189566539	0.040428	0.967752	0.983894	FOSFOMYCIN CALCIUM
6.383815	-0.05639	0.001706312	0.170631178	0.03639	0.970971	0.985373	VIDARABINE
6.426511	-0.20261	0.001671007	0.167100738	0.130923	0.895836	0.942759	MINOCYCLINE HYDROCHLORIDE
6.4843	-0.25499	0.001603307	0.160330732	0.034999	0.972081	0.985373	Conivaptan HCl
6.507856	-0.38164	0.001602861	0.160286112	0.026961	0.978491	0.987827	BUTENAFINE HYDROCHLORIDE
6.485721	-0.26129	0.001526213	0.152621315	0.041833	0.966632	0.983536	OXICONAZOLE NITRATE
6.457882	-0.26946	0.001513036	0.151303639	0.118546	0.905635	0.948256	CAFFEINE
6.4843	-0.26169	0.001443913	0.144391259	0.027313	0.97821	0.987827	IODOQUINOL
6.466868	-0.22441	0.00141453	0.141453013	0.038772	0.969072	0.984517	DIRITHROMYCIN
6.474216	-0.23982	0.001356934	0.135693433	0.028939	0.976913	0.986937	LIOTHYRONINE
6.474216	-0.23879	0.00120137	0.120136998	0.032929	0.973731	0.985572	HYCANTHONE
6.448706	-0.18888	0.001096347	0.109634709	0.030051	0.976027	0.98684	CLOPIDOGREL SULFATE
6.471292	-0.23535	0.000999623	0.099962312	0.018909	0.984914	0.991888	ARIPIPRAZOLE
6.503791	-0.30116	0.000972751	0.097275059	0.0184	0.98532	0.99205	NIKETHAMIDE
6.515874	-0.33539	0.000951623	0.09516231	0.036568	0.970829	0.985373	VANITIOLIDE
6.465383	-0.22339	0.000786115	0.078611483	0.01487	0.988136	0.994155	TRIENTINE HYDROCHLORIDE
6.471292	-0.23535	0.00073212	0.073211976	0.013849	0.988951	0.994525	CHLORMEZANONE
6.510545	-0.38742	0.00056146	0.056146002	0.009444	0.992465	0.995381	Salmon Calcitonin Acetate
6.482874	-0.25572	0.000499055	0.04990553	0.013679	0.989086	0.994525	MIGLITOL
6.487138	-0.33734	0.000244881	0.024488072	0.005427	0.99567	0.99732	Protocatechuic acid
6.499687	-0.28557	0.000243359	0.024335855	0.005312	0.995761	0.99732	Anisotropine Methylbromide
6.482874	-0.26857	0.000115249	0.011524861	0.004429	0.996466	0.997636	EPROBEMIDE
6.49693	-0.28009	5.67E-05	0.005667394	0.001237	0.999013	0.999599	Bazedoxifene HCl
6.399674	-0.08857	-6.32E-05	-0.006319792	-0.00135	0.998925	0.999599	MONENSIN SODIUM
6.474216	-0.24127	-0.000299168	-0.029916821	-0.00566	0.995485	0.99732	PEFLOXACINE MESYLATE
6.453318	-0.25973	-0.000431542	-0.043154173	-0.03381	0.973028	0.985572	DIMETHADIONE
6.469822	-0.23091	-0.000447313	-0.044731285	-0.00954	0.992389	0.995381	LEVOSIMENDAN
6.454845	-0.20052	-0.000472182	-0.047218188	-0.01007	0.991965	0.995381	MYCOPHENOLIC ACID
6.468347	-0.29667	-0.000934518	-0.093451785	-0.01572	0.987458	0.993669	LACTOSE MONOHYDRATE
6.468347	-0.22329	-0.000979465	-0.097946539	-0.02138	0.982942	0.990487	Everolimus (RAD001)
6.475671	-0.25398	-0.001006956	-0.100695572	-0.03869	0.969134	0.984517	LUFENURON
6.465383	-0.2215	-0.001077807	-0.107780721	-0.02954	0.976432	0.98684	PREGABALIN
6.462398	-0.21566	-0.001147071	-0.114707144	-0.03144	0.974918	0.986283	CINNARAZINE
6.468347	-0.22792	-0.001160977	-0.116097697	-0.02476	0.980247	0.988637	STAVUDINE
6.510545	-0.3246	-0.001209736	-0.120973582	-0.04649	0.962922	0.981342	HYDROQUINIDINE
6.506505	-0.31642	-0.001254381	-0.125438097	-0.0482	0.961555	0.981087	ACEDOBEN
6.472756	-0.23686	-0.001257055	-0.125705474	-0.02681	0.978612	0.987827	MEFENAMIC ACID
6.462398	-0.21566	-0.001267493	-0.126749262	-0.03474	0.972286	0.985373	VARDENAFIL HYDROCHLORIDE
6.49693	-0.35841	-0.001343821	-0.134382104	-0.02978	0.976243	0.98684	Ipragliflozin
6.478566	-0.2473	-0.001369461	-0.136946095	-0.03754	0.970057	0.985259	OXAPROZIN
6.465383	-0.2215	-0.001495099	-0.149509877	-0.04098	0.967311	0.98389	SULFLURAMID
6.480007	-0.32199	-0.001548695	-0.154869512	-0.03432	0.972623	0.985519	Azithromycin A
6.469822	-0.24214	-0.001618754	-0.161875375	-0.0622	0.9504	0.975355	NIMESULIDE
6.511883	-0.3903	-0.001783567	-0.178356704	-0.03	0.976067	0.98684	AMISULPRIDE
6.517196	-0.33807	-0.001845621	-0.184562058	-0.07092	0.94346	0.971017	AJMALINE

6.457882	-0.26946	-0.001918647	-0.191864729	-0.15033	0.880508	0.933955	ERGOCALCIFEROL
6.498311	-0.29006	-0.001924015	-0.192401451	-0.03639	0.970968	0.985373	ADRENALONE HYDROCHLORIDE
6.478566	-0.2473	-0.002055807	-0.205580712	-0.05635	0.955063	0.977203	DOXAPRAM HYDROCHLORIDE
6.477121	-0.31554	-0.002139609	-0.213960926	-0.03599	0.971291	0.985373	ESCULIN MONOHYDRATE
6.459392	-0.20978	-0.002231095	-0.223109464	-0.06115	0.951237	0.975823	ZOLPIDEM
6.40824	-0.10767	-0.002240055	-0.224005545	-0.04237	0.966202	0.983536	ETOMIDATE
6.472756	-0.23686	-0.002249699	-0.224969939	-0.04798	0.961733	0.981087	SUCCINYLSULFATHIAZOLE
6.474216	-0.23879	-0.002274671	-0.227467148	-0.06235	0.950285	0.975355	AZITHROMYCIN
6.469822	-0.23091	-0.002401291	-0.240129079	-0.05121	0.959157	0.979603	DIHYDROSTREPTOMYCIN SESQUISULFATE [5mM/10% aq DMSO]
6.459392	-0.27268	-0.002719553	-0.271955279	-0.21308	0.831268	0.909616	BENZOCAINE
6.454845	-0.26299	-0.002726304	-0.272630445	-0.2136	0.830855	0.909505	ACETYLCHOLINE CHLORIDE
6.49276	-0.27882	-0.002726677	-0.272667653	-0.05158	0.958866	0.979603	ZAPRINAST
6.472756	-0.23686	-0.002793701	-0.279370066	-0.05958	0.95249	0.975935	LEVOTHYROXINE SODIUM
6.475671	-0.24278	-0.002889419	-0.288941942	-0.06162	0.950864	0.975636	TETRACAINE HYDROCHLORIDE
6.463893	-0.21888	-0.00291955	-0.291954954	-0.06226	0.950352	0.975355	CEFEPIME HYDROCHLORIDE
6.459392	-0.20978	-0.002963087	-0.296308708	-0.08122	0.935269	0.966149	ARGININE HYDROCHLORIDE
6.462398	-0.28388	-0.003046232	-0.304623193	-0.05124	0.959135	0.979603	CARMUSTINE
6.472756	-0.23686	-0.003107435	-0.310743516	-0.06627	0.947162	0.973203	SPARFLOXACIN
6.469822	-0.23237	-0.003120567	-0.312056727	-0.05903	0.95293	0.975995	AMINOPENTAMIDE SULFATE
6.450249	-0.25319	-0.003130306	-0.313030567	-0.24526	0.806257	0.897399	HEXYLRESORCINOL
6.457882	-0.26946	-0.003137767	-0.313776684	-0.24584	0.805804	0.897285	METHENAMINE
6.454845	-0.21181	-0.003148836	-0.314883633	-0.121	0.90369	0.94722	HYDROXYTOLUIC ACID
6.451786	-0.25647	-0.003247285	-0.324728474	-0.25442	0.799168	0.892225	DOPAMINE HYDROCHLORIDE
6.394452	-0.13789	-0.003294621	-0.329462117	-0.07301	0.9418	0.970224	Menadiol Diacetate
6.424882	-0.13972	-0.003512342	-0.351234183	-0.07491	0.940289	0.969058	WARFARIN
6.477121	-0.31579	-0.003534921	-0.353492128	-0.07833	0.937563	0.967059	Proanthocyanidins
6.501059	-0.30539	-0.003542606	-0.354260619	-0.13613	0.891716	0.94067	NIALAMIDE
6.463893	-0.21859	-0.003600642	-0.360064159	-0.09869	0.921382	0.956564	TRANILAST
6.513218	-0.33002	-0.003727068	-0.372706797	-0.14322	0.886115	0.936604	MEBHYDROLIN
6.507856	-0.38164	-0.003817492	-0.381749196	-0.06421	0.948801	0.974567	CHLORDANE
6.510545	-0.3246	-0.003831449	-0.383144943	-0.14723	0.882949	0.935188	GLICLAZIDE
6.474216	-0.23982	-0.00384829	-0.384829023	-0.08207	0.93459	0.965907	FENOTEROL HYDROBROMIDE
6.459392	-0.27268	-0.003910753	-0.391075312	-0.30641	0.759296	0.870292	ETHYLENEDIAMINE TETRACETIC ACID
6.453318	-0.25973	-0.004062135	-0.406213475	-0.31827	0.750283	0.8645	CICLOPIROX OLAMINE
6.459392	-0.20978	-0.004089322	-0.408932155	-0.11209	0.910754	0.950481	CLIOQUINOL
6.454845	-0.26299	-0.004094404	-0.409440441	-0.32079	0.748366	0.862786	MAFENIDE HYDROCHLORIDE
6.448706	-0.24991	-0.004133872	-0.41338718	-0.32389	0.746023	0.861249	DOXYCYCLINE HYDROCHLORIDE
6.480007	-0.26276	-0.004142587	-0.414258724	-0.15919	0.87352	0.931817	METHOPRENE (S)
6.445604	-0.18281	-0.004153167	-0.415316704	-0.11384	0.909366	0.949807	AZTREONAM
6.466868	-0.29349	-0.004159885	-0.415988498	-0.06997	0.944216	0.971017	SORBITOL
6.450249	-0.25796	-0.004237753	-0.423775292	-0.09391	0.925182	0.9587	Fimasartan(BR-A-657)
6.437751	-0.23107	-0.004256254	-0.425625414	-0.09432	0.924857	0.958556	PAMBA
6.447158	-0.18585	-0.004315311	-0.431531084	-0.11828	0.905844	0.948256	RESORCINOL MONOACETATE
6.456366	-0.26623	-0.004453744	-0.445374415	-0.34895	0.727128	0.849516	METOPROLOL TARTRATE
6.506505	-0.31642	-0.004687554	-0.468755385	-0.18013	0.85705	0.923049	CARNITINE (dl) HYDROCHLORIDE
6.454845	-0.26763	-0.004689702	-0.468970151	-0.07888	0.937125	0.967031	PRAXADINE HYDROCHLORIDE
6.457882	-0.26946	-0.004765622	-0.476562207	-0.37338	0.708862	0.8357	PARGYLINE HYDROCHLORIDE
6.487138	-0.33734	-0.004872768	-0.487276843	-0.10798	0.914012	0.952522	2-Methoxyestradiol
6.456366	-0.27091	-0.004931432	-0.493143188	-0.08295	0.933892	0.965682	ACEPROMAZINE MALEATE
6.468347	-0.23915	-0.00496062	-0.496061987	-0.19062	0.848821	0.919002	RACECADOTRIL
6.472756	-0.23205	-0.005050469	-0.505046887	-0.11025	0.912213	0.951422	Trametinib (GSK1120212)
6.460898	-0.2143	-0.005102934	-0.510293399	-0.09653	0.923103	0.957708	ROCURONIUM BROMIDE
6.457882	-0.26946	-0.005238809	-0.523880925	-0.41046	0.68147	0.82097	PROCAINAMIDE HYDROCHLORIDE
6.459392	-0.27268	-0.005363992	-0.536399192	-0.42027	0.674291	0.817101	CHLORAMBUCIL
6.478566	-0.24865	-0.005374998	-0.537499782	-0.11463	0.908738	0.949345	ABACAVIR SULFATE
6.465383	-0.22339	-0.005415984	-0.541598445	-0.10245	0.918402	0.954895	OXOLINIC ACID
6.488551	-0.28006	-0.005633808	-0.563380838	-0.21649	0.828604	0.908254	ETHENZAMIDE
6.49693	-0.29703	-0.005648315	-0.564831517	-0.21705	0.82817	0.908254	PROPOXUR
6.466868	-0.23616	-0.005654159	-0.565415942	-0.21727	0.827995	0.908254	OXOLAMINE CITRATE
6.49276	-0.2718	-0.005757275	-0.575727491	-0.12568	0.899988	0.94498	Pimavanserin
6.447158	-0.18648	-0.005814794	-0.581479384	-0.10999	0.912416	0.95144	BISOCTRIZOLE
6.459392	-0.27268	-0.005889115	-0.588911545	-0.46141	0.644505	0.796315	CLINDAMYCIN HYDROCHLORIDE
6.465383	-0.22339	-0.005982889	-0.598288903	-0.11317	0.909895	0.95	METHSUXIMIDE
6.472756	-0.23593	-0.006008151	-0.600815052	-0.16468	0.869194	0.929464	BEMOTRIZINOL
6.506505	-0.37901	-0.006036828	-0.603682846	-0.13377	0.893581	0.941439	Micafungin
6.478566	-0.25008	-0.006125614	-0.612561395	-0.11587	0.907755	0.948952	CYCLAMIC ACID
6.489958	-0.26623	-0.006152386	-0.615238638	-0.1343	0.893164	0.941439	Pomalidomide
6.465383	-0.2174	-0.00623202	-0.623201961	-0.13604	0.89179	0.94067	Entrectinib (RXDX-101)
6.457882	-0.26946	-0.006240019	-0.624001914	-0.4889	0.62491	0.780566	NORETHINDRONE
6.471292	-0.23306	-0.006276922	-0.627692191	-0.17205	0.863399	0.925586	ATOMOXETINE HYDROCHLORIDE
6.501059	-0.29563	-0.006296617	-0.629661747	-0.11911	0.905192	0.948155	CALCIUM GLUCEPTATE
6.514548	-0.33271	-0.006316121	-0.631612092	-0.24271	0.808229	0.898044	FIPEXIDE HYDROCHLORIDE
6.460898	-0.27588	-0.00636147	-0.636147007	-0.49842	0.618189	0.775441	CAMPHOR
6.509203	-0.38453	-0.006447111	-0.644711066	-0.10844	0.913644	0.952332	EPINASTINE HYDROCHLORIDE
6.480007	-0.25157	-0.006703372	-0.670337155	-0.14296	0.886321	0.936629	DEXIBUPROFEN
6.481443	-0.25292	-0.006711395	-0.671139517	-0.18396	0.854046	0.92193	DERACOXIB
6.428135	-0.14632	-0.006865681	-0.68656811	-0.14642	0.883588	0.935375	DIAVERIDINE
6.453318	-0.20872	-0.006961301	-0.696130109	-0.2675	0.789081	0.885209	TIOPRONIN
6.463893	-0.28732	-0.007042083	-0.704208284	-0.15605	0.875993	0.932644	Isoprinosine
6.469822	-0.23019	-0.007157574	-0.715757361	-0.19619	0.844463	0.916416	CEPHALOTHIN SODIUM
6.466868	-0.22491	-0.007275288	-0.72752878	-0.15516	0.876697	0.932812	OLSALAZINE SODIUM
6.49693	-0.35841	-0.007285806	-0.728580644	-0.16145	0.871737	0.931017	Geniposide
6.460898	-0.28065	-0.007371198	-0.737119819	-0.12399	0.901325	0.945735	Triflusal
6.468347	-0.2273	-0.007420038	-0.742003822	-0.20338	0.838836	0.913071	AMITRAZ
6.511883	-0.32731	-0.007581171	-0.758117076	-0.29132	0.770803	0.876702	BERBERINE CHLORIDE
6.463893	-0.21859	-0.007611745	-0.761174498	-0.20864	0.834732	0.911847	IOPANIC ACID
6.491362	-0.26902	-0.007639381	-0.763938064	-0.16676	0.867558	0.928269	Lonafarnib
6.513218	-0.33002	-0.007760962	-0.776096193	-0.29823	0.765525	0.873716	DIPYROCETYL
6.495544	-0.35516	-0.007809902	-0.780990183	-0.13137	0.895485	0.942759	Diphemanil Methylsulfate
6.478566	-0.24865	-0.007810076	-0.781007629	-0.16656	0.867714	0.928269	ETODOLAC
6.502427	-0.36996	-0.00792467	-0.792467028	-0.1333	0.893958	0.941439	FENIPENTOL
6.456366	-0.21489	-0.008097054	-0.809705414	-0.31115	0.755688	0.868527	PIDOLIC ACID
6.503791	-0.31093	-0.008133403	-0.813340332	-0.31254	0.754626	0.867854	CYCLANDELATE
6.485721	-0.33429	-0.008162667	-0.816266741	-0.18088	0.856459	0.922979	Strontium ranelate
6.460898	-0.27588	-0.008194349	-0.819434903	-0.64202	0.520858	0.700065	METOCLOPRAMIDE HYDROCHLORIDE
6.465383	-0.2219	-0.0082504	-0.825040005	-0.17595	0.86033	0.924143	FLUMEQUINE
6.463893	-0.22037	-0.008327372	-0.8327372	-0.15752	0.874836	0.931996	EDETATE DISODIUM
6.472756	-0.30615	-0.00836005	-0.836004996	-0.14062	0.88817	0.938194	CEFSULODIN SODIUM
6.426511	-0.20688	-0.008366099	-0.836609884	-0.18539	0.852922	0.921884	Flupenthixol dihydrochloride
6.475671	-0.30736	-0.008542816	-0.854281646	-0.66933	0.503287	0.685875	PROMETHAZINE HYDROCHLORIDE
6.456366	-0.19948	-0.008557008	-0.85570078	-0.18679	0.851823	0.921279	Idasanutlin (RG-7388)
6.488551	-0.26683	-0.008663853	-0.866385312	-0.23748	0.812288	0.899809	PIPERACETAZINE
6.494155	-0.29141	-0.008679743	-0.867974264	-0.33354	0.738727	0.856495	OXYPHENONIUM BROMIDE
6.485721	-0.26129	-0.00868269	-0.868269018	-0.23799	0.811888	0.89956	DONEPEZIL HYDROCHLORIDE
6.477121	-0.31554	-0.008722017	-0.872201735	-0.14671	0.883362	0.935375	Retapamulin
6.501059	-0.29563	-0.008763864	-0.876386394	-0.16578	0.868334	0.928738	ETHION
6.513218	-0.39317	-0.008868271	-0.886827072	-0.14917	0.88142	0.934526	Clopidogrel Hydrogen Sulfate
6.462398	-0.21566	-0.008872656	-0.887265611	-0.2432	0.807852	0.898044	TIAPRIDE HYDROCHLORIDE
6.495544	-0.35543	-0.009023864	-0.902386396	-0.19997	0.841506	0.914953	Ataluren (PTC124)
6.468347	-0.23915	-0.00902852	-0.902852014	-0.34694	0.728635	0.850379	D-PHENYLALANINE
6.489958	-0.27315	-0.009161591	-0.916159073	-0.1733	0.862417	0.924921	NICORANDIL
6.475671	-0.31266	-0.009199167	-0.919916748	-0.20385	0.838469	0.913071	Foscarnet·Na (Sodium Phosphonoformate Tribasic Hexahydrate)
6.469822	-0.23091	-0.009206432	-0.920643207	-0.19634	0.844342	0.916416	TUAMINOHEPTANE SULFATE
6.503791	-0.37289	-0.009350629	-0.93506288	-0.15728	0.875022	0.931998	Fudosteine
6.460898	-0.2143	-0.009440034	-0.94400342	-0.17857	0.858279	0.923049	ERYTHROSINE SODIUM
6.466868	-0.23616	-0.009469856	-0.946985553	-0.3639	0.715932	0.84092	CHLORQUINALDOL
6.503791	-0.37317	-0.009569674	-0.956967448	-0.21206	0.832059	0.90989	Sultamicillin Tosylate
6.474216	-0.24127	-0.009575568	-0.957556821	-0.18113	0.856266	0.922979	ARSENIC TRIOXIDE DIETHANOLAMINE SALT
6.450249	-0.25775	-0.009640621	-0.964062099	-0.16216	0.871179	0.930615	Articaine HCl
6.472756	-0.24808	-0.009745233	-0.97452327	-0.37448	0.708045	0.835105	PALMIDROL [5mM]
6.453318	-0.25973	-0.009777624	-0.977762385	-0.76607	0.443633	0.635064	ISONIAZID
6.511883	-0.32731	-0.009787871	-0.978787099	-0.37612	0.706826	0.834364	CHLORAMINE-T
6.481443	-0.25449	-0.009911097	-0.991109668	-0.21137	0.832598	0.910099	OXYCLOZANIDE
6.478566	-0.3189	-0.009950496	-0.995049573	-0.2205	0.825481	0.906776	Liranaftate
6.454845	-0.21181	-0.009975771	-0.997577115	-0.38334	0.701466	0.831367	FURALTADONE
6.489958	-0.26958	-0.010066677	-1.006667704	-0.27593	0.782605	0.88142	CARPROFEN
6.481443	-0.25292	-0.01010901	-1.01090096	-0.27709	0.781714	0.880998	COLESEVALAM HYDROCHLORIDE (high mol wt copolymer @ 5mg/ml)
6.485721	-0.26129	-0.010115698	-1.011569835	-0.27727	0.781573	0.880998	ETHISTERONE
6.463893	-0.28227	-0.01015616	-1.015615999	-0.79573	0.426188	0.619558	CLIDINIUM BROMIDE
6.468347	-0.22792	-0.010167657	-1.016765728	-0.21684	0.828331	0.908254	TROPICAMIDE
6.494155	-0.29141	-0.010206215	-1.020621495	-0.3922	0.694912	0.827238	TROPISETRON HYDROCHLORIDE

6.491362	-0.28576	-0.010215241	-1.021524057	-0.39254	0.694656	0.827125	ETOSALAMIDE
6.50515	-0.30391	-0.010234422	-1.023442213	-0.19359	0.846495	0.917183	FENOLDOPAM MESYLATE
6.477121	-0.24715	-0.010314009	-1.031400867	-0.1951	0.845317	0.916946	SUCCIMER
6.515874	-0.39888	-0.010323266	-1.032326624	-0.17364	0.862146	0.924825	Tetracycline
6.469822	-0.23091	-0.010344877	-1.034487653	-0.22062	0.825387	0.906776	ACARBOSE
6.477121	-0.24572	-0.010444658	-1.044465808	-0.22275	0.82373	0.906169	ENILCONAZOLE SULFATE
6.510545	-0.38771	-0.010455595	-1.045559541	-0.23169	0.816776	0.902827	Oxytetracycline·HCl
6.499687	-0.29285	-0.010514337	-1.051433695	-0.19889	0.842351	0.915677	ELETRIPTAN HYDROBROMIDE
6.450249	-0.19119	-0.010542345	-1.054234522	-0.22483	0.822109	0.905436	ZOMEPIRAC SODIUM
6.462398	-0.21566	-0.010572473	-1.05724734	-0.28979	0.771977	0.876702	BUTOCONAZOLE
6.456366	-0.20386	-0.010614268	-1.061426823	-0.29094	0.771101	0.876702	ACYCLOVIR
6.510545	-0.38771	-0.010901074	-1.090107425	-0.24157	0.809117	0.898044	(+)-Bicuculline
6.503791	-0.30116	-0.010909058	-1.090905777	-0.20635	0.836515	0.912821	HEXYLENE GLYCOL
6.510545	-0.38742	-0.010914703	-1.091470307	-0.18359	0.854334	0.922047	Sodium Nitrite
6.477121	-0.24447	-0.011070894	-1.107089407	-0.30345	0.761546	0.871505	CEFDITORIN PIVOXIL
6.507856	-0.38192	-0.011080899	-1.108089879	-0.24555	0.80603	0.897342	Oxandrolone
6.481443	-0.26567	-0.011083173	-1.108317322	-0.4259	0.670183	0.814079	PAZUFLOXACIN MESYLATE
6.494155	-0.35244	-0.011131284	-1.113128371	-0.24667	0.805166	0.89677	Aloe-emodin
6.466868	-0.29349	-0.011215112	-1.121511187	-0.18864	0.850372	0.920098	SULFISOMIDINE
6.503791	-0.30116	-0.01123088	-1.123087998	-0.21244	0.831763	0.909769	THIAMYLAL SODIUM
6.457882	-0.26946	-0.011242367	-1.124236694	-0.88083	0.378407	0.578687	DEFERASIROX
6.465383	-0.2219	-0.011268911	-1.126891099	-0.24033	0.810076	0.898135	TRIPROLIDINE HYDROCHLORIDE
6.474216	-0.23982	-0.011287647	-1.128764703	-0.24073	0.809766	0.898135	ZIDOVUDINE [AZT]
6.474216	-0.23879	-0.011317584	-1.131758404	-0.31021	0.756399	0.868527	VENLAFAXINE HYDROCHLORIDE
6.472756	-0.30615	-0.011358187	-1.135818696	-0.19105	0.848486	0.918834	PYRIDOSTIGMINE BROMIDE
6.485721	-0.33429	-0.011450095	-1.145009467	-0.25373	0.799703	0.892401	Swertiamarin
6.487138	-0.26744	-0.011472802	-1.147280246	-0.21702	0.828195	0.908254	BUTYL PARABEN
6.507856	-0.38164	-0.011487473	-1.148747294	-0.19323	0.846782	0.917183	Cilastatin·Na
6.509203	-0.38482	-0.011548727	-1.154872724	-0.25592	0.798015	0.89152	Pikamilone
6.41162	-0.17468	-0.011572076	-1.157207564	-0.19465	0.845668	0.916946	LORGLUMIDE SODIUM
6.448706	-0.24991	-0.011745489	-1.174548875	-0.92025	0.35744	0.557955	CYCLOPHOSPHAMIDE
6.456366	-0.2036	-0.011816761	-1.181676098	-0.25201	0.801032	0.893136	CARBIDOPA
6.487138	-0.2772	-0.011911815	-1.191181543	-0.45774	0.64714	0.797867	MECYSTEINE HYDROCHLORIDE
6.514548	-0.33271	-0.011938407	-1.193840669	-0.45876	0.646406	0.797358	FLOPROPIONE
6.466868	-0.2886	-0.012276834	-1.227683372	-0.96188	0.336107	0.539458	MEPENZOLATE BROMIDE
6.465383	-0.2215	-0.012371512	-1.237151179	-0.3391	0.734533	0.853931	LEVOCETIRIZINE DIHYDROCHLORIDE
6.457882	-0.26946	-0.012400956	-1.240095601	-0.97161	0.331245	0.53634	DICYCLOMINE HYDROCHLORIDE
6.421604	-0.19615	-0.012541223	-1.254122303	-0.21095	0.832926	0.910263	FLUROTHYL
6.501059	-0.30539	-0.012550666	-1.255066631	-0.48229	0.629601	0.785467	FASUDIL HYDROCHLORIDE
6.502427	-0.37024	-0.012607224	-1.260722407	-0.27937	0.779958	0.879794	Ecabet Sodium
6.480007	-0.26276	-0.012692727	-1.269272715	-0.48775	0.625729	0.781398	CIPROFIBRATE
6.481443	-0.25292	-0.012720076	-1.272007617	-0.34866	0.727348	0.849516	CEFTIBUTEN
6.506505	-0.37873	-0.012722267	-1.272226746	-0.214	0.83055	0.909414	Pamidronate Disodium Pentahydrate (Pamidronic Acid)
6.471292	-0.23389	-0.012812131	-1.281213091	-0.27324	0.784669	0.882968	AMLEXANOX
6.488551	-0.34038	-0.012931873	-1.293187296	-0.28657	0.774443	0.876858	Arbidol hydrochloride
6.498311	-0.29006	-0.013089254	-1.308925415	-0.24759	0.804449	0.896185	ESTRAMUSTINE
6.439333	-0.1804	-0.013166523	-1.316652252	-0.50595	0.612889	0.771574	PIPEMIDIC ACID
6.465383	-0.2215	-0.013238638	-1.323863819	-0.36287	0.716703	0.841632	ETHYLNOREPINEPHRINE HYDROCHLORIDE
6.436163	-0.15933	-0.013352557	-1.335255656	-0.29148	0.770688	0.876702	Cediranib (AZD2171)
6.445604	-0.18177	-0.013395747	-1.339574665	-0.28569	0.775118	0.877234	RACEPHEDRINE HYDROCHLORIDE
6.44248	-0.24124	-0.013429049	-1.34290489	-0.29758	0.76602	0.873987	10,11-Dihydrocarbamazepine
6.510545	-0.3246	-0.013432898	-1.343289819	-0.51619	0.605721	0.765758	MILRINONE
6.454845	-0.20205	-0.013537839	-1.353783869	-0.25608	0.79789	0.89152	LIOTHYRONINE (L- isomer) SODIUM
6.471292	-0.23306	-0.013541919	-1.354191909	-0.37118	0.710502	0.836654	SERTRALINE HYDROCHLORIDE
6.471292	-0.23306	-0.013561328	-1.356132766	-0.37171	0.710106	0.836379	ALENDRONATE SODIUM TRIHYDRATE
6.465383	-0.2219	-0.013574295	-1.357429477	-0.28949	0.772203	0.876702	ETHOSUXIMIDE
6.466868	-0.23616	-0.013672327	-1.367232663	-0.52539	0.599311	0.761597	METICRANE
6.445604	-0.1931	-0.013711598	-1.371159848	-0.5269	0.598263	0.76102	BROXALDINE
6.477121	-0.24572	-0.013748208	-1.374820795	-0.2932	0.769367	0.87653	CHENODIOL
6.4843	-0.25851	-0.013817162	-1.381716152	-0.37873	0.704891	0.833497	CLARITHROMYCIN
6.4843	-0.26169	-0.013888208	-1.388820825	-0.26271	0.792777	0.888382	ALISKIREN HEMIFUMARATE
6.491362	-0.34643	-0.013954464	-1.395446415	-0.30923	0.757148	0.868803	Tanshinone IIA-sulfonic sodium
6.462398	-0.21734	-0.014057003	-1.405700305	-0.2659	0.790317	0.886208	GEMIFLOXACIN MESYLATE
6.475671	-0.24163	-0.01407874	-1.407874016	-0.3859	0.699573	0.830277	ROXITHROMYCIN
6.437751	-0.16744	-0.014086688	-1.408668785	-0.38611	0.699412	0.830277	CHOLINE CHLORIDE
6.437751	-0.16744	-0.014101243	-1.410124259	-0.38651	0.699117	0.830277	METHYSERGIDE MALEATE
6.477121	-0.24715	-0.014168708	-1.416870826	-0.26801	0.78869	0.885159	BROMINDIONE
6.515874	-0.39888	-0.014271057	-1.427105664	-0.24005	0.810294	0.898182	RAFOXANIDE
6.501059	-0.29563	-0.014281396	-1.428139645	-0.27014	0.78705	0.884287	LEVOSULPIRIDE
6.457882	-0.2082	-0.014393825	-1.439382495	-0.27227	0.785414	0.883419	MITOTANE
6.40654	-0.1639	-0.014490932	-1.449093172	-0.32112	0.748123	0.862786	Imperatorin
6.468347	-0.22792	-0.014582093	-1.458209255	-0.31099	0.75581	0.868527	SELENOMETHIONINE
6.466868	-0.22491	-0.014718263	-1.471826302	-0.31389	0.753604	0.867043	BENZOYL PEROXIDE
6.462398	-0.27908	-0.014849566	-1.484956582	-1.16346	0.244644	0.443076	PIPERAZINE
6.462398	-0.27908	-0.01485235	-1.48523496	-1.16368	0.244556	0.443076	HALAZONE
6.462398	-0.21566	-0.014879867	-1.487986673	-0.40786	0.68338	0.821663	DIMPYLATE
6.495544	-0.29423	-0.014886319	-1.488631861	-0.57204	0.567294	0.738131	TODRALAZINE HYDROCHLORIDE
6.482874	-0.2588	-0.014908137	-1.490813738	-0.282	0.777945	0.879072	GENTAMICIN SULFATE
6.477121	-0.24447	-0.015024025	-1.502402532	-0.41181	0.680481	0.820359	MELOXICAM
6.471292	-0.23306	-0.015029338	-1.502933829	-0.41195	0.680375	0.820359	QUINETHAZONE
6.460898	-0.27588	-0.015089635	-1.50896355	-1.18227	0.2371	0.434415	LINCOMYCIN HYDROCHLORIDE
6.485721	-0.27434	-0.015171859	-1.517185936	-0.58301	0.559884	0.731367	PIROMIDIC ACID
6.471292	-0.23306	-0.015274341	-1.527434126	-0.41867	0.675459	0.817964	ACETRIAZOIC ACID
6.485721	-0.25781	-0.015383496	-1.538349578	-0.33581	0.737015	0.855091	Clevudine
6.453318	-0.19895	-0.015384497	-1.538449675	-0.29101	0.771044	0.876702	TEMAZEPAM
6.474216	-0.23495	-0.015579832	-1.557983242	-0.3401	0.733785	0.853665	Fludarabine
6.439333	-0.17054	-0.015584311	-1.558431114	-0.42716	0.66926	0.813922	ESTROPIPATE
6.478566	-0.25008	-0.015680553	-1.568055336	-0.29661	0.766765	0.874546	DILTIAZEM HYDROCHLORIDE
6.501059	-0.30539	-0.015744611	-1.574461094	-0.60502	0.545164	0.720133	CLOPERASTINE HYDROCHLORIDE
6.462398	-0.27908	-0.015747283	-1.574728286	-1.23379	0.21728	0.412792	COTININE
6.460898	-0.20848	-0.015793838	-1.579383807	-0.34477	0.73027	0.85151	Fidaxomicin
6.457882	-0.20668	-0.015907958	-1.590795765	-0.33926	0.734411	0.853931	MIZORIBINE
6.457882	-0.26946	-0.015959418	-1.59594178	-1.25041	0.211148	0.405455	ERYTHROMYCIN ETHYLSUCCINATE
6.463893	-0.28227	-0.015998699	-1.599869891	-1.25349	0.210027	0.403504	ANTIPYRINE
6.511883	-0.31755	-0.01608318	-1.608317983	-0.30423	0.760956	0.87122	AMMONIUM LACTATE
6.511883	-0.3903	-0.01609277	-1.609277042	-0.27069	0.78663	0.884249	Nafarelin Acetate
6.460898	-0.20848	-0.016220967	-1.622096711	-0.35409	0.723271	0.847402	Tolterodine tartrate
6.488551	-0.2703	-0.016249822	-1.62498224	-0.30738	0.758556	0.869639	OCTISALATE
6.482874	-0.25572	-0.016524379	-1.652437902	-0.45293	0.650598	0.800022	METAXALONE
6.514548	-0.33271	-0.016636172	-1.663617235	-0.63928	0.522638	0.701234	CLEMIZOLE HYDROCHLORIDE
6.510545	-0.3246	-0.016644934	-1.664493357	-0.63962	0.52242	0.701191	BECLAMIDE
6.459392	-0.27268	-0.0166657	-1.666570047	-1.30575	0.191637	0.380156	GEMFIBROZIL
6.480007	-0.25011	-0.016673077	-1.667307741	-0.45701	0.647666	0.798087	HALCINONIDE
6.502427	-0.30816	-0.01682485	-1.682485033	-0.64653	0.517934	0.697297	CITIOLONE
6.489958	-0.34315	-0.01689039	-1.689039029	-0.28411	0.776329	0.877828	CHLOROACETOXYQUINOLINE
6.506505	-0.30666	-0.017018412	-1.701841205	-0.32192	0.747516	0.862493	NADOLOL
6.419956	-0.12712	-0.017084183	-1.708418284	-0.37293	0.709198	0.835757	Felbamate
6.507856	-0.38192	-0.017186586	-1.718658574	-0.38085	0.703314	0.832209	Scopoletin
6.369216	-0.02629	-0.017252179	-1.725217899	-0.3766	0.70647	0.834364	Afatinib (BIBW2992)
6.485721	-0.26129	-0.017503421	-1.750342108	-0.47977	0.631394	0.786936	MEPHENTERMINE SULFATE
6.472756	-0.30639	-0.017643748	-1.764374775	-0.39098	0.695811	0.828115	Talc
6.495544	-0.35543	-0.017759228	-1.775922811	-0.39354	0.69392	0.826634	Zinc bacitracin
6.510545	-0.31484	-0.017834013	-1.783401289	-0.33734	0.735858	0.854135	ESTRIOL TRIPROPIONATE
6.50515	-0.3761	-0.018110786	-1.811078627	-0.40133	0.688176	0.823376	Mafenide·HCl
6.514548	-0.33271	-0.018124237	-1.812423672	-0.69647	0.486137	0.671174	EPIESTRIOL
6.50515	-0.30391	-0.018157444	-1.81574439	-0.34346	0.731251	0.85199	DARIFENACIN HYDROBROMIDE
6.4843	-0.25851	-0.018328428	-1.832842831	-0.50238	0.6154	0.773405	ENALAPRILAT
6.509203	-0.38482	-0.018379876	-1.837987641	-0.40729	0.683792	0.821796	Levocetirizine Dihydrochloride
6.4843	-0.26029	-0.018493021	-1.849302101	-0.39439	0.69329	0.82646	ALTHIAZIDE
6.488551	-0.28006	-0.018521159	-1.852115929	-0.71172	0.476639	0.662385	ACEXAMIC ACID
6.465383	-0.28544	-0.018535717	-1.853571742	-1.45227	0.146428	0.317002	AMPICILLIN SODIUM
6.487138	-0.33734	-0.018587629	-1.858762947	-0.4119	0.680414	0.820359	Amfepramone hydrochloride
6.478566	-0.24865	-0.018735928	-1.873592802	-0.39957	0.68947	0.824209	NEFAZODONE HYDROCHLORIDE
6.498311	-0.36138	-0.018803836	-1.880383618	-0.41669	0.676906	0.81866	Isavuconazole
6.482874	-0.25739	-0.018810963	-1.881096331	-0.40118	0.688291	0.823376	SULFAMETHOXAZOLE
6.4843	-0.26029	-0.01902017	-1.902016965	-0.40564	0.68501	0.8224	THIABENDAZOLE
6.471292	-0.24512	-0.01902362	-1.902361991	-0.73103	0.464763	0.651049	TROXERUTIN
6.456366	-0.27091	-0.019080184	-1.908018392	-0.32094	0.748256	0.862786	CLORSULON
6.506505	-0.31642	-0.019238824	-1.923882427	-0.7393	0.459727	0.646116	AMEZINIUM METILSULFATE
6.506505	-0.31642	-0.019243596	-1.924359617	-0.73948	0.459616	0.646116	SODIUM TETRADECYL SULFATE
6.456366	-0.26623	-0.019305541	-1.930554074	-1.51258	0.130386	0.294736	CROMOLYN SODIUM

6.485721	-0.33429	-0.019307401	-1.930740112	-0.42785	0.668762	0.813699	Topiroxostat
6.444045	-0.24441	-0.019397339	-1.939733886	-0.32627	0.744217	0.860328	SENNOSIDE A
6.463893	-0.28227	-0.019411282	-1.94112824	-1.52087	0.128293	0.290905	CEFOTAXIME SODIUM
6.50515	-0.3761	-0.01943638	-1.943638021	-0.43071	0.666682	0.812137	Limonin
6.487138	-0.26407	-0.019668475	-1.966847545	-0.53911	0.589811	0.754268	AVOBENZONE
6.474216	-0.25104	-0.019805978	-1.980597799	-0.76109	0.446603	0.637559	PICONOL
6.482874	-0.25739	-0.019883462	-1.988346183	-0.42405	0.671531	0.814942	FENCLONINE (+/-)
6.510545	-0.31484	-0.01993916	-1.993916029	-0.37716	0.706052	0.834099	PHENOBARBITAL
6.416641	-0.18564	-0.019978546	-1.997854563	-0.44272	0.657968	0.805739	Chrysophanic Acid
6.475671	-0.31266	-0.020014135	-2.001413516	-0.44351	0.657398	0.805739	Flunisolide
6.491362	-0.27233	-0.020080974	-2.00809738	-0.55042	0.582034	0.74906	THONZONIUM BROMIDE
6.466868	-0.2886	-0.020189313	-2.018931288	-1.58182	0.11369	0.268836	CARBACHOL
6.471292	-0.23389	-0.020205672	-2.020567201	-0.43092	0.666527	0.812137	RONIDAZOLE
6.495544	-0.35516	-0.020328122	-2.032812192	-0.34193	0.732403	0.852679	GLYCYRRHIZIN
6.459392	-0.20978	-0.020370173	-2.037017301	-0.55834	0.57661	0.744951	NIMODIPINE
6.445604	-0.24796	-0.020383213	-2.038321347	-0.45169	0.651494	0.800684	Arbutin
6.472756	-0.23686	-0.020471366	-2.047136625	-0.43659	0.662412	0.80886	CAPSAICIN
6.474216	-0.30426	-0.020530301	-2.053030092	-1.60854	0.107717	0.257442	ETHACRYNIC ACID
6.491362	-0.27233	-0.020637737	-2.063773739	-0.56568	0.571613	0.741677	CANRENONE
6.487138	-0.26605	-0.020643155	-2.064315464	-0.44025	0.659756	0.806908	FLUTAMIDE
6.503791	-0.37317	-0.020654038	-2.065403814	-0.45769	0.647176	0.797867	Leucovorin Calcium Pentahydrate
6.515874	-0.33539	-0.020698829	-2.069882859	-0.7954	0.426381	0.619662	SECNIDAZOLE
6.450249	-0.25319	-0.020830099	-2.08300993	-1.63203	0.102673	0.249382	OXYMETAZOLINE HYDROCHLORIDE
6.448706	-0.24991	-0.020936954	-2.093695358	-1.6404	0.100922	0.246762	DEHYDROCHOLATE SODIUM
6.472756	-0.23686	-0.02110273	-2.110272956	-0.45005	0.652674	0.801749	ADAPALENE
6.454845	-0.19645	-0.021136467	-2.113646671	-0.46139	0.644517	0.796315	Apatinib
6.468347	-0.29176	-0.02122361	-2.122361013	-1.66286	0.09634	0.238868	PRAZIQUANTEL
6.501059	-0.30539	-0.021450638	-2.145063759	-0.82429	0.409774	0.60559	DROFENINE HYDROCHLORIDE
6.499687	-0.29285	-0.021500753	-2.150075265	-0.4067	0.684226	0.821796	MALATHION
6.453318	-0.25973	-0.021528285	-2.152828544	-1.68673	0.091655	0.230941	DIBUCAINE HYDROCHLORIDE
6.4843	-0.33098	-0.021718053	-2.171805266	-0.36531	0.71488	0.840263	PIRIBEDIL HYDROCHLORIDE
6.466868	-0.2886	-0.02171964	-2.171963982	-1.70172	0.088807	0.225653	NOVOBIOCIN SODIUM
6.495544	-0.35543	-0.021723665	-2.172366465	-0.48139	0.630238	0.785908	Etofibrate
6.469822	-0.23091	-0.021741014	-2.174101389	-0.46366	0.642889	0.795301	RESORCINOL
6.503791	-0.29372	-0.021798895	-2.179889497	-0.47585	0.63418	0.788634	Brexpiprazole
6.482874	-0.25739	-0.021819201	-2.181920142	-0.46533	0.641695	0.794555	CEFMETAZOLE SODIUM
6.472756	-0.23832	-0.02186529	-2.186529042	-0.4136	0.679168	0.820359	DESOXYMETASONE
6.488551	-0.26683	-0.022175251	-2.217525073	-0.60782	0.543307	0.718496	IMIQUIMOD HYDROCHLORIDE
6.517196	-0.40172	-0.022277602	-2.227760194	-0.37472	0.707868	0.835088	IPROHEPTINE HYDROCHLORIDE
6.469822	-0.23091	-0.022329954	-2.232995444	-0.47622	0.633915	0.788634	alpha-TOCHOPHERYL ACETATE [4mM]
6.513218	-0.33002	-0.022331357	-2.233135734	-0.85813	0.390818	0.587882	AMINOTHIAZOLE
6.485721	-0.26129	-0.022393501	-2.239350066	-0.6138	0.539346	0.714291	TRIFLURIDINE
6.522444	-0.3487	-0.022657821	-2.265782077	-0.87068	0.383929	0.582262	PRIDINOL METHANESULFONATE
6.466868	-0.2886	-0.022720576	-2.272057624	-1.78015	0.075052	0.200451	MECLIZINE HYDROCHLORIDE
6.418301	-0.18921	-0.022754931	-2.275493135	-0.50424	0.61409	0.77264	5-Acetylsalicylic acid
6.468347	-0.29176	-0.022796306	-2.279630574	-1.78608	0.074086	0.198493	COLISTIMETHATE SODIUM
6.526339	-0.35659	-0.022872344	-2.287234384	-0.87892	0.379443	0.57924	AMBROXOL HYDROCHLORIDE
6.515874	-0.39888	-0.022913913	-2.291391282	-0.38542	0.699923	0.830499	Terazosin·HCl
6.50515	-0.30391	-0.022950887	-2.295088664	-0.43413	0.664192	0.810646	MOLINDONE HYDROCHLORIDE
6.481443	-0.25449	-0.022955399	-2.295539893	-0.48956	0.624444	0.780364	DEHYDROCHOLIC ACID
6.491362	-0.27233	-0.022970937	-2.297093679	-0.62963	0.528937	0.706528	RIFAXIMIN
6.501059	-0.36702	-0.022994853	-2.299485269	-0.38679	0.698915	0.830265	Bivalirudin Trifluoroacetate
6.485721	-0.26317	-0.023007709	-2.3007709	-0.49068	0.623655	0.78033	ROXATIDINE ACETATE HYDROCHLORIDE
6.50515	-0.37582	-0.023148823	-2.314882316	-0.38938	0.696998	0.828702	Sodium ascorbate
6.487138	-0.26407	-0.023205314	-2.320531426	-0.63605	0.524741	0.702804	AMINOLEVULINIC ACID HYDROCHLORIDE
6.489958	-0.27315	-0.023246284	-2.324628395	-0.43972	0.660139	0.806908	TICARCILLIN DISODIUM
6.480007	-0.25011	-0.023251062	-2.325106164	-0.63731	0.523924	0.702223	MORANTEL CITRATE
6.488551	-0.26891	-0.023393896	-2.339389646	-0.49891	0.61784	0.775441	THEOPHYLLINE
6.444045	-0.18018	-0.023406151	-2.340615117	-0.44275	0.65795	0.805739	VORTIOXETINE HYDROBROMIDE
6.475671	-0.24278	-0.023629644	-2.362964448	-0.50394	0.614303	0.77264	PALIPERIDONE
6.499687	-0.36434	-0.023648298	-2.364829767	-0.52404	0.60025	0.762086	Torezolid
6.459392	-0.20978	-0.023749667	-2.37496675	-0.65098	0.515063	0.694817	FLOXURIDINE
6.523746	-0.35134	-0.023760455	-2.376045454	-0.91305	0.361216	0.562564	CHLORINDIONE
6.475671	-0.25398	-0.023760457	-2.376045723	-0.91305	0.361216	0.562564	CHLOROBUTANOL
6.487138	-0.2772	-0.02377952	-2.377952029	-0.91378	0.360831	0.562391	DOCOSANOL
6.480007	-0.25011	-0.023869782	-2.386978211	-0.65427	0.51294	0.693491	TRIMIPRAMINE MALEATE
6.491362	-0.27233	-0.023990064	-2.399006427	-0.65756	0.510818	0.692085	PHYSOSTIGMINE SALICYLATE
6.49693	-0.35814	-0.024020057	-2.402005745	-0.40403	0.68619	0.822779	4-NAPHTHALIMIDOBUTYRIC ACID
6.424882	-0.20337	-0.024072172	-2.407217204	-0.53343	0.593733	0.75695	Aristolochic Acid
6.460898	-0.27588	-0.024082816	-2.408281633	-1.88688	0.059177	0.169011	METRONIDAZOLE
6.489958	-0.26958	-0.024111575	-2.411157522	-0.66089	0.50868	0.690284	SEVOFLURANE
6.474216	-0.30929	-0.024303265	-2.430326486	-0.40879	0.682691	0.821547	EQUILIN
6.474216	-0.23982	-0.024348504	-2.434850353	-0.51927	0.603571	0.764596	ADENOSINE 5-MONOPHOSPHATE
6.491362	-0.27233	-0.024379269	-2.437926869	-0.66823	0.503985	0.686603	CEFPROZIL
6.477121	-0.24572	-0.024415945	-2.441594502	-0.52071	0.602568	0.763651	SPECTINOMYCIN HYDROCHLORIDE
6.418301	-0.12383	-0.024416358	-2.441635757	-0.53299	0.594041	0.756966	Axitinib
6.514548	-0.39603	-0.024486662	-2.448666223	-0.41188	0.680428	0.820359	ACETOHEXAMIDE
6.495544	-0.28446	-0.02449827	-2.449827004	-0.4634	0.643075	0.795301	METHYLATROPINE NITRATE
6.510545	-0.38742	-0.024498393	-2.449839258	-0.41208	0.680283	0.820359	CURCUMIN
6.477121	-0.31045	-0.02453435	-2.453434974	-1.92226	0.054574	0.159575	CHLORPROPAMIDE
6.518514	-0.40486	-0.024655394	-2.46553936	-0.54636	0.58482	0.751198	Tetrahydropalmatine hydrochloride
6.460898	-0.27588	-0.024667624	-2.466762431	-1.9327	0.053273	0.15721	PHENYL AMINOSALICYLATE
6.4843	-0.26169	-0.024768695	-2.476869508	-0.46852	0.639414	0.792495	TROMETHAMINE
6.514548	-0.32294	-0.024825848	-2.482584834	-0.4696	0.638641	0.792113	VIOMYCIN SULFATE
6.472756	-0.23686	-0.024851934	-2.485193419	-0.53001	0.596106	0.759031	NALIDIXIC ACID
6.445604	-0.17809	-0.024979651	-2.49796507	-0.54529	0.585557	0.751957	Mubritinib (TAK 165)
6.495544	-0.29423	-0.024990245	-2.499024501	-0.96031	0.3369	0.54015	TENATOPRAZOLE
6.485721	-0.27434	-0.024995269	-2.49952692	-0.9605	0.336803	0.54015	BERGAPTEN
6.488551	-0.26891	-0.025050983	-2.505098276	-0.53425	0.593166	0.756793	PIPERINE
6.468347	-0.29176	-0.025207454	-2.520745405	-1.97499	0.048269	0.147018	PIROXICAM
6.474216	-0.23982	-0.025275293	-2.527529266	-0.53904	0.589861	0.754268	MECLOCYCLINE SULFOSALICYLATE
6.511883	-0.3903	-0.025330043	-2.533004253	-0.42607	0.67006	0.814079	Imipenem
6.487138	-0.33734	-0.025353036	-2.535303559	-0.56182	0.57424	0.743767	Hydroferulic acid
6.495544	-0.28051	-0.025363681	-2.536368061	-0.69521	0.486921	0.671434	CYCLOTHIAZIDE
6.434569	-0.22422	-0.025382499	-2.538249942	-0.56247	0.573795	0.743379	(-)-Huperzine A (HupA)
6.521138	-0.4105	-0.025413828	-2.54138278	-0.56317	0.573323	0.742954	Cefminox Sodium
6.503791	-0.31093	-0.025432977	-2.543297738	-0.97732	0.32841	0.533848	AMINOPYRINE
6.481443	-0.25449	-0.025434494	-2.543449448	-0.54243	0.58752	0.753156	ANTAZOLINE
6.463893	-0.28227	-0.025441015	-2.544101531	-1.99329	0.046229	0.142673	PENICILLIN G POTASSIUM
6.468347	-0.29667	-0.025470313	-2.547031308	-0.42842	0.668342	0.813699	Trientine Dihydrochloride
6.485721	-0.26317	-0.025548221	-2.554822142	-0.54486	0.585851	0.752146	AMOROLFINE HYDROCHLORIDE
6.507856	-0.31916	-0.025588056	-2.558805555	-0.98328	0.325469	0.531906	METHYL PARATHIONE
6.495544	-0.35516	-0.025656459	-2.56564594	-0.43156	0.666064	0.811964	Alitretinoin
6.523746	-0.41581	-0.025717814	-2.571781357	-0.43259	0.665314	0.811629	LYNESTRENOL
6.521138	-0.34605	-0.0261382	-2.613819968	-1.00442	0.315176	0.521389	ACTARIT
6.501059	-0.29563	-0.026209935	-2.6209935	-0.49578	0.620049	0.777148	DIFLORASONE DIACETATE
6.4843	-0.26169	-0.026355538	-2.635553809	-0.49853	0.618107	0.775441	OCTOCRYLENE
6.52763	-0.3592	-0.02659193	-2.659193026	-1.02186	0.306849	0.511082	CINCHOPHEN
6.474216	-0.24127	-0.026638574	-2.663857381	-0.50389	0.61434	0.77264	HALOTHANE
6.501059	-0.3673	-0.02666511	-2.666510983	-0.59089	0.554592	0.727148	Indinavir
6.515874	-0.39888	-0.026712731	-2.671273086	-0.44932	0.653199	0.802009	4-Aminosalicylic Acid
6.518514	-0.40486	-0.026813863	-2.681386311	-0.59419	0.552385	0.725732	Catharanthine
6.471292	-0.22914	-0.026871583	-2.687158331	-0.58658	0.557483	0.729435	Enzastaurin (LY317615)
6.513218	-0.33002	-0.026899729	-2.689972889	-1.03368	0.301284	0.505379	IRSOGLADINE MALEATE
6.40824	-0.16756	-0.026923276	-2.692327557	-0.59661	0.550765	0.724542	Biperiden·HCl
6.517196	-0.33807	-0.026990701	-2.699070113	-1.03718	0.299652	0.50405	EFLOXATE
6.466868	-0.2886	-0.027055735	-2.705573518	-2.11981	0.034022	0.112022	PHENYTOIN SODIUM
6.436163	-0.15933	-0.027210741	-2.721074056	-0.59399	0.55252	0.725732	Temsirolimus (CCI-779, NSC 683864)
6.502427	-0.30816	-0.027255714	-2.725571358	-1.04736	0.294932	0.499686	NONIVAMIDE
6.480007	-0.25157	-0.027292479	-2.729247876	-0.58206	0.560528	0.731558	SULFISOXAZOLE
6.49276	-0.27507	-0.027347022	-2.734702242	-0.74958	0.453509	0.641781	LOSARTAN
6.472756	-0.30115	-0.027359658	-2.735965766	-2.14362	0.032064	0.107049	CLEMASTINE FUMARATE
6.482874	-0.32816	-0.027412385	-2.741238471	-0.60745	0.543551	0.718559	Formoterol
6.49693	-0.28322	-0.027552506	-2.755250592	-0.75521	0.450123	0.639287	HYGROMYCIN B
6.465383	-0.28544	-0.027558021	-2.755802111	-2.15916	0.030838	0.103943	AMPHOTERICIN B
6.471292	-0.23306	-0.027579527	-2.757952702	-0.75595	0.449679	0.638835	CLOSANTEL
6.462398	-0.21734	-0.027608372	-2.760837186	-0.52223	0.601508	0.762685	DIGITONIN
6.502427	-0.36996	-0.027664279	-2.766427868	-0.46533	0.641696	0.794555	PENTOBARBITAL
6.472756	-0.23593	-0.027716462	-2.771646181	-0.7597	0.447431	0.638119	DESLORATADINE HYDROCHLORIDE
6.478566	-0.2473	-0.027785735	-2.778573524	-0.7616	0.446297	0.637389	BENDROFLUMETHIAZIDE
6.513218	-0.32025	-0.027811743	-2.781174303	-0.52608	0.598833	0.761177	TEMOZOLOMIDE

6.471292	-0.23306	-0.027854663	-2.78546634	-0.76349	0.44517	0.63649	THIOSTREPTON
6.480007	-0.25157	-0.027894992	-2.789499156	-0.59491	0.551906	0.725669	AZATADINE MALEATE
6.511883	-0.3903	-0.02804386	-2.804386009	-0.47171	0.637131	0.790815	MOLSIDOMINE
6.511883	-0.31755	-0.028051404	-2.805140366	-0.53061	0.595687	0.758685	NITHIAMIDE
6.469822	-0.2949	-0.028076202	-2.807620235	-2.19976	0.027824	0.095997	ACETAMINOPHEN
6.450249	-0.25775	-0.028076943	-2.807694283	-0.47227	0.636734	0.790514	GHRP-2
6.494155	-0.27779	-0.028125559	-2.812555893	-0.77092	0.440756	0.633362	NALTREXONE HYDROCHLORIDE
6.482874	-0.2588	-0.028217928	-2.821792806	-0.53376	0.593505	0.756848	DEFLAZACORT
6.513218	-0.39317	-0.028267365	-2.826736483	-0.47547	0.63445	0.788634	PROTAMINE SULFATE [10mg/ ml]
6.507856	-0.31916	-0.028298593	-2.829859272	-1.08744	0.276843	0.480324	DIACETAMATE
6.469822	-0.2949	-0.028356377	-2.835637711	-2.22171	0.026303	0.0918	HYDROFLUMETHIAZIDE
6.522444	-0.3487	-0.028395083	-2.839508325	-1.09115	0.275208	0.478136	EPALRESTAT
6.4843	-0.25499	-0.028429463	-2.842946315	-0.62059	0.534868	0.710567	Decitabine
6.518514	-0.34074	-0.028504804	-2.850480442	-1.09536	0.273357	0.476051	NEVIRAPINE
6.494155	-0.35244	-0.028576124	-2.857612424	-0.63324	0.526576	0.704487	O-Acetyl-L-carnitine hydrochloride
6.489958	-0.26958	-0.02859131	-2.859131042	-0.78368	0.433226	0.625702	MIDODRINE HYDROCHLORIDE
6.49276	-0.27507	-0.028595358	-2.859535814	-0.78379	0.433161	0.625702	PIOGLITAZONE HYDROCHLORIDE
6.521138	-0.34605	-0.028645362	-2.864536246	-1.10076	0.270999	0.473404	HEPTAMINOL HYDROCHLORIDE
6.468347	-0.29667	-0.02870678	-2.870677985	-0.48286	0.629192	0.785149	Indacaterol Maleate
6.521138	-0.4105	-0.028804285	-2.880428455	-0.6383	0.52328	0.701544	Lycopene
6.477121	-0.25692	-0.028819088	-2.881908782	-1.10744	0.268104	0.469857	ADIPIC ACID
6.514548	-0.3151	-0.028834313	-2.883431297	-0.62943	0.529068	0.706528	Rimonabant
6.485721	-0.33404	-0.028953538	-2.895353775	-0.48701	0.626248	0.781665	HEPTACHLOR
6.471292	-0.29803	-0.028983927	-2.898392736	-2.27088	0.023154	0.083018	DIETHYLSTILBESTROL
6.480007	-0.25011	-0.029087504	-2.908750445	-0.79728	0.425286	0.618522	DROSPIRENONE
6.471292	-0.303	-0.029107247	-2.910724668	-0.4896	0.624417	0.780364	Apremilast (CC-10004)
6.50515	-0.30391	-0.029129444	-2.912944402	-0.55101	0.58163	0.74898	ADENOSINE TRIPHOSPHATE DISODIUM
6.498311	-0.29006	-0.029228623	-2.922862265	-0.55288	0.580345	0.748266	PIMAGEDINE HYDROCHLORIDE
6.510545	-0.30714	-0.02924154	-2.924154036	-0.63832	0.523266	0.701544	Rufinamide
6.480007	-0.25011	-0.029247884	-2.924788364	-0.80168	0.422738	0.616643	PRILOCAINE HYDROCHLORIDE
6.4843	-0.25851	-0.029279411	-2.927941078	-0.80254	0.422238	0.61609	CARBARSONE
6.518514	-0.40456	-0.029318839	-2.931883879	-0.49316	0.6219	0.778706	Tramadol·HCl
6.485721	-0.33404	-0.029593711	-2.959371135	-0.49778	0.618637	0.775569	Olopatadine
6.466868	-0.22639	-0.029649467	-2.964946666	-0.56084	0.574905	0.744251	LOBENDAZOLE
6.462398	-0.27908	-0.029780917	-2.978091659	-2.33332	0.019631	0.073494	DAPSONE
6.498311	-0.28592	-0.029868107	-2.986810703	-0.81868	0.412969	0.60811	CHLORMADINONE ACETATE
6.456366	-0.2036	-0.029988001	-2.998800109	-0.63954	0.522469	0.701191	NICARDIPINE HYDROCHLORIDE
6.506505	-0.30666	-0.030008025	-3.000802522	-0.56762	0.57029	0.740336	MIRTAZAPINE
6.482874	-0.32791	-0.030156088	-3.015608782	-0.50724	0.611985	0.771005	CIANIDANOL [+-catechin]
6.471292	-0.29803	-0.030159691	-3.015969069	-2.363	0.018128	0.068904	INOSITOL
6.519828	-0.3434	-0.030235203	-3.02352034	-1.16186	0.245293	0.443937	LEVOCARNITINE PROPIONATE HYDROCHLORIDE
6.465383	-0.2174	-0.030541901	-3.054190061	-0.6667	0.504961	0.687409	Vatalanib (PTK787) 2HCl
6.4843	-0.26029	-0.030553809	-3.055380903	-0.65161	0.514652	0.694706	RANITIDINE HYDROCHLORIDE
6.510545	-0.31484	-0.030795294	-3.079529373	-0.58252	0.560219	0.731528	ANAGRELIDE HYDROCHLORIDE
6.469822	-0.23019	-0.030844955	-3.084495538	-0.84546	0.397856	0.594503	EZETIMIBE
6.463893	-0.28227	-0.030856128	-3.08561284	-2.41756	0.015625	0.061585	CARISOPRODOL
6.519828	-0.40768	-0.030907119	-3.090711866	-0.6849	0.49341	0.676194	Pentostatin
6.494155	-0.35244	-0.030942353	-3.094235336	-0.68568	0.492917	0.675748	Adrenosterone
6.509203	-0.30448	-0.030947103	-3.094710269	-0.67555	0.499326	0.681383	Marimastat(BB-2516)
6.472756	-0.23686	-0.031047251	-3.104725083	-0.66213	0.507885	0.69012	SULFAMETHAZINE
6.468347	-0.29176	-0.031076544	-3.107654417	-2.43483	0.014899	0.059455	ROBENIDINE HYDROCHLORIDE
6.463893	-0.28709	-0.031090663	-3.109066276	-0.52296	0.601001	0.762419	OZAGREL HYDROCHLORIDE
6.480007	-0.24646	-0.031123447	-3.112344655	-0.6794	0.496885	0.679501	Nandrolone decanoate
6.5302	-0.3644	-0.031149229	-3.114922875	-1.19698	0.231314	0.427973	VINCAMINE
6.513218	-0.32025	-0.031333528	-3.133352783	-0.5927	0.553384	0.726188	DIETHYLTOLUAMIDE
6.513218	-0.39346	-0.031350179	-3.135017943	-0.69471	0.487235	0.671686	Sinomenine hydrochloride
6.481443	-0.25292	-0.031440035	-3.14400351	-0.86177	0.388816	0.586373	CEFUROXIME AXETIL
6.444045	-0.18994	-0.031450234	-3.145023395	-1.20855	0.226836	0.42356	KHELLIN
6.517196	-0.40202	-0.031465203	-3.146520337	-0.69726	0.485639	0.671112	Bifendatatum
6.471292	-0.30324	-0.031629565	-3.162956487	-0.7009	0.483363	0.668689	Eburnalritardo
6.510545	-0.38771	-0.031631236	-3.163123641	-0.70094	0.48334	0.668689	Verteporfin
6.507856	-0.3018	-0.031645291	-3.164529147	-0.69079	0.489697	0.673629	Nelfinavir Mesylate
6.499687	-0.28862	-0.031768737	-3.176873686	-0.87078	0.383876	0.582262	RANOLAZINE DIHYDROCHLORIDE
6.454845	-0.26299	-0.03183979	-3.18397896	-2.49463	0.012609	0.05202	NIACIN
6.471292	-0.29803	-0.032034802	-3.203480183	-2.50991	0.012076	0.050186	FUROSEMIDE
6.4843	-0.33098	-0.032057354	-3.205735428	-0.53922	0.589733	0.754268	AZINPHOS METHYL
6.506505	-0.37901	-0.032164206	-3.216420649	-0.71275	0.475999	0.66226	Anethole trithione
6.513218	-0.39317	-0.032179164	-3.217916399	-0.54127	0.588321	0.753427	TERAZOSIN HYDROCHLORIDE
6.49276	-0.27745	-0.032243087	-3.224308703	-0.68764	0.491681	0.674908	KETOPROFEN
6.506505	-0.37901	-0.032251314	-3.22513141	-0.71468	0.474805	0.661091	Tipiracil hydrochloride
6.487138	-0.26744	-0.032265905	-3.226590539	-0.61033	0.541641	0.716589	TOLTRAZURIL
6.499687	-0.36434	-0.03232903	-3.232902955	-0.7164	0.473742	0.660582	2-Aminobenzenesulfonamide
6.491362	-0.27461	-0.032333659	-3.233365908	-0.68957	0.490465	0.673962	TOLMETIN SODIUM
6.502427	-0.29101	-0.032373943	-3.237394344	-0.7067	0.479755	0.664892	Galeterone
6.457882	-0.20249	-0.03247034	-3.247034042	-0.7088	0.478448	0.664043	Nafamostat Mesylate
6.462398	-0.27908	-0.032491236	-3.249123608	-2.54568	0.010907	0.046574	OXIDOPAMINE HYDROCHLORIDE
6.481443	-0.25449	-0.032548525	-3.254852455	-0.69415	0.487587	0.67181	MEMANTINE HYDROCHLORIDE
6.460898	-0.27588	-0.032549342	-3.254934174	-2.55023	0.010765	0.046124	HYDROXYZINE PAMOATE
6.510545	-0.38742	-0.032555146	-3.255514582	-0.5476	0.58397	0.750733	Cabergoline
6.478566	-0.24865	-0.032606439	-3.260643851	-0.69539	0.486813	0.671434	IOHEXOL
6.450249	-0.25796	-0.032621386	-3.262138638	-0.72288	0.469752	0.655885	Tetrandrine
6.514548	-0.33271	-0.032731945	-3.27319453	-1.2578	0.208464	0.401404	CYPROTERONE
6.487138	-0.33708	-0.032736996	-3.273699606	-0.55065	0.581871	0.74906	EFLORNITHINE HYDROCHLORIDE HYDRATE
6.462398	-0.21584	-0.032900577	-3.290057703	-0.70166	0.482892	0.668398	INDOPROFEN
6.511883	-0.39059	-0.033006278	-3.30062781	-0.73141	0.464527	0.650898	Scopolamine N-oxide HBr
6.431364	-0.14979	-0.033150249	-3.315024904	-0.72364	0.469285	0.655627	Bosutinib (SKI-606)
6.506505	-0.30666	-0.033165723	-3.316572257	-0.62735	0.530427	0.707604	PRALIDOXIME CHLORIDE
6.474216	-0.23982	-0.033192471	-3.319247105	-0.70788	0.479017	0.664505	MEPHENESIN
6.501059	-0.36702	-0.033255073	-3.325507279	-0.55937	0.57591	0.744775	PHYSOSTIGMINE
6.474216	-0.30426	-0.033407877	-3.340787662	-2.61749	0.008858	0.039063	KITASAMYCINS [A1 shown]
6.50515	-0.3761	-0.033613939	-3.361393933	-0.74488	0.456346	0.643423	Ramatroban
6.465383	-0.2174	-0.033625497	-3.362549707	-0.73402	0.462938	0.649204	Riociguat (BAY 63-2521)
6.519828	-0.40768	-0.033643083	-3.364308286	-0.74552	0.455955	0.643421	Lappaconitine
6.498311	-0.29006	-0.033654594	-3.365459434	-0.6366	0.524384	0.702655	CITICOLINE
6.514548	-0.39632	-0.033660492	-3.366049153	-0.74591	0.455722	0.643421	Sodium Demethylcantharidate
6.523746	-0.41612	-0.033665055	-3.366505469	-0.74601	0.455661	0.643421	Methyl Aminolevulinate·HCl
6.50515	-0.29642	-0.033714005	-3.371400497	-0.73595	0.461762	0.648086	Imidapril HCl
6.471292	-0.29803	-0.033749259	-3.374925878	-2.64424	0.008187	0.036452	SEMUSTINE
6.474216	-0.24127	-0.033857284	-3.3857284	-0.64044	0.521889	0.700779	TILORONE
6.472756	-0.30115	-0.033945196	-3.394519591	-2.65959	0.007824	0.035137	NAPHAZOLINE HYDROCHLORIDE
6.503791	-0.31093	-0.033957345	-3.395734502	-1.30489	0.19193	0.380443	ALLYLTHIOUREA
6.519828	-0.33363	-0.033988019	-3.398801889	-0.64291	0.520283	0.699724	HYDRALAZINE HYDROCHLORIDE
6.485721	-0.33429	-0.034046988	-3.404698795	-0.75447	0.450565	0.639737	Ibudilast
6.454845	-0.26299	-0.034047188	-3.404718776	-2.66758	0.00764	0.034586	NORFLOXACIN
6.481443	-0.24931	-0.034156695	-3.415669532	-0.74561	0.455901	0.643421	TSU-68 (SU6668, Orantinib)
6.487138	-0.33708	-0.034189172	-3.418917181	-0.57508	0.565237	0.736016	Bemegride
6.525045	-0.41891	-0.034290128	-3.429012849	-0.75986	0.447337	0.638119	Mozavaptan
6.498311	-0.28283	-0.034369007	-3.436900661	-0.75025	0.453106	0.641388	SAR245409 (XL765)
6.511883	-0.32731	-0.034547813	-3.454781343	-1.32758	0.184317	0.36898	ITOPRIDE HYDROCHLORIDE
6.514548	-0.39632	-0.034548312	-3.454831159	-0.76558	0.443924	0.635064	Levobunolol·HCl
6.44248	-0.24104	-0.034548426	-3.454842577	-0.58112	0.561157	0.732006	Raltegravir
6.474216	-0.30426	-0.034567438	-3.456743786	-2.70834	0.006762	0.031219	KANAMYCIN A SULFATE
6.474216	-0.30426	-0.034603547	-3.460354728	-2.71117	0.006705	0.031009	DIOXYBENZONE
6.491362	-0.27233	-0.034629096	-3.462909583	-0.94918	0.34253	0.545215	CEFTAZIDIME
6.463893	-0.23013	-0.034784623	-3.478462332	-1.33668	0.181327	0.365086	NAFTOPIDIL
6.510545	-0.38771	-0.034805966	-3.480596568	-0.77129	0.440533	0.63322	Isoferulic acid
6.485721	-0.33404	-0.034837609	-3.483760947	-0.58599	0.557884	0.729595	Azacyclonol
6.488551	-0.26344	-0.034908552	-3.490855245	-0.76203	0.446045	0.637207	Fenticonazole Nitrate
6.463893	-0.21444	-0.034935134	-3.493513387	-0.76261	0.445699	0.63689	Tenofovir Disoproxil Fumarate
6.471292	-0.23535	-0.034952507	-3.495250661	-0.66115	0.508514	0.690284	PAMABROM
6.481443	-0.32483	-0.035055697	-3.505569683	-0.58966	0.555421	0.727752	Riluzole·HCl
6.440909	-0.23767	-0.035087584	-3.508758398	-0.59019	0.555062	0.727577	DICHLORODIPHENYLDICHLOROETHYLENE
6.518514	-0.34074	-0.035091914	-3.509191361	-1.34849	0.177501	0.360495	AMPYZINE SULFATE
6.499687	-0.29285	-0.035110332	-3.511033203	-0.66414	0.506602	0.689107	PYRIPROXYFEN
6.454845	-0.26763	-0.035206751	-3.520675134	-0.5922	0.553719	0.726188	Medetomidine HCl
6.501059	-0.36702	-0.035324574	-3.532457375	-0.59418	0.552392	0.725732	DELAPRIL HYDROCHLORIDE
6.482874	-0.25739	-0.035526069	-3.552606879	-0.75765	0.448659	0.638767	SULPIRIDE (S [-])
6.487138	-0.26605	-0.035559191	-3.555919068	-0.75836	0.448236	0.638734	LIDOCAINE HYDROCHLORIDE
6.49693	-0.28322	-0.035808164	-3.580816402	-0.9815	0.326348	0.532215	SUCRALFATE [5mM]
6.511883	-0.3903	-0.035823213	-3.582321301	-0.60257	0.546797	0.721095	RAMOPLANIN [A2 shown; 2mM]
6.481443	-0.25591	-0.035904574	-3.590457445	-0.67916	0.497035	0.679525	GLYCOPYRROLATE

6.480007	-0.25011	-0.035932112	-3.593211182	-0.98489	0.324676	0.531439	TERFENADINE
6.511883	-0.3903	-0.035976094	-3.597609433	-0.60514	0.545087	0.720133	Betaine
6.519828	-0.40768	-0.036087948	-3.608794802	-0.7997	0.423884	0.617408	Cyclovirobuxin D
6.478566	-0.24865	-0.036089013	-3.608901328	-0.76966	0.441503	0.633902	LASALOCID SODIUM
6.489958	-0.26958	-0.036125636	-3.612563582	-0.9902	0.322077	0.528706	RITANSERIN
6.49693	-0.28322	-0.036195368	-3.61953678	-0.99211	0.321144	0.528318	TERCONAZOLE
6.481443	-0.25591	-0.036259402	-3.625940238	-0.68587	0.492793	0.675748	COLISTIN SULFATE
6.494155	-0.27779	-0.036313982	-3.63139817	-0.99536	0.319561	0.526601	TELITHROMYCIN
6.517196	-0.40172	-0.036378952	-3.637895153	-0.61191	0.540595	0.715688	METHYL PARABEN
6.525045	-0.41891	-0.036440038	-3.644003821	-0.8075	0.419376	0.613536	Hesperetin
6.477121	-0.24572	-0.036447172	-3.644717201	-0.7773	0.436984	0.628852	PRAMOXINE HYDROCHLORIDE
6.466868	-0.2886	-0.036449463	-3.644946296	-2.8558	0.004293	0.021633	PENICILLIN V POTASSIUM
6.519828	-0.40768	-0.036468627	-3.646862677	-0.80814	0.419012	0.61348	Peramivir Trihydrate
6.495544	-0.29423	-0.036490508	-3.649050777	-1.40223	0.160846	0.337893	FENACLON
6.485721	-0.26317	-0.036543909	-3.654390885	-0.77936	0.435768	0.627781	CEFOPERAZONE
6.444045	-0.17976	-0.036549162	-3.654916224	-1.00181	0.316437	0.522294	AKLOMIDE
6.503791	-0.37317	-0.036565882	-3.656588161	-0.81029	0.417772	0.612385	Eprazinone dihydrochloride
6.475671	-0.24422	-0.036655655	-3.665565542	-0.69337	0.488078	0.672124	PYRIDOXINE HYDROCHLORIDE
6.474216	-0.23495	-0.036675131	-3.667513103	-0.80059	0.42337	0.617038	Tizanidine HCl
6.494155	-0.28028	-0.036883457	-3.68834568	-0.7866	0.431516	0.624819	SULFASALAZINE
6.49276	-0.27745	-0.036900923	-3.690092258	-0.78697	0.431297	0.624706	PHTHALYLSULFATHIAZOLE
6.475671	-0.24278	-0.036997813	-3.699781257	-0.78904	0.430089	0.623458	PIRENZEPINE HYDROCHLORIDE
6.482874	-0.32271	-0.037064778	-3.706477843	-2.90401	0.003684	0.019053	PROGESTERONE
6.480007	-0.3166	-0.037162061	-3.716206056	-2.91163	0.003595	0.018613	ESTRONE
6.489958	-0.28292	-0.03717207	-3.717206961	-1.42842	0.15317	0.327582	RAMIFENAZONE
6.519828	-0.3434	-0.037372844	-3.737284391	-1.43614	0.150963	0.324216	THIOCTIC ACID
6.459392	-0.20974	-0.037378857	-3.737885738	-0.79717	0.425355	0.618522	QUINACRINE HYDROCHLORIDE
6.450249	-0.25775	-0.037391061	-3.739106145	-0.62894	0.529389	0.706599	Saquinavir Mesylate
6.472756	-0.30615	-0.037446558	-3.744655804	-0.62987	0.528778	0.706528	BETAXALOL HYDROCHLORIDE
6.52763	-0.42416	-0.037468138	-3.746813829	-0.63023	0.528541	0.706528	Ethamsylate
6.487138	-0.26605	-0.037685944	-3.768594383	-0.80372	0.421561	0.615629	CEPHALEXIN
6.471292	-0.23535	-0.037774224	-3.777422413	-0.71453	0.474901	0.661091	BENOXINATE HYDROCHLORIDE
6.466868	-0.22491	-0.037781269	-3.778126874	-0.80575	0.420388	0.614557	PYRAZINAMIDE
6.474216	-0.25104	-0.037968205	-3.796820517	-1.45902	0.144561	0.314519	DEXFOSFOSERINE HYDROCHLORIDE
6.515874	-0.33539	-0.038077106	-3.80771056	-1.4632	0.143412	0.313523	THIOPHANATE METHYL
6.453318	-0.26435	-0.038140736	-3.814073613	-0.64155	0.521166	0.700176	Lypressin Acetate
6.480007	-0.25011	-0.038167168	-3.816716835	-1.04616	0.295489	0.499968	ETIDRONATE DISODIUM
6.488551	-0.26891	-0.038167431	-3.8167431	-0.81398	0.415654	0.610485	FENBUFEN
6.475671	-0.30736	-0.038175086	-3.817508592	-2.991	0.002781	0.014892	DIPHENYLPYRALINE HYDROCHLORIDE
6.513218	-0.39346	-0.038273687	-3.82736872	-0.84814	0.396362	0.593692	WY-14643 (Pirinixic Acid)
6.517196	-0.40172	-0.038384724	-3.838472448	-0.64565	0.518505	0.697882	Isosorbide
6.494155	-0.28165	-0.038563739	-3.856373907	-0.72946	0.465719	0.651854	ETHYNODIOL DIACETATE
6.456366	-0.19948	-0.038650947	-3.865094657	-0.84372	0.398827	0.594986	CAL-101 (Idelalisib, GS-1101)
6.475671	-0.30736	-0.038826895	-3.882689476	-3.04207	0.00235	0.012907	ESTRADIOL CYPIONATE
6.469822	-0.2949	-0.038874388	-3.887438752	-3.04579	0.002321	0.01279	CYCLOSERINE (D)
6.495544	-0.29423	-0.038878501	-3.887850099	-1.494	0.135176	0.300132	OLTIPRAZ
6.434569	-0.16099	-0.038895361	-3.889536076	-0.73574	0.461892	0.648092	NITROGLYCERIN
6.498311	-0.28592	-0.038927045	-3.892704462	-1.06698	0.285979	0.4908	HOMOSALATE
6.501059	-0.30539	-0.038949783	-3.894978277	-1.49674	0.134462	0.299454	ACETANILIDE
6.499687	-0.30262	-0.038984837	-3.898483674	-1.49808	0.134112	0.298934	AMPIROXICAM
6.510545	-0.3246	-0.039008747	-3.900874665	-1.499	0.133873	0.298663	FENTHION
6.487138	-0.33708	-0.039028096	-3.902809613	-0.65647	0.511519	0.692285	IVABRADINE HYDROCHLORIDE
6.49693	-0.29703	-0.039081771	-3.908177103	-1.50181	0.133147	0.297702	GUAIACOL
6.49693	-0.28591	-0.039082995	-3.908299512	-0.83351	0.404557	0.600213	RITONAVIR
6.459392	-0.27268	-0.039112	-3.911199961	-3.06441	0.002181	0.012138	HOMATROPINE HYDROBROMIDE
6.448706	-0.25443	-0.039188662	-3.918866237	-0.65918	0.509783	0.690866	Regadenoson
6.487138	-0.26407	-0.039223066	-3.92230658	-1.0751	0.282331	0.487368	HYDROCORTISONE BUTYRATE
6.478566	-0.31353	-0.039227958	-3.922795823	-3.07349	0.002116	0.011864	MELPHALAN
6.474216	-0.30426	-0.039267566	-3.926756636	-3.0766	0.002094	0.011755	LEUCOVORIN CALCIUM
6.494155	-0.35217	-0.039340663	-3.934066341	-0.66173	0.508143	0.690217	Sertaconazole
6.498311	-0.36138	-0.039349464	-3.934946434	-0.87198	0.383222	0.582224	Tizoxanide
6.510545	-0.30714	-0.039374058	-3.937405784	-0.8595	0.390063	0.587466	Lapatinib
6.49693	-0.28322	-0.039380672	-3.938067209	-1.07942	0.280401	0.485018	GADOTERIDOL
6.523746	-0.41581	-0.039415814	-3.941581426	-0.663	0.507333	0.689553	Aprotinin
6.475671	-0.30736	-0.039425266	-3.942526643	-3.08895	0.002009	0.011376	POTASSIUM p-AMINOBENZOATE
6.460898	-0.2143	-0.039553992	-3.955399223	-0.74819	0.454343	0.64243	THIOPENTAL SODIUM
6.503791	-0.29372	-0.039558518	-3.955851754	-0.86353	0.387846	0.585255	Raltitrexed
6.495544	-0.2831	-0.039640185	-3.964018534	-0.84539	0.397892	0.594503	ALLANTOIN
6.440909	-0.23786	-0.039662611	-3.966261138	-0.87892	0.379447	0.57924	Diatrizoate Meglumine
6.487138	-0.26605	-0.039712308	-3.971230793	-0.84693	0.397034	0.594042	ESOMEPRAZOLE POTASSIUM
6.434569	-0.22403	-0.039717397	-3.971739667	-0.66807	0.50409	0.686603	Otilonium Bromide
6.477121	-0.31045	-0.039717867	-3.971786699	-3.11188	0.001859	0.010623	CHLOROTHIAZIDE
6.477121	-0.31045	-0.039732146	-3.973214648	-3.113	0.001852	0.010595	MANNITOL
6.518514	-0.32298	-0.039773335	-3.977333454	-0.86822	0.385274	0.583438	Eslicarbazepine Acetate
6.472756	-0.30115	-0.039851978	-3.985197766	-3.12239	0.001794	0.010343	LEVOMILNACIPRAN HYDROCHLORIDE
6.489958	-0.26623	-0.039896579	-3.989657942	-0.87091	0.383803	0.582262	Cobimetinib (GDC-0973, RG7420)
6.471292	-0.29803	-0.039921228	-3.992122804	-3.12781	0.001761	0.010166	PILOCARPINE NITRATE
6.463893	-0.28227	-0.03994553	-3.994553003	-3.12972	0.00175	0.010123	PHENAZOPYRIDINE HYDROCHLORIDE
6.4133	-0.17845	-0.040077791	-4.00777908	-0.88812	0.374479	0.57544	Omeprazole sulfide
6.457882	-0.26946	-0.040099376	-4.009937623	-3.14177	0.001679	0.009759	DEFEROXAMINE MESYLATE
6.485721	-0.27434	-0.040177114	-4.017711383	-1.5439	0.122613	0.282681	BROMOPRIDE
6.421604	-0.19615	-0.04024738	-4.024738011	-0.67698	0.498417	0.680505	Tenofovir Alafenamide (GS-7340)
6.459392	-0.20549	-0.040264422	-4.026442235	-0.87894	0.379434	0.57924	Perampanel
6.478566	-0.2473	-0.04043993	-4.043993001	-1.10845	0.267666	0.469819	SUPLATAST TOSYLATE
6.521138	-0.3282	-0.040495179	-4.049517904	-0.88398	0.376709	0.576779	Loteprednol etabonate
6.495544	-0.2831	-0.040527677	-4.052767658	-0.86432	0.387412	0.584773	SULFAPYRIDINE
6.475671	-0.30736	-0.040581214	-4.058121424	-3.17952	0.001475	0.008722	AMILORIDE HYDROCHLORIDE
6.499687	-0.29285	-0.040597781	-4.059778119	-0.76794	0.442524	0.634657	METHYCLOTHIAZIDE
6.528917	-0.36181	-0.04065842	-4.065841972	-1.56239	0.118195	0.275573	FENDILINE HYDROCHLORIDE
6.519828	-0.40738	-0.040689083	-4.068908293	-0.68441	0.493714	0.676289	GLYBURIDE
6.49693	-0.28591	-0.040720791	-4.072079145	-0.86844	0.385154	0.583429	DICLAZURIL
6.523746	-0.35134	-0.040828179	-4.082817889	-1.56892	0.116667	0.273325	d-LIMONENE
6.513218	-0.39317	-0.040864638	-4.086463762	-0.68737	0.491852	0.674962	Difluprednate
6.510545	-0.38742	-0.040901246	-4.090124585	-0.68798	0.491464	0.674791	DORZOLAMIDE
6.474216	-0.30929	-0.041251798	-4.125179783	-0.69388	0.487759	0.671866	Sodium butyrate
6.471292	-0.23306	-0.041282362	-4.128236213	-1.13154	0.257826	0.457881	ALTRETAMINE
6.488551	-0.26683	-0.041371172	-4.13711722	-1.13398	0.256804	0.456857	MILNACIPRAN HYDROCHLORIDE
6.507856	-0.30939	-0.041384131	-4.138413052	-0.78281	0.433737	0.626088	GLIPIZIDE
6.526339	-0.42139	-0.041395893	-4.139589348	-0.6963	0.48624	0.671174	CORTEXOLONE ACETATE
6.469822	-0.23019	-0.041436405	-4.143640508	-1.13577	0.256054	0.455841	ADIPHENINE HYDROCHLORIDE
6.495544	-0.28051	-0.041441034	-4.14410345	-1.13589	0.256001	0.455841	SARAFLOXACIN HYDROCHLORIDE
6.487138	-0.26605	-0.041616859	-4.161685857	-0.88755	0.374784	0.57544	TRIMETHOPRIM
6.501059	-0.2913	-0.041810481	-4.181048103	-1.14602	0.251787	0.45232	ENOXACIN
6.488551	-0.26891	-0.041871409	-4.187140895	-0.89298	0.371869	0.57314	CIMETIDINE
6.480007	-0.253	-0.041909827	-4.190982654	-0.79276	0.42792	0.621273	DIAZOXIDE
6.471292	-0.23306	-0.041956034	-4.195603429	-1.15001	0.25014	0.450153	THALIDOMIDE
6.481443	-0.24931	-0.041974155	-4.1974155	-0.91626	0.35953	0.560722	Parecoxib
6.488551	-0.28006	-0.04202037	-4.202037048	-1.61473	0.106369	0.254924	BRIVUDINE
6.507856	-0.31916	-0.042030038	-4.203003765	-1.6151	0.106289	0.254894	ACECLOFENAC
6.487138	-0.2772	-0.042090538	-4.209053818	-1.61743	0.105786	0.253922	IDAZOXAN HYDROCHLORIDE
6.463893	-0.28227	-0.04211209	-4.211209023	-3.29946	0.000969	0.006019	AMOXICILLIN
6.487138	-0.26605	-0.042130041	-4.213004066	-0.89849	0.368923	0.569627	BETAMETHASONE
6.469822	-0.2949	-0.042151678	-4.215167792	-3.30257	0.000958	0.00596	NEOMYCIN TRISULFATE
6.471292	-0.23389	-0.042177104	-4.217710431	-0.8995	0.368388	0.569317	MERBROMIN
6.477121	-0.24572	-0.042278787	-4.227878739	-0.90167	0.367235	0.568392	RIBAVIRIN
6.469822	-0.2949	-0.042383704	-4.238370377	-3.32075	0.000898	0.005628	AMINOGLUTETHIMIDE
6.44248	-0.24124	-0.04265279	-4.265279021	-0.94518	0.344569	0.547062	Hyperoside
6.485721	-0.26129	-0.042732885	-4.273288533	-1.1713	0.241477	0.440144	AZELASTINE HYDROCHLORIDE
6.525045	-0.41891	-0.042901226	-4.290122618	-0.95068	0.341766	0.544782	Disodium clodronate tetrahydrate
6.485721	-0.25781	-0.04311583	-4.311583019	-0.94118	0.346611	0.549256	Temocapril HCl
6.477121	-0.31045	-0.043196159	-4.319615891	-3.3844	0.000713	0.004571	DOMPERIDONE
6.521138	-0.33629	-0.043370757	-4.337075722	-0.82039	0.411993	0.607373	BUTYLATED HYDROXYANISOLE
6.514548	-0.39603	-0.043376059	-4.337605874	-0.72961	0.465629	0.651854	EMPAGLIFLOZIN
6.485721	-0.26317	-0.043398949	-4.339894942	-0.92555	0.354677	0.556186	VALDECOXIB
6.502427	-0.29101	-0.043417002	-4.341700221	-0.94776	0.343253	0.545963	Dabrafenib (GSK2118436)
6.457882	-0.20668	-0.043515348	-4.351534777	-0.92804	0.353388	0.554787	AZELAIC ACID
6.478566	-0.31353	-0.043568953	-4.356895262	-3.41361	0.000641	0.004134	CANAGLIFLOZIN
6.513218	-0.39317	-0.04360092	-4.360092046	-0.73339	0.46332	0.649561	IMATINIB
6.487138	-0.26063	-0.043600976	-4.360097565	-0.95177	0.341212	0.544238	GS-9973
6.517196	-0.33807	-0.043628177	-4.362817682	-1.67651	0.093638	0.234094	PASINIAZID
6.495544	-0.28051	-0.04364859	-4.364859029	-1.1964	0.23154	0.427973	THONZYLAMINE HYDROCHLORIDE

6.487138	-0.26605	-0.043724457	-4.372445714	-0.9325	0.35108	0.552916	MEBENDAZOLE
6.482874	-0.25572	-0.043813593	-4.381359347	-1.20092	0.22978	0.426725	NABUMETONE
6.521138	-0.4105	-0.043837277	-4.38372774	-0.97142	0.331337	0.53634	Magnolol
6.498311	-0.28592	-0.043901316	-4.390131558	-1.20333	0.228849	0.425456	DIPYRONE
6.489958	-0.27177	-0.043905997	-4.390599691	-0.93637	0.349084	0.551298	NITARSONE
6.49693	-0.28591	-0.04400432	-4.400432011	-0.93847	0.348005	0.550577	TRETINOIN
6.487138	-0.26744	-0.044025211	-4.402521098	-0.83277	0.404974	0.600483	FAMPRIDINE
6.478566	-0.31353	-0.044068623	-4.406862278	-3.45276	0.000555	0.003605	ACETAZOLAMIDE
6.514548	-0.39603	-0.044164961	-4.416496133	-0.74288	0.457555	0.644302	ALGINIC ACID [Mol Wt ~200,000; monomers shown]
6.523746	-0.35134	-0.044211933	-4.421193326	-1.69895	0.089329	0.226584	LOXOPROFEN
6.510545	-0.30714	-0.044277892	-4.427789224	-0.96655	0.333769	0.537727	Silodosin
6.525045	-0.35397	-0.044397761	-4.439776054	-1.70609	0.087992	0.224324	PROTIONAMIDE
6.494155	-0.28028	-0.044446762	-4.444676168	-0.9479	0.34318	0.545963	HYDRASTINE (1R, 9S)
6.487138	-0.26063	-0.044621391	-4.462139139	-0.97405	0.330032	0.535923	Lacidipine
6.515874	-0.39888	-0.044657747	-4.465774659	-0.75117	0.452552	0.640958	Aclidinium Bromide
6.514548	-0.39632	-0.044723805	-4.472380514	-0.99107	0.321651	0.528346	Zoledronic Acid Monohydrate
6.485721	-0.26317	-0.044740221	-4.474022065	-0.95416	0.340003	0.543281	SULFACETAMIDE
6.515874	-0.39918	-0.044753808	-4.47538076	-0.99173	0.321327	0.528322	Potassium guaiacolsulfonate
6.498311	-0.36111	-0.044758105	-4.475810482	-0.75286	0.451536	0.640229	Escitalopram
6.528917	-0.42693	-0.044831572	-4.483157167	-0.75409	0.450794	0.639857	SODIUM PHENYLACETATE
6.522444	-0.41331	-0.044872992	-4.487299183	-0.99438	0.32004	0.527221	Istradefylline
6.471292	-0.23306	-0.045139879	-4.513987913	-1.23728	0.215984	0.411005	AMCINONIDE
6.450249	-0.19119	-0.045161985	-4.516198468	-0.96315	0.33547	0.539294	PIMOZIDE
6.482874	-0.25739	-0.045190747	-4.519074676	-0.96377	0.335162	0.539124	PIPERIDOLATE HYDROCHLORIDE
6.49276	-0.28859	-0.045276635	-4.527663481	-1.73986	0.081884	0.213139	IPRONIAZID PHOSPHATE
6.528917	-0.42724	-0.045326736	-4.532673574	-1.00443	0.315171	0.521389	Paritaprevir
6.491362	-0.27461	-0.045349298	-4.534929766	-0.96715	0.33347	0.537727	beta-CYCLODEXTRIN
6.49693	-0.28322	-0.045379615	-4.53796152	-1.24385	0.213555	0.408874	THIAMPHENICOL
6.437751	-0.16583	-0.045596336	-4.559633641	-0.97242	0.330843	0.536321	PROPYLTHIOURACIL
6.475671	-0.24422	-0.045810232	-4.581023233	-0.86654	0.386197	0.584123	TADALAFIL
6.515874	-0.39918	-0.045816596	-4.581659633	-1.01529	0.309969	0.515441	4-Hydroxyantipyrine
6.460898	-0.27588	-0.045956461	-4.595646076	-3.60067	0.000317	0.002184	DICLOFENAC SODIUM
6.514548	-0.32294	-0.04597338	-4.597338018	-0.86962	0.384507	0.582621	NATAMYCIN
6.472756	-0.23686	-0.045993764	-4.599376407	-0.98089	0.326645	0.532215	ARECOLINE HYDROBROMIDE
6.481443	-0.25449	-0.046055134	-4.605513381	-0.9822	0.326	0.531906	SISOMICIN SULFATE
6.488551	-0.26891	-0.046084469	-4.608446867	-0.98283	0.325692	0.531906	BALSALAZIDE DISODIUM
6.488551	-0.26683	-0.046123349	-4.612334859	-1.26423	0.206146	0.397863	KETANSERIN
6.510545	-0.31484	-0.04612797	-4.612797013	-0.87255	0.382911	0.582152	FLUNIXIN MEGLUMINE
6.462398	-0.27908	-0.046155881	-4.615588097	-3.61629	0.000299	0.002079	MERCAPTOPURINE
6.474216	-0.30426	-0.046179474	-4.617947419	-3.61814	0.000297	0.00207	FURAZOLIDONE
6.478566	-0.31353	-0.046271394	-4.627139386	-3.62534	0.000289	0.002021	BENZTHIAZIDE
6.477121	-0.31045	-0.046327159	-4.632715905	-3.62971	0.000284	0.001993	BENSERAZIDE HYDROCHLORIDE
6.5302	-0.42969	-0.046394076	-4.639407608	-0.78037	0.435171	0.62745	CLORGILINE HYDROCHLORIDE
6.499687	-0.29285	-0.046432727	-4.643272694	-0.87831	0.379775	0.579568	BURAMATE
6.4133	-0.17845	-0.046443586	-4.644358648	-1.02918	0.303395	0.507842	Testosterone Phenylpropionate
6.495544	-0.28051	-0.046649314	-4.664931414	-1.27865	0.20102	0.391786	DABIGATRAN ETEXILATE MESYLATE
6.50515	-0.29931	-0.046706932	-4.670693165	-1.28023	0.200464	0.391448	BUTACAINE SULFATE
6.459392	-0.27764	-0.046765175	-4.676517474	-1.03631	0.300059	0.504347	Daurisoline
6.507856	-0.3046	-0.046889535	-4.688953461	-1.28524	0.19871	0.389956	ACETOPHENAZINE MALEATE
6.50515	-0.31368	-0.047069636	-4.706963588	-1.80876	0.070488	0.192172	DICHLORISONE ACETATE
6.471292	-0.29803	-0.047187676	-4.718767621	-3.69714	0.000218	0.001561	PRIMIDONE
6.498311	-0.29983	-0.047281247	-4.728124674	-1.81689	0.069234	0.189864	CHLORPHENIRAMINE MALEATE
6.480007	-0.24646	-0.047331156	-4.733115643	-1.0332	0.30151	0.505478	Resveratrol
6.499687	-0.29151	-0.047334238	-4.733423814	-1.00948	0.312744	0.518814	ENALAPRIL MALEATE
6.510545	-0.38771	-0.047459307	-4.745930748	-1.05169	0.292943	0.497366	Bromisoval
6.49693	-0.28727	-0.047477334	-4.747733368	-0.89807	0.369148	0.569696	PROTRYPTYLINE HYDROCHLORIDE
6.475671	-0.24278	-0.047572873	-4.757287309	-1.01457	0.310311	0.515841	PHENACETIN
6.471292	-0.23535	-0.047610326	-4.761032579	-0.90059	0.367809	0.568937	MUPIROCIN
6.519828	-0.40768	-0.047781738	-4.778173766	-1.05883	0.289676	0.494051	Cevimeline hydrochloride hemihydrate
6.511883	-0.32731	-0.047822307	-4.78223066	-1.83768	0.066109	0.183187	BARBITAL
6.502427	-0.2984	-0.047856845	-4.785684547	-0.90525	0.365334	0.566386	FOMEPIZOLE HYDROCHLORIDE
6.477121	-0.31045	-0.047873404	-4.787340435	-3.75086	0.000176	0.001282	ORPHENADRINE CITRATE
6.503791	-0.29665	-0.047873697	-4.787369735	-1.31221	0.189449	0.376837	SULFANILATE ZINC
6.474216	-0.23982	-0.047977615	-4.797761507	-1.0232	0.306212	0.510354	SODIUM SALICYLATE
6.463893	-0.28227	-0.04800189	-4.800189048	-3.76093	0.000169	0.001233	CYCLIZINE
6.451786	-0.20562	-0.04806452	-4.806451954	-1.84699	0.064748	0.180464	CAPREOMYCIN SULFATE [4mM]
6.465383	-0.2219	-0.048124052	-4.812405228	-1.02633	0.304738	0.508891	NALOXONE HYDROCHLORIDE
6.447158	-0.18118	-0.048230673	-4.823067322	-1.05284	0.292416	0.497068	Tebipenem Pivoxil
6.509203	-0.31212	-0.048261592	-4.826159156	-0.9129	0.361293	0.562564	CLOZAPINE
6.488551	-0.2703	-0.04843794	-4.843793969	-0.91624	0.359541	0.560722	CLOMIPRAMINE HYDROCHLORIDE
6.487138	-0.33708	-0.048476052	-4.847605218	-0.81539	0.414847	0.610174	TEBUCONAZOLE
6.509203	-0.38453	-0.048642226	-4.86422261	-0.81819	0.413249	0.608348	BRIMONIDINE
6.480007	-0.3166	-0.048654009	-4.865400872	-3.81202	0.000138	0.001027	CEPHAPIRIN SODIUM
6.49276	-0.28859	-0.048657729	-4.865772925	-1.86979	0.061513	0.173173	PRASTERONE ACETATE
6.523746	-0.34157	-0.04867673	-4.867673013	-0.92076	0.357177	0.557814	PREGNENOLONE SUCCINATE
6.480007	-0.25157	-0.048730967	-4.873096727	-1.03927	0.29868	0.502973	AZAPERONE
6.515874	-0.39888	-0.048740864	-4.874086378	-0.81985	0.412303	0.607479	Fenoprofen Calcium
6.528917	-0.42693	-0.048829212	-4.882921223	-0.82133	0.411456	0.607373	PROCARBAZINE HYDROCHLORIDE
6.491362	-0.27233	-0.048852784	-4.885278357	-1.33905	0.180555	0.36424	MEPRYLCAINE HYDROCHLORIDE
6.510545	-0.3246	-0.048870513	-4.887051317	-1.87796	0.060386	0.171061	CHLORPYRIFOS
6.4843	-0.25499	-0.04887216	-4.88721601	-1.06684	0.286044	0.4908	Rucaparib (AG-014699,PF-01367338)
6.481443	-0.25449	-0.048930457	-4.893045711	-1.04352	0.296706	0.500886	NITRENDIPINE
6.469822	-0.2949	-0.048961405	-4.896140477	-3.83611	0.000125	0.000937	DIBENZOTHIOPHENE
6.471292	-0.30324	-0.048991712	-4.899171181	-1.08565	0.277636	0.48121	Eptifibatide
6.503791	-0.37289	-0.04914879	-4.914879013	-0.82671	0.408401	0.60434	Calcipotriene
6.513218	-0.32025	-0.049191647	-4.919164687	-0.9305	0.352114	0.554033	ACAMPROSATE CALCIUM
6.50515	-0.30391	-0.049306413	-4.930641258	-0.93267	0.350991	0.552916	BETAMETHASONE SODIUM PHOSPHATE
6.485721	-0.33404	-0.049330326	-4.933032601	-0.82976	0.406673	0.602304	Vigabatrin
6.491362	-0.27599	-0.04934417	-4.934417011	-0.93338	0.350623	0.552916	ITRACONAZOLE HYDROCHLORIDE
6.494155	-0.27779	-0.049421859	-4.942185903	-1.35465	0.17553	0.358768	MEPIVACAINE HYDROCHLORIDE
6.498311	-0.28871	-0.0495895	-4.958950038	-1.05758	0.290248	0.494232	SULFATHIAZOLE
6.507856	-0.30939	-0.049656259	-4.965625947	-0.93929	0.347584	0.550288	ASPARAGINE (L) HYDRATE
6.517196	-0.40172	-0.04967137	-4.967137008	-0.8355	0.403436	0.599244	Levofloxacin·HCl
6.487138	-0.26407	-0.049767887	-4.976788739	-1.36413	0.172526	0.354611	VINPOCETINE
6.471292	-0.23389	-0.049838974	-4.983897426	-1.0629	0.287828	0.492211	alpha-TOCHOPHEROL [4 mM]
6.518514	-0.32298	-0.049839406	-4.98394057	-1.08795	0.276616	0.480167	Cobicistat (GS-9350)
6.478566	-0.31353	-0.049854303	-4.985430259	-3.90606	9.38E-05	0.000717	DESIPRAMINE HYDROCHLORIDE
6.522444	-0.3487	-0.049857082	-4.985708245	-1.91587	0.055381	0.160926	PEMPIDINE TARTRATE
6.534026	-0.43792	-0.049884299	-4.988429895	-0.83908	0.401424	0.597989	Eprosartan Mesylate
6.515874	-0.39888	-0.049900225	-4.990022517	-0.83935	0.401273	0.597939	ANAZOLENE SODIUM
6.511883	-0.32731	-0.049975827	-4.99758275	-1.92044	0.054803	0.159729	CARMOFUR
6.481443	-0.31966	-0.05001401	-5.001400987	-3.91858	8.91E-05	0.000685	CORTISONE ACETATE
6.480007	-0.3166	-0.050084755	-5.008475509	-3.92412	8.70E-05	0.000671	NITROMIDE
6.52763	-0.34943	-0.050175118	-5.017511801	-0.9491	0.34257	0.545215	CASANTHRANOL [cascaroside A shown]
6.478566	-0.31353	-0.050260251	-5.026025059	-3.93787	8.22E-05	0.000638	HYDROCORTISONE
6.507856	-0.31916	-0.050276758	-5.027675776	-1.932	0.053359	0.157283	SILIBININ
6.509203	-0.30448	-0.050291067	-5.029106732	-1.09781	0.272286	0.47467	Telbivudine
6.494155	-0.28028	-0.050525329	-5.052532948	-1.07754	0.281241	0.486306	MEFEXAMIDE HYDROCHLORIDE
6.507856	-0.38164	-0.050538486	-5.053848563	-0.85009	0.395278	0.592627	VALETHAMATE BROMIDE
6.522444	-0.41301	-0.050606141	-5.060614051	-0.85122	0.394645	0.591852	GRANISETRON HYDROCHLORIDE
6.480007	-0.32175	-0.050686349	-5.068634916	-0.85257	0.393896	0.591596	ASTEMIZOLE
6.49693	-0.28322	-0.050742516	-5.074251565	-1.39085	0.164272	0.342868	MOXIFLOXACIN HYDROCHLORIDE
6.404834	-0.09707	-0.050754592	-5.07545918	-1.10793	0.267891	0.469819	Cinacalcet HCl
6.506505	-0.29912	-0.05084236	-5.084235969	-1.10985	0.267065	0.469242	Fulvestrant
6.491362	-0.27461	-0.050912815	-5.091281483	-1.0858	0.277567	0.48121	PHENTOLAMINE HYDROCHLORIDE
6.510545	-0.38742	-0.051170892	-5.117089164	-0.86072	0.389391	0.586894	DEXRAZOXANE
6.463893	-0.21859	-0.051226394	-5.122639428	-1.40411	0.160287	0.337307	TEPOXALIN
6.460898	-0.20848	-0.051247874	-5.124787387	-1.1187	0.263268	0.464325	Xylitol
6.481443	-0.31966	-0.051249759	-5.124975894	-4.0154	5.93E-05	0.000472	ANTHRALIN
6.509203	-0.38482	-0.051254482	-5.12544822	-1.13579	0.256045	0.455841	Isocarboxazid
6.532754	-0.43518	-0.051425179	-5.142517914	-0.865	0.387039	0.584773	CEFCAPENE PIVOXIL HYDROCHLORIDE
6.472756	-0.30115	-0.051535567	-5.153556667	-4.03779	5.40E-05	0.000434	DOXEPIN HYDROCHLORIDE
6.502427	-0.29707	-0.051551569	-5.155156906	-1.09942	0.271584	0.474253	CHOLECALCIFEROL
6.532754	-0.36958	-0.051605693	-5.160569298	-1.98307	0.04736	0.14546	DICHLOROPHEN
6.481443	-0.31966	-0.051622537	-5.162253665	-4.0446	5.24E-05	0.000423	NOREPINEPHRINE TARTRATE
6.502427	-0.29707	-0.051638529	-5.163852939	-1.10128	0.270776	0.473326	FOSCARNET SODIUM
6.471292	-0.22914	-0.051656841	-5.165684071	-1.12763	0.259478	0.459538	Cilomilast
6.519828	-0.40738	-0.051734042	-5.173404221	-0.8702	0.384194	0.582491	BENACTYZINE HYDROCHLORIDE
6.518514	-0.34074	-0.051781885	-5.178188479	-1.98984	0.046609	0.143584	DIMINAZENE ACETURATE
6.507856	-0.38192	-0.051840181	-5.184018059	-1.14877	0.250652	0.45061	Glycopyrrolate Iodide
6.469822	-0.2949	-0.051880469	-5.188046871	-4.06481	4.81E-05	0.000391	DIHYDROERGOTAMINE MESYLATE

6.491362	-0.28576	-0.051908958	-5.190895769	-1.99472	0.046073	0.142362	MOGUISTEINE
6.444045	-0.17499	-0.052049416	-5.204941642	-1.1362	0.255874	0.455841	Telotristat Etiprate (LX 1606 Hippurate)
6.511883	-0.31755	-0.05220759	-5.220759001	-0.98755	0.323375	0.529879	MODALINE SULFATE
6.509203	-0.31212	-0.052208037	-5.220803704	-0.98755	0.323371	0.529879	NETILMICIN SULFATE
6.521138	-0.33629	-0.052212564	-5.221256351	-0.98764	0.323329	0.529879	FLUOROMETHOLONE
6.480007	-0.25011	-0.052217312	-5.221731229	-1.43127	0.152353	0.326331	CHLORPROTHIXENE HYDROCHLORIDE
6.498311	-0.28283	-0.052295769	-5.229576866	-1.14157	0.253631	0.453577	Cilazapril Monohydrate
6.50515	-0.30391	-0.05239684	-5.239683965	-0.99113	0.321624	0.528346	MIANSERIN HYDROCHLORIDE
6.491362	-0.27233	-0.052622739	-5.262273863	-1.44238	0.149195	0.321227	TRILOSTANE
6.477121	-0.31554	-0.052646595	-5.264659467	-0.88554	0.375863	0.576345	Ulipristal
6.494155	-0.28028	-0.052650105	-5.265010489	-1.12285	0.261501	0.462003	MESNA
6.52763	-0.42416	-0.052681868	-5.268186801	-0.88614	0.375543	0.5762	ORNITHINE aKETOGLUTARATE
6.503791	-0.29665	-0.05273273	-5.273273047	-1.4454	0.148346	0.320343	OXIGLUTATIONE
6.448706	-0.25464	-0.05277586	-5.277586009	-1.1695	0.242201	0.440993	Cefprozil
6.472756	-0.23205	-0.052872208	-5.287220763	-1.15416	0.248436	0.4482	Ibutilide Fumarate
6.495544	-0.28051	-0.053098367	-5.309836665	-1.45542	0.145553	0.315883	OLMESARTAN MEDOXOMIL
6.480007	-0.3166	-0.053127767	-5.312776735	-4.16254	3.15E-05	0.000266	GALLAMINE TRIETHIODIDE
6.487138	-0.2772	-0.053176714	-5.31767145	-2.04344	0.041009	0.130091	beta-NAPHTHOL
6.475671	-0.30736	-0.053254203	-5.325420311	-4.17244	3.01E-05	0.000256	IPRATROPIUM BROMIDE
6.525045	-0.41891	-0.053289652	-5.328965172	-1.18089	0.237647	0.434711	Abiraterone
6.468347	-0.22329	-0.053304319	-5.330431884	-1.16359	0.24459	0.443076	Bafetinib (INNO-406)
6.510545	-0.30986	-0.053309095	-5.330909464	-1.46119	0.143962	0.314113	URETHANE
6.472756	-0.24808	-0.053348819	-5.334881895	-2.05005	0.040359	0.128507	FTAXILIDE
6.480007	-0.25157	-0.053378509	-5.337850923	-1.13839	0.25496	0.454841	SULFACHLORPYRIDAZINE
6.499687	-0.29151	-0.053380446	-5.338044633	-1.13843	0.254942	0.454841	CEFUROXIME SODIUM
6.532754	-0.43518	-0.053569972	-5.356997152	-0.90108	0.367548	0.568705	ACETAMINOSALOL
6.526339	-0.35659	-0.05370927	-5.370926978	-2.0639	0.039027	0.125199	CARZENIDE
6.499687	-0.28862	-0.053709504	-5.370950374	-1.47217	0.140975	0.309251	BENZYL ALCOHOL
6.522444	-0.41301	-0.053730107	-5.373010693	-0.90377	0.366117	0.567075	GLYCYRRHIZIC ACID, AMMONIUM SALT
6.482874	-0.32271	-0.053778248	-5.37782478	-4.2135	2.51E-05	0.000219	LACTULOSE
6.532754	-0.4355	-0.053855608	-5.385560757	-1.19343	0.232701	0.429492	Ginsenoside Rc
6.506505	-0.37873	-0.053938389	-5.393838948	-0.90727	0.364262	0.565501	EMEDASTINE DIFUMARATE
6.44248	-0.18677	-0.053963666	-5.396366634	-2.07368	0.038109	0.122716	OXEDRINE
6.519828	-0.40768	-0.053976265	-5.397626516	-1.1961	0.231657	0.428034	Gastrodenol
6.460898	-0.28087	-0.054039644	-5.403964375	-1.19751	0.231109	0.427973	Naringin
6.50515	-0.37582	-0.054046496	-5.40464965	-0.90909	0.363302	0.564695	METACRESOL
6.502427	-0.29707	-0.05406445	-5.406444965	-1.15301	0.248905	0.448887	PIZOTYLINE MALATE
6.511883	-0.32731	-0.054140673	-5.414067335	-2.08048	0.037481	0.120859	TIMONACIC
6.448706	-0.18426	-0.054154068	-5.415406769	-1.18214	0.23715	0.434415	Sivelestat sodium
6.472756	-0.30115	-0.054216903	-5.421690256	-4.24787	2.16E-05	0.000191	NORTRIPTYLINE HYDROCHLORIDE
6.507856	-0.3018	-0.054217115	-5.42171154	-1.18352	0.236605	0.433889	Sotrastaurin
6.503791	-0.29372	-0.05444268	-5.444267995	-1.18844	0.23466	0.431869	Roscovitine (Seliciclib,CYC202)
6.489958	-0.27177	-0.054469211	-5.446921059	-1.16165	0.245379	0.443937	IODIPAMIDE
6.540329	-0.38492	-0.054535698	-5.453569838	-2.09566	0.036112	0.116979	DINITOLMIDE
6.489958	-0.26623	-0.054544183	-5.454418301	-1.19066	0.233789	0.43073	Clevidipine Butyrate
6.489958	-0.26958	-0.05456513	-5.456513035	-1.49562	0.134752	0.299579	PAROXETINE HYDROCHLORIDE
6.468347	-0.29176	-0.054676263	-5.467626295	-4.28386	1.84E-05	0.000164	GLUCOSAMINE HYDROCHLORIDE
6.462398	-0.21584	-0.054761666	-5.476166632	-1.16788	0.242854	0.441818	LEVETIRACETAM
6.475671	-0.23784	-0.054831475	-5.483147506	-1.19693	0.231335	0.427973	Ambrisentan
6.465383	-0.2903	-0.054864307	-5.486430742	-0.92285	0.356086	0.557283	Silver Sulfadiazine
6.501059	-0.2913	-0.054924715	-5.492471488	-1.50548	0.132201	0.296612	DICLORALUREA
6.494155	-0.28165	-0.054935311	-5.493531114	-1.03914	0.298738	0.502973	AMYLENE HYDRATE
6.4843	-0.26029	-0.054955104	-5.495510427	-1.17201	0.241194	0.439783	CEFORANIDE
6.501059	-0.29563	-0.055038695	-5.503869481	-1.0411	0.29783	0.502104	TICLOPIDINE HYDROCHLORIDE
6.494155	-0.28028	-0.055239496	-5.523949628	-1.17807	0.238767	0.435825	RACTOPAMINE HYDROCHLORIDE
6.514548	-0.39632	-0.055316895	-5.531689518	-1.22581	0.22027	0.416156	Coenzyme Q10 (CoQ10)
6.507856	-0.38164	-0.05547416	-5.547416035	-0.93311	0.350765	0.552916	Sodium Picosulfate
6.506505	-0.31642	-0.055551157	-5.555115694	-2.13468	0.032787	0.108513	BUCETIN
6.468347	-0.23915	-0.055577939	-5.557793924	-2.13571	0.032703	0.108375	TAMSULOSIN HYDROCHLORIDE
6.521138	-0.34605	-0.055729117	-5.572911682	-2.14152	0.032232	0.1073	EBSELEN
6.519828	-0.3434	-0.055877083	-5.587708333	-2.14721	0.031777	0.106408	CARSALAM
6.482874	-0.32271	-0.055885788	-5.588578782	-4.37863	1.19E-05	0.00011	HETACILLIN POTASSIUM
6.454845	-0.26763	-0.055888845	-5.588884506	-0.94008	0.347176	0.549981	Ropivacaine·HCl Monohydrate
6.462398	-0.2841	-0.055926942	-5.592694198	-1.23933	0.215224	0.410577	Dipivefrin hydrochloride
6.517196	-0.40202	-0.056009094	-5.600909385	-1.24115	0.21455	0.410217	Nicotine
6.471292	-0.23535	-0.05628929	-5.628929	-1.06475	0.286987	0.491251	NOCODAZOLE
6.522444	-0.33079	-0.056375657	-5.637565711	-1.23063	0.21846	0.413804	Tiagabine
6.510545	-0.31484	-0.056427016	-5.642701566	-1.06736	0.285809	0.4908	ORNIDAZOLE
6.482874	-0.26857	-0.056512421	-5.651242081	-2.17162	0.029884	0.101397	CLOFOCTOL
6.4843	-0.32575	-0.056513923	-5.651392312	-4.42784	9.52E-06	8.89E-05	PARACHLOROPHENOL
6.50515	-0.31368	-0.056592956	-5.659295618	-2.17472	0.029651	0.100776	ATROPINE OXIDE
6.499687	-0.29151	-0.056707016	-5.670701612	-1.20937	0.22652	0.423279	NEBIVOLOL HYDROCHLORIDE
6.514548	-0.39603	-0.05671312	-5.671312007	-0.95395	0.340111	0.543281	Risredonic Acid
6.510545	-0.38771	-0.056912343	-5.691234305	-1.26117	0.207249	0.399517	Methyl hesperidin
6.52763	-0.42416	-0.056962708	-5.696270807	-0.95814	0.33799	0.541461	Balsalazide
6.487138	-0.26407	-0.057041782	-5.704178248	-1.56351	0.117933	0.275252	LORATADINE
6.477121	-0.25692	-0.057160605	-5.716060495	-2.19653	0.028054	0.096655	BUFEXAMAC
6.478566	-0.25008	-0.057262621	-5.7262621	-1.08317	0.278735	0.482754	OXIRACETAM
6.459392	-0.20549	-0.057265259	-5.726525888	-1.25005	0.21128	0.405455	Nandrolone
6.506505	-0.30534	-0.057344791	-5.734479055	-1.22297	0.22134	0.417157	BRETYLIUM TOSYLATE
6.5302	-0.43	-0.057368003	-5.736800257	-1.27126	0.203635	0.394929	Bendazol
6.526339	-0.33853	-0.057557725	-5.755772502	-1.25644	0.208957	0.401923	Entecavir Hydrate
6.450249	-0.25775	-0.05772928	-5.772928039	-0.97104	0.331529	0.536482	Rupatadine Fumarate
6.468347	-0.29176	-0.05775633	-5.775632987	-4.52518	6.03E-06	5.79E-05	AZATHIOPRINE
6.507856	-0.38192	-0.05794692	-5.794691956	-1.28409	0.19911	0.390441	Mildronate
6.499687	-0.28862	-0.058008971	-5.800897098	-1.59002	0.111831	0.26563	ATORVASTATIN CALCIUM
6.515874	-0.39888	-0.058242668	-5.824266754	-0.97967	0.327247	0.532583	TETRABENAZINE
6.480007	-0.3166	-0.05845073	-5.845072967	-4.57959	4.66E-06	4.55E-05	NORGESTIMATE
6.49276	-0.27507	-0.058714963	-5.871496271	-1.60937	0.107536	0.257218	OLMESARTAN
6.502427	-0.29707	-0.058957498	-5.89574984	-1.25737	0.208621	0.401556	ESTRADIOL BENZOATE
6.531479	-0.43244	-0.059048092	-5.904809209	-0.99322	0.320602	0.527977	Teriflunomide
6.535294	-0.37472	-0.05913172	-5.913171981	-2.27227	0.02307	0.082774	PREGNENOLONE
6.501059	-0.2913	-0.059157825	-5.915782465	-1.62151	0.104909	0.252768	METHYLPREDNISOLONE SODIUM SUCCINATE
6.494155	-0.29141	-0.059254703	-5.925470288	-2.277	0.022786	0.082117	FEBUXOSTAT
6.50515	-0.29931	-0.059357806	-5.93578058	-1.62699	0.103739	0.251014	MODAFINIL
6.485721	-0.32878	-0.059358536	-5.935853603	-4.65072	3.31E-06	3.30E-05	PHENOLPHTHALEIN
6.519828	-0.40738	-0.059370003	-5.937000348	-0.99864	0.317971	0.52415	Mezlocillin Sodium
6.498311	-0.28283	-0.059467175	-5.946717508	-1.29812	0.194246	0.383992	Doxercalciferol
6.491362	-0.27461	-0.059513537	-5.951353676	-1.26922	0.204361	0.395886	VANCOMYCIN HYDROCHLORIDE
6.521138	-0.4102	-0.059688793	-5.968879337	-1.004	0.315379	0.521557	Ondansetron
6.515874	-0.32029	-0.05978572	-5.978572042	-1.63872	0.101272	0.247264	CLOBETASOL PROPIONATE
6.501059	-0.29563	-0.059868638	-5.9868638	-1.13246	0.257441	0.457514	ETHAMIVAN
6.477121	-0.24715	-0.05989698	-5.989698033	-1.133	0.257216	0.457272	LEVODOPA
6.521138	-0.3282	-0.059992242	-5.999224199	-1.30958	0.190337	0.37831	Camostat Mesilate
6.514548	-0.39632	-0.060141876	-6.014187609	-1.33273	0.18262	0.36696	Alizapride hydrochloride
6.4843	-0.25851	-0.060173086	-6.0173086	-1.64934	0.099079	0.244356	FOMEPIZOLE
6.498311	-0.28871	-0.060178729	-6.017872919	-1.28341	0.199348	0.390609	OFLOXACIN
6.5302	-0.3644	-0.060250485	-6.025048533	-2.31526	0.020598	0.076202	LOBELINE HYDROCHLORIDE
6.469822	-0.2949	-0.060261895	-6.026189459	-4.72149	2.34E-06	2.40E-05	AMITRIPTYLINE HYDROCHLORIDE
6.50515	-0.29931	-0.06034864	-6.034864021	-1.65415	0.098097	0.242286	CANDESARTAN CILEXTIL
6.503791	-0.29665	-0.060362234	-6.036223407	-1.65452	0.098022	0.242216	ISOSORBIDE MONONITRATE
6.499687	-0.29285	-0.06036902	-6.036901978	-1.14193	0.253485	0.453474	FLUTICASONE PROPIONATE
6.4843	-0.32575	-0.060373588	-6.037358839	-4.73025	2.24E-06	2.31E-05	BUSULFAN
6.506505	-0.31642	-0.060381647	-6.038164733	-2.32031	0.020324	0.075461	ELLAGIC ACID
6.50515	-0.30391	-0.060451	-6.045099978	-1.14348	0.252841	0.452638	HYMECHROME
6.515874	-0.32563	-0.060482944	-6.048294421	-1.14408	0.25259	0.452638	SULFISOXAZOLE ACETYL
6.511883	-0.31248	-0.060540588	-6.054058756	-1.65941	0.097033	0.240008	PROPAFENONE HYDROCHLORIDE
6.502427	-0.29707	-0.060552015	-6.055201472	-1.29137	0.196575	0.386512	STREPTOMYCIN SULFATE [5mM; 10% aq DMSO + 5mM ethanolamine]
6.537819	-0.4464	-0.060604185	-6.060418524	-1.34298	0.17928	0.362947	Nicotinic acid hydrazide
6.4843	-0.26169	-0.060774648	-6.077464825	-1.1496	0.250309	0.450153	CYCLOSERINE (L)
6.501059	-0.2913	-0.060810177	-6.081017734	-1.6668	0.095554	0.23761	IBANDRONATE SODIUM
6.534026	-0.36238	-0.060844116	-6.084411645	-1.15091	0.249768	0.449811	BEPHENIUM HYDROXYNAPTHOATE
6.531479	-0.43275	-0.060903657	-6.090365657	-1.34961	0.17714	0.360155	L-5-Hydroxytryptophan
6.5302	-0.42969	-0.060975719	-6.097571884	-1.02565	0.305059	0.509142	EPLERENONE
6.537819	-0.4464	-0.061001457	-6.10014574	-1.35178	0.176446	0.359721	Salvianolic acid B
6.518514	-0.34074	-0.061039981	-6.103998083	-2.3456	0.018996	0.071358	TRIMETAZIDINE DIHYDROCHLORIDE
6.44248	-0.17701	-0.061146626	-6.114662554	-1.15663	0.247422	0.446528	CLOFAZIMINE
6.475671	-0.31242	-0.061168352	-6.116835249	-1.02889	0.303533	0.507842	Tasisulam
6.49693	-0.28727	-0.061206687	-6.12066868	-1.15777	0.246958	0.446112	BROMPERIDOL
6.507856	-0.30939	-0.061313562	-6.131356163	-1.15979	0.246133	0.444987	TETRAMIZOLE HYDROCHLORIDE
6.50515	-0.30259	-0.061378787	-6.137878747	-1.309	0.190533	0.378552	TOBRAMYCIN

6.482874	-0.2588	-0.061415488	-6.141548824	-1.16172	0.245349	0.443937	ISOETHARINE MESYLATE
6.534026	-0.43792	-0.06144499	-6.144498985	-1.03354	0.301352	0.505379	Atosiban Acetate
6.528917	-0.42724	-0.061506796	-6.150679593	-1.36298	0.17289	0.35474	Deracil
6.509203	-0.32189	-0.061543009	-6.154300887	-2.36493	0.018033	0.068647	AFALANINE
6.471292	-0.29803	-0.061549533	-6.154953288	-4.82238	1.42E-06	1.51E-05	DIMENHYDRINATE
6.495544	-0.28051	-0.061560673	-6.156067279	-1.68737	0.091532	0.230746	PIPOBROMAN
6.495544	-0.28051	-0.061583658	-6.158365815	-1.688	0.091411	0.230555	AZLOCILLIN SODIUM
6.478566	-0.2436	-0.061661424	-6.166142351	-1.34602	0.178296	0.361679	Pimecrolimus
6.49276	-0.28859	-0.061745693	-6.174569274	-2.37272	0.017658	0.067768	PHENOLSULFONPHTHALEIN
6.439333	-0.23447	-0.061910644	-6.191064423	-1.37193	0.170086	0.352147	Trapidil
6.5302	-0.43	-0.062090172	-6.209017156	-1.37591	0.168851	0.350528	Asiatic acid
6.475671	-0.30736	-0.062368746	-6.236874566	-4.88657	1.03E-06	1.11E-05	ISOPROPAMIDE IODIDE
6.465383	-0.28544	-0.062430848	-6.243084816	-4.89143	1.00E-06	1.09E-05	DANTHRON
6.498311	-0.29983	-0.062435692	-6.243569207	-2.39924	0.016429	0.064332	ERDOSTEINE
6.525045	-0.41891	-0.06245713	-6.24571297	-1.38404	0.166347	0.345937	Loganin
6.498311	-0.28592	-0.062513639	-6.251363942	-1.71349	0.086622	0.222086	RIBOFLAVIN
6.495544	-0.2831	-0.06269087	-6.269087031	-1.33699	0.181227	0.365086	TRAMIPROSATE
6.456366	-0.19948	-0.062699747	-6.269974651	-1.36868	0.171098	0.352986	Bazedoxifene Acetate
6.506505	-0.29912	-0.062729841	-6.272984142	-1.36934	0.170893	0.35281	Gabexate Mesylate
6.472756	-0.30115	-0.062814771	-6.281477056	-4.92151	8.59E-07	9.52E-06	NORETHYNODREL
6.502427	-0.29707	-0.062818477	-6.281847664	-1.33971	0.18034	0.364233	METHACYCLINE HYDROCHLORIDE
6.487138	-0.3318	-0.063002413	-6.300241286	-4.93621	7.97E-07	8.89E-06	KETOCONAZOLE
6.488551	-0.28006	-0.063223894	-6.322389366	-2.42953	0.015119	0.060146	TIOGUANINE
6.488551	-0.33481	-0.063305737	-6.330573667	-4.95998	7.05E-07	7.90E-06	INDOMETHACIN
6.49276	-0.27745	-0.063365667	-6.336566667	-1.35138	0.176574	0.359754	ANISINDIONE
6.507856	-0.30939	-0.063369578	-6.33695782	-1.19868	0.230651	0.427719	PANTOTHENIC ACID(d) Na salt
6.510545	-0.38742	-0.063439792	-6.343979249	-1.06709	0.28593	0.4908	CLONIXIN
6.488551	-0.34012	-0.063779926	-6.377992615	-1.07281	0.283355	0.488806	Pilocarpine·HCl
6.465383	-0.29053	-0.063848263	-6.384826274	-1.41486	0.157108	0.333132	Sophoricoside
6.499687	-0.29151	-0.063925295	-6.392529457	-1.36331	0.172784	0.35474	SURAMIN HEXASODIUM
6.522444	-0.41331	-0.063930176	-6.393017621	-1.41668	0.156577	0.332782	Blonanserin
6.52763	-0.3592	-0.064030524	-6.403052391	-2.46052	0.013874	0.056108	CORTISONE
6.494155	-0.28028	-0.064100816	-6.410081605	-1.36706	0.171608	0.353432	RETINOL
6.518514	-0.40486	-0.064164794	-6.416479372	-1.42188	0.155062	0.331327	Lacosamide
6.499687	-0.28557	-0.064420896	-6.442089631	-1.40626	0.159648	0.33664	Vorapaxar
6.509203	-0.32189	-0.064585365	-6.458536509	-2.48184	0.01307	0.053451	LEVOMENTHOL
6.478566	-0.31353	-0.064596258	-6.459625825	-5.06109	4.17E-07	4.77E-06	ASPIRIN
6.472756	-0.23832	-0.064626472	-6.462647196	-1.22246	0.221534	0.417159	REPAGLINIDE
6.502427	-0.29398	-0.064666584	-6.46665837	-1.7725	0.076311	0.202757	RISEDRONATE SODIUM
6.498311	-0.28592	-0.064672224	-6.467222391	-1.77266	0.076286	0.202757	MEXILETINE HYDROCHLORIDE
6.523746	-0.35134	-0.064736537	-6.473653733	-2.48765	0.012859	0.052839	PERINDOPRILAT
6.416641	-0.12053	-0.064761829	-6.47618286	-1.4137	0.157451	0.33367	Luliconazole
6.517196	-0.40172	-0.064880588	-6.488058804	-1.09133	0.275129	0.478136	DROXYDOPA
6.475671	-0.30736	-0.064902383	-6.490238265	-5.08508	3.67E-07	4.26E-06	FLURBIPROFEN
6.5302	-0.35464	-0.064945065	-6.494506485	-1.22849	0.219265	0.414716	PROPIOLACTONE
6.426511	-0.14015	-0.064955713	-6.495571293	-1.41793	0.156211	0.332391	Lopinavir
6.515874	-0.32563	-0.065034367	-6.503436744	-1.23017	0.218632	0.413824	FIPRONIL
6.487138	-0.3318	-0.065127723	-6.512772342	-5.10273	3.35E-07	3.90E-06	EPHEDRINE (1R,2S) HYDROCHLORIDE
6.447158	-0.18492	-0.065242093	-6.52420931	-1.3914	0.164105	0.342807	SULFAQUINOXALINE SODIUM
6.526339	-0.34682	-0.06540503	-6.540502961	-1.23719	0.216018	0.411005	PELARGONIC ACID
6.489958	-0.33781	-0.065413706	-6.54137062	-5.12514	2.97E-07	3.47E-06	DESOXYCORTICOSTERONE ACETATE
6.50515	-0.37582	-0.065498305	-6.549830512	-1.10172	0.270584	0.473153	ATROPINE
6.506505	-0.30196	-0.065533484	-6.55334839	-1.79626	0.072453	0.195241	OXETHAZAINE
6.50515	-0.31368	-0.065684192	-6.568419161	-2.52407	0.011601	0.048684	PROXYPHYLLINE
6.523746	-0.33338	-0.065723023	-6.572302298	-1.43468	0.151378	0.324971	Enzalutamide (MDV3100)
6.487138	-0.26605	-0.065742492	-6.574249206	-1.40207	0.160895	0.337893	CAPECITABINE
6.477121	-0.25692	-0.065817177	-6.581717732	-2.52918	0.011433	0.048189	PIPERONYL BUTOXIDE
6.531479	-0.43275	-0.06587421	-6.58742097	-1.45976	0.144356	0.314379	Sodium [(2,3-dihydro-1,5-dimethyl-3-oxo-2-phenyl-1H-pyrazol-4-yl)methylamino]methanesulfonate
6.507856	-0.30808	-0.065970526	-6.59705265	-1.40693	0.159448	0.33664	KOJIC ACID
6.489958	-0.33781	-0.065972247	-6.597224697	-5.1689	2.35E-07	2.77E-06	EUGENOL
6.49276	-0.28859	-0.066064832	-6.606483166	-2.53869	0.011127	0.047159	CYROMAZINE
6.475671	-0.30736	-0.066131334	-6.613133395	-5.18136	2.20E-07	2.61E-06	METHYLDOPA
6.499687	-0.28557	-0.066291584	-6.629158357	-1.44709	0.147871	0.319587	Alprostadil
6.507856	-0.30939	-0.066415446	-6.641544566	-1.2563	0.209008	0.401923	DEXPANTHENOL
6.436163	-0.15933	-0.066561983	-6.656198264	-1.45299	0.146225	0.316966	Vandetanib (ZD6474)
6.494155	-0.28028	-0.066662743	-6.666274266	-1.42169	0.155115	0.331327	CARBETAPENTANE CITRATE
6.52763	-0.42416	-0.066723688	-6.672368777	-1.12233	0.261722	0.462235	Triptorelin Acetate
6.489958	-0.26623	-0.066757811	-6.675781107	-1.45727	0.145042	0.314936	Semagacestat (LY450139)
6.510545	-0.30714	-0.066847751	-6.684775065	-1.45923	0.144501	0.314519	Dextrose
6.537819	-0.4464	-0.06702445	-6.702445047	-1.48525	0.137478	0.303336	Batyl alcohol
6.489958	-0.27177	-0.067149462	-6.71494616	-1.43207	0.152123	0.326159	LOMEFLOXACIN HYDROCHLORIDE
6.536558	-0.36751	-0.067215575	-6.721557498	-1.27143	0.203574	0.394929	TRICLOSAN
6.488551	-0.33481	-0.067233871	-6.723387054	-5.26775	1.38E-07	1.67E-06	HALOPERIDOL
6.518514	-0.34074	-0.067320356	-6.732035559	-2.58694	0.009683	0.042015	ACIPIMOX
6.536558	-0.44369	-0.067322569	-6.732256877	-1.49185	0.135737	0.300855	Piceatannol
6.501059	-0.2913	-0.067403357	-6.740335677	-1.84752	0.064672	0.18035	NAFRONYL OXALATE
6.513218	-0.32025	-0.0674231	-6.742309981	-1.27536	0.202182	0.393303	D-LACTITOL MONOHYDRATE
6.519828	-0.33363	-0.067457637	-6.745763707	-1.27601	0.201951	0.393003	METFORMIN HYDROCHLORIDE
6.534026	-0.36238	-0.067459303	-6.745930265	-1.27604	0.20194	0.393003	DYDROGESTERONE
6.49693	-0.28009	-0.067462984	-6.746298447	-1.47266	0.140842	0.309092	Elacridar (GF120918)
6.528917	-0.42724	-0.067495325	-6.749532513	-1.49568	0.134737	0.299579	Scoparone
6.49693	-0.28322	-0.067601136	-6.760113616	-1.85294	0.063891	0.178463	GLUCONOLACTONE
6.49693	-0.28591	-0.067737588	-6.773758799	-1.44462	0.148566	0.320545	ALRESTATIN
6.482874	-0.32271	-0.067766388	-6.77663882	-5.30947	1.10E-07	1.34E-06	CARBINOXAMINE MALEATE
6.49276	-0.2718	-0.067801904	-6.780190445	-1.48006	0.138857	0.305738	Lonidamine
6.453318	-0.26456	-0.067826554	-6.782655433	-1.50302	0.132833	0.297379	Carbendazim
6.514548	-0.32294	-0.06787324	-6.787324019	-1.28387	0.199186	0.390441	OXTRIPHYLLINE
6.494155	-0.27457	-0.068128973	-6.812897256	-1.4872	0.136962	0.302504	Rilpivirine
6.50515	-0.29931	-0.06823123	-6.823123034	-1.87021	0.061455	0.173173	CYSTEAMINE HYDROCHLORIDE
6.469822	-0.22622	-0.068276423	-6.82764229	-1.49042	0.136114	0.301361	Bosentan
6.506505	-0.30534	-0.068359176	-6.835917649	-1.45787	0.144876	0.31486	FLUNISOLIDE
6.519828	-0.32559	-0.06836141	-6.836140985	-1.49227	0.135627	0.300741	Sofosbuvir (PSI-7977, GS-7977)
6.501059	-0.29563	-0.068479671	-6.847967125	-1.29534	0.195201	0.385286	PHENOTHIAZINE
6.537819	-0.44607	-0.068516858	-6.851685773	-1.15249	0.249119	0.449116	BENZYL NICOTINATE
6.536558	-0.44369	-0.068676614	-6.867661368	-1.52186	0.128044	0.290561	Methandrostenolone
6.503791	-0.29983	-0.068732722	-6.873272153	-1.46584	0.142692	0.312349	ERYTHROMYCIN ESTOLATE
6.4843	-0.32575	-0.06874973	-6.874973004	-5.38651	7.18E-08	8.93E-07	CRESOL
6.463893	-0.21859	-0.068993814	-6.899381358	-1.89111	0.05861	0.167737	NALED
6.525045	-0.3442	-0.069174336	-6.917433568	-1.30848	0.190709	0.378755	TRICHLORFON
6.4843	-0.32575	-0.069279892	-6.92798922	-5.42805	5.70E-08	7.11E-07	DISULFIRAM
6.522444	-0.41301	-0.069309504	-6.930950423	-1.16582	0.243685	0.442593	Acrivastine
6.509203	-0.31212	-0.069315496	-6.93154965	-1.31116	0.189805	0.377399	BENAZEPRIL HYDROCHLORIDE
6.480007	-0.32199	-0.06933288	-6.933288007	-1.5364	0.12444	0.284561	Doripenem
6.509203	-0.30448	-0.069335921	-6.933592071	-1.51355	0.130141	0.294441	Lafutidine
6.539076	-0.38238	-0.069353702	-6.935370177	-2.66508	0.007697	0.03466	PAROXYPROPIONE
6.525045	-0.3442	-0.0694131	-6.941309993	-1.313	0.189183	0.37655	FIUMAZENIL
6.506505	-0.30196	-0.069514722	-6.95147223	-1.90539	0.05673	0.164099	FEXOFENADINE HYDROCHLORIDE
6.522444	-0.41301	-0.069568135	-6.95681349	-1.17017	0.241931	0.440656	Tolcapone
6.511883	-0.32731	-0.069689228	-6.968922752	-2.67797	0.007407	0.03365	ORNITHINE HYDROCHLORIDE
6.517196	-0.32036	-0.069722744	-6.972274366	-1.52199	0.128011	0.290561	LCZ696
6.509203	-0.31082	-0.069867444	-6.986744351	-1.49004	0.136214	0.30139	HYDROXYZINE HYDROCHLORIDE
6.517196	-0.33807	-0.069930068	-6.993006817	-2.68823	0.007205	0.032907	PANTETHINE
6.463893	-0.28709	-0.070048708	-7.004870824	-1.17826	0.238694	0.435825	Caspofungin Acetate
6.450249	-0.25775	-0.070219896	-7.02198963	-1.18114	0.237548	0.434684	RUFLOXACIN HYDROCHLORIDE
6.469822	-0.30008	-0.070260576	-7.026057614	-1.55696	0.11948	0.277432	Drostanolone Propionate
6.522444	-0.3487	-0.070282788	-7.028278844	-2.70078	0.006918	0.031823	OXINIACIC ACID
6.50515	-0.31368	-0.070335567	-7.033556707	-2.70281	0.006876	0.031658	NIFLUMIC ACID
6.522444	-0.41301	-0.070456929	-7.045692919	-1.18512	0.235968	0.433324	Goserelin Acetate
6.503791	-0.29372	-0.070552669	-7.055266906	-1.54011	0.123534	0.284062	Alfacalcidol
6.489958	-0.33781	-0.070608669	-7.060868906	-5.53216	3.16E-08	4.08E-07	EPINEPHRINE BITARTRATE
6.509203	-0.30723	-0.070723167	-7.072316675	-1.93851	0.052561	0.155826	TRANDOLAPRIL
6.523746	-0.35134	-0.070819121	-7.081912123	-2.72139	0.006501	0.030231	DIMETRIDAZOLE
6.511883	-0.3903	-0.070900635	-7.090063467	-1.19259	0.233031	0.429797	PITAVASTATIN CALCIUM
6.507856	-0.3046	-0.070903051	-7.090305078	-1.94344	0.051963	0.154638	LEVOFLOXACIN
6.49693	-0.28727	-0.070980961	-7.098096055	-1.34266	0.179383	0.362947	RALOXIFENE HYDROCHLORIDE
6.523746	-0.33338	-0.071036399	-7.103639907	-1.55067	0.120982	0.279903	Nelarabine
6.507856	-0.31916	-0.071060518	-7.106051838	-2.73067	0.006321	0.029608	EDARAVONE
6.521138	-0.4105	-0.071159811	-7.115981095	-1.57689	0.114822	0.270801	Diclofenac
6.513218	-0.31246	-0.071190911	-7.119091143	-1.55404	0.120175	0.278667	Tepotinib (EMD 1214063)
6.534026	-0.37215	-0.071227947	-7.122794679	-2.7371	0.006198	0.029089	BROXYQUINOLINE
6.50515	-0.30259	-0.07123278	-7.12327797	-1.51916	0.128723	0.291749	SPIRONOLACTONE

6.494155	-0.27457	-0.071328736	-7.132873592	-1.55705	0.119459	0.277432	Zibotentan (ZD4054)
6.451786	-0.1949	-0.071458803	-7.145880264	-1.95868	0.050151	0.150511	PROPOFOL
6.515874	-0.32029	-0.071808904	-7.180890398	-1.96827	0.049037	0.148385	ISOXICAM
6.4843	-0.25851	-0.07184211	-7.184210977	-1.96918	0.048932	0.148332	LOXAPINE SUCCINATE
6.503791	-0.37317	-0.071847607	-7.184760726	-1.59213	0.111356	0.265183	Nifuratel
6.535294	-0.44096	-0.072053606	-7.205360622	-1.59669	0.110334	0.263239	Andrographolide
6.482874	-0.32271	-0.072084488	-7.208448816	-5.64779	1.63E-08	2.20E-07	EUCALYPTOL
6.534026	-0.37215	-0.072113319	-7.211331918	-2.77112	0.005586	0.026831	EXALAMIDE
6.488551	-0.33481	-0.072117816	-7.211781591	-5.6504	1.60E-08	2.17E-07	NAFCILLIN SODIUM
6.501059	-0.29429	-0.072199292	-7.219929185	-1.53977	0.123617	0.284074	TUBOCURARINE CHLORIDE PENTAHYDRATE
6.472756	-0.30615	-0.072201324	-7.220132405	-1.21447	0.22457	0.420094	Biapenem
6.491362	-0.3408	-0.072218081	-7.221808063	-5.65826	1.53E-08	2.08E-07	POLYMYXIN B SULFATE
6.534026	-0.37215	-0.072296261	-7.229626071	-2.77815	0.005467	0.026381	TOLPERISONE HYDROCHLORIDE
6.5302	-0.42969	-0.072342404	-7.234240382	-1.21684	0.223665	0.419014	BENZTROPINE MESYLATE
6.495544	-0.28446	-0.072485099	-7.248509935	-1.37111	0.170341	0.352239	CEFONICID SODIUM
6.501059	-0.29429	-0.072487541	-7.248754086	-1.54592	0.122125	0.282038	XYLAZINE
6.4843	-0.27146	-0.072601707	-7.260170714	-2.78989	0.005273	0.025516	CHLOROPYRAMINE HYDROCHLORIDE
6.519828	-0.3434	-0.072678832	-7.267883179	-2.79285	0.005225	0.025307	REBAMIPIDE
6.522444	-0.41331	-0.07268644	-7.268643986	-1.61072	0.107242	0.256698	Chondroitine sulfate
6.494155	-0.35217	-0.072743619	-7.274361883	-1.22359	0.221108	0.416969	Meptazinol HCl
6.498311	-0.36111	-0.072817122	-7.28171225	-1.22482	0.220641	0.416397	Palonosetron HCl
6.534026	-0.37215	-0.072943693	-7.294369296	-2.80303	0.005062	0.024615	TOLFENAMIC ACID
6.501059	-0.29429	-0.073221435	-7.322143481	-1.56157	0.11839	0.275901	KETOTIFEN FUMARATE
6.503791	-0.31093	-0.073283847	-7.328384706	-2.8161	0.004861	0.023839	CAPRYLIDENE
6.4843	-0.32575	-0.073303994	-7.330399419	-5.74334	9.28E-09	1.30E-07	ACETYLCYSTEINE
6.515874	-0.32029	-0.073478183	-7.347818298	-2.01403	0.044007	0.137303	EDOXUDINE
6.534026	-0.43792	-0.073652175	-7.365217545	-1.23887	0.215393	0.410577	BEXAROTENE
6.491362	-0.3408	-0.07365371	-7.365370992	-5.77074	7.89E-09	1.11E-07	MECLOFENAMATE SODIUM
6.519828	-0.32559	-0.073791699	-7.379169913	-1.61081	0.107221	0.256698	Rasagiline Mesylate
6.546543	-0.3975	-0.073851585	-7.385158516	-2.83792	0.004541	0.022616	DIOSMIN
6.52763	-0.3592	-0.074034015	-7.403401519	-2.84493	0.004442	0.022232	SUCRALOSE
6.506505	-0.30196	-0.074124117	-7.412411663	-2.03173	0.042181	0.132902	SUVOREXANT
6.481443	-0.32483	-0.0741666	-7.416659975	-1.24752	0.212206	0.406926	Tiludronate Disodium
6.489958	-0.33781	-0.074225821	-7.422582105	-5.81556	6.04E-09	8.57E-08	DIFLUNISAL
6.50515	-0.29642	-0.074246229	-7.424622873	-1.62073	0.105075	0.252978	Efavirenz
6.501059	-0.28829	-0.074285419	-7.428541941	-1.62159	0.104891	0.252768	Licofelone
6.481443	-0.25292	-0.074325236	-7.432523577	-2.03724	0.041626	0.131557	AMLODIPINE BESYLATE
6.517196	-0.32036	-0.074734304	-7.473430435	-1.63139	0.102808	0.249497	TAK-700 (Orteronel)
6.507856	-0.30808	-0.074753272	-7.475327228	-1.59424	0.110883	0.264179	TRANYLCYPROMINE SULFATE
6.488551	-0.26891	-0.074781926	-7.478192589	-1.59485	0.110746	0.263976	PYRIMETHAMINE
6.501059	-0.29429	-0.074792298	-7.479229846	-1.59507	0.110697	0.263976	URSODIOL
6.515874	-0.32563	-0.074911549	-7.49115488	-1.41701	0.15648	0.332716	TROCLOSENE SODIUM
6.506505	-0.29912	-0.074918314	-7.491831411	-1.63541	0.101964	0.248243	Olmutinib (HM61713, BI 1482694)
6.475671	-0.24422	-0.075086823	-7.508682309	-1.42032	0.155513	0.331762	DENATONIUM BENZOATE
6.494155	-0.28165	-0.075094191	-7.509419139	-1.42046	0.155473	0.331762	TEICOPLANIN [A(2-1) shown]
6.517196	-0.40172	-0.075095894	-7.509589421	-1.26315	0.206534	0.398437	Lithocholic acid
6.515874	-0.32563	-0.075143222	-7.514322249	-1.42139	0.155203	0.331376	CAPOBENIC ACID
6.485721	-0.32878	-0.075145494	-7.514549365	-5.88762	3.92E-09	5.62E-08	NORETHINDRONE ACETATE
6.482874	-0.32271	-0.075156392	-7.515639186	-5.88847	3.90E-09	5.61E-08	PHENACEMIDE
6.49693	-0.35266	-0.075364186	-7.536418552	-5.90475	3.53E-09	5.09E-08	DEXCHLORPHENIRAMINE MALEATE
6.485721	-0.26129	-0.075444653	-7.544465324	-2.06793	0.038647	0.124284	IOXILAN
6.515874	-0.39918	-0.075540919	-7.554091878	-1.67397	0.094136	0.234882	Carbasalate Calcium
6.518514	-0.32545	-0.07555947	-7.555946972	-2.07107	0.038352	0.12342	VORINOSTAT
6.471292	-0.30324	-0.075559628	-7.555962809	-1.67439	0.094055	0.234793	Spiculisporic Acid
6.506505	-0.37901	-0.075564779	-7.556477895	-1.6745	0.094032	0.234793	Fosfomycin Calcium
6.501059	-0.29429	-0.075929065	-7.592906531	-1.61931	0.10538	0.253307	BETAHISTINE DIHYDROCHLORIDE
6.471292	-0.30324	-0.076019706	-7.601970568	-1.68458	0.09207	0.23143	Hydroxy Camptothecine
6.534026	-0.35381	-0.076130255	-7.613025466	-1.66186	0.096541	0.239249	Dienogest
6.482874	-0.32791	-0.076274851	-7.627485056	-1.28299	0.199497	0.390752	Tiopronin
6.49693	-0.28727	-0.076289548	-7.628954792	-1.44307	0.148999	0.321176	METOLAZONE
6.517196	-0.33807	-0.076291663	-7.629166289	-2.93168	0.003371	0.017631	XYLOSE
6.509203	-0.30448	-0.076379291	-7.637929143	-1.6673	0.095455	0.237594	Agomelatine
6.526339	-0.34682	-0.076430981	-7.643098097	-1.44575	0.148247	0.320264	GUANFACINE HYDROCHLORIDE
6.502427	-0.29707	-0.076733934	-7.673393391	-1.63648	0.101739	0.247933	ROXARSONE
6.444045	-0.24461	-0.076800802	-7.680080178	-1.70189	0.088776	0.225653	Paeonol
6.481443	-0.25591	-0.07685975	-7.685974971	-1.45386	0.145985	0.316579	HYDROCORTISONE VALERATE
6.495544	-0.28051	-0.077036597	-7.703659655	-2.11156	0.034724	0.113602	CANDICIDIN
6.489958	-0.28292	-0.077070552	-7.707055198	-2.96162	0.00306	0.016187	CHLORAZANIL HYDROCHLORIDE
6.509203	-0.30723	-0.077115607	-7.711560685	-2.11373	0.034539	0.113067	QUIPAZINE MALEATE
6.50515	-0.3761	-0.077121925	-7.712192529	-1.70901	0.08745	0.22376	Fosphenytoin·Na Pentahydrate
6.462398	-0.22711	-0.077220409	-7.722040911	-2.96737	0.003004	0.015936	GATIFLOXACIN
6.488551	-0.33481	-0.077451397	-7.745139723	-6.06829	1.29E-09	1.99E-08	APOMORPHINE HYDROCHLORIDE
6.517196	-0.32287	-0.077583647	-7.758364661	-2.12656	0.033457	0.110444	BERGENIN
6.535294	-0.35633	-0.077823994	-7.782399416	-1.69883	0.08935	0.226584	Pazopanib
6.539076	-0.37261	-0.077988397	-7.798839651	-1.47521	0.140156	0.307851	DIATRIZOIC ACID
6.531479	-0.43275	-0.078006763	-7.800676262	-1.72861	0.083878	0.216829	Ifenprodil Tartrate
6.52763	-0.3592	-0.07807408	-7.807407962	-3.00018	0.002698	0.014557	RONNEL
6.491362	-0.3408	-0.078123471	-7.812347131	-6.12094	9.30E-10	1.47E-08	CARBAMAZEPINE
6.468347	-0.22329	-0.078232008	-7.823200798	-1.70774	0.087684	0.223912	Nystatin (Fungicidin)
6.4843	-0.27146	-0.078286505	-7.828650467	-3.00834	0.002627	0.014232	CARBIMAZOLE
6.481443	-0.26567	-0.078472959	-7.847295915	-3.01551	0.002566	0.013974	CHINIOFON
6.513218	-0.39317	-0.078511421	-7.851142124	-1.32061	0.186633	0.372538	Clindamycin Phosphate
6.49693	-0.28591	-0.078612607	-7.861260664	-1.67654	0.093632	0.234094	BUDESONIDE
6.510545	-0.31354	-0.078661563	-7.866156269	-1.67759	0.093427	0.233797	ZALCITABINE
6.49693	-0.35266	-0.078772421	-7.877242145	-6.17179	6.75E-10	1.08E-08	CHLOROXYLENOL
6.511883	-0.3098	-0.078796501	-7.879650102	-1.72006	0.085421	0.21978	Fostamatinib (R788)
6.511883	-0.39059	-0.079197762	-7.919776179	-1.75501	0.079258	0.208961	Sulfogaiacol
6.5302	-0.3644	-0.079229841	-7.9229841	-3.04459	0.00233	0.012814	THIODIGLYCOL
6.480007	-0.253	-0.079276683	-7.927668278	-1.49958	0.133724	0.298459	DIMETHYLSULFONE
6.536558	-0.36751	-0.079303531	-7.930353097	-1.50009	0.133592	0.298295	SULISOBENZONE
6.522444	-0.3487	-0.079551325	-7.955132543	-3.05694	0.002236	0.012377	INOSINE
6.49693	-0.29703	-0.079603929	-7.96039288	-3.05897	0.002221	0.01232	SECURININE
6.517196	-0.32287	-0.07968865	-7.968864985	-2.18425	0.028944	0.09886	TAURINE
6.540329	-0.45147	-0.079897835	-7.989783478	-1.34393	0.178972	0.362476	BENDAMUSTINE HYDROCHLORIDE
6.523746	-0.41581	-0.079939111	-7.993911108	-1.34462	0.178748	0.362207	Dolasetron
6.4843	-0.25499	-0.079970801	-7.997080127	-1.7457	0.080864	0.211667	Cerdulatinib (PRT062070, PRT2070)
6.489958	-0.33781	-0.08010876	-8.010876022	-6.27649	3.46E-10	5.65E-09	HYDROXYPROGESTERONE CAPROATE
6.466868	-0.29349	-0.08022604	-8.022603969	-1.34945	0.177194	0.360155	Ramelteon
6.444045	-0.17499	-0.080328104	-8.032810387	-1.7535	0.079517	0.209319	Embelin
6.523746	-0.33338	-0.08037282	-8.037282028	-1.75447	0.079349	0.209094	VX-809 (Lumacaftor)
6.513218	-0.33002	-0.080426303	-8.042630272	-3.09057	0.001998	0.011327	BITOSCANATE
6.480007	-0.32175	-0.080589388	-8.058938843	-1.35556	0.17524	0.358511	QUININE HDROCHLORIDE
6.429752	-0.21385	-0.080841508	-8.08415079	-1.79143	0.073224	0.196858	Bupropion
6.489958	-0.26958	-0.081014712	-8.101471161	-2.2206	0.026378	0.091937	AZILSARTAN MEDOXOMIL
6.478566	-0.25985	-0.08120755	-8.120755046	-3.12059	0.001805	0.010395	PRASTERONE
6.445604	-0.24796	-0.081320756	-8.13207563	-1.80205	0.071537	0.19359	AN-2690
6.525045	-0.4186	-0.081366392	-8.136639195	-1.36863	0.171116	0.352986	Cysteamine HCl
6.495544	-0.28051	-0.081423285	-8.142328541	-2.2318	0.025628	0.089751	CEFTRIAXONE SODIUM TRIHYDRATE
6.488551	-0.2703	-0.081617349	-8.161734877	-1.54385	0.122624	0.282681	FELBINAC
6.498311	-0.28871	-0.081617835	-8.161783483	-1.74064	0.081747	0.213032	SALICYLAMIDE
6.459392	-0.27741	-0.08164779	-8.164778995	-1.37336	0.16964	0.3515	Cinepazide maleate
6.50515	-0.30391	-0.08183355	-8.183354953	-1.54794	0.121636	0.281036	AMINOPTERIN
6.501059	-0.29429	-0.081843794	-8.184379447	-1.74545	0.080906	0.21167	CEFOXITIN SODIUM
6.503791	-0.29983	-0.081867373	-8.18673733	-1.74596	0.080818	0.211667	TINIDAZOLE
6.491362	-0.27599	-0.081885959	-8.188595872	-1.54893	0.121397	0.280738	TAPENTADOL HYDROCHLORIDE
6.506505	-0.30666	-0.08199118	-8.199118028	-1.55093	0.12092	0.279886	ETHOPABATE
6.515874	-0.31773	-0.082059915	-8.205991546	-1.7913	0.073245	0.196858	Dolutegravir (GSK1349572)
6.528917	-0.3458	-0.082064834	-8.206483369	-2.24939	0.024488	0.086587	PIROCTONE OLAMINE
6.513218	-0.39346	-0.082116466	-8.211646643	-1.81968	0.068807	0.188796	Aconitine
6.481443	-0.25449	-0.082160647	-8.216064662	-1.75221	0.079737	0.209734	PYRANTEL PAMOATE
6.509203	-0.31082	-0.082241274	-8.224127352	-1.75393	0.079442	0.209231	CISPLATIN
6.50515	-0.29931	-0.082318721	-8.231872064	-2.25634	0.024049	0.085271	VILAZODONE HYDROCHLORIDE
6.507856	-0.30808	-0.082667465	-8.266746501	-1.76302	0.077897	0.205584	ROPINIROLE HYDROCHLORIDE
6.487138	-0.26744	-0.082871699	-8.287169947	-1.56758	0.116979	0.273742	IMEXON
6.545307	-0.395	-0.082912495	-8.291249546	-3.18611	0.001442	0.008565	DROPROPIZINE
6.518514	-0.32545	-0.082928281	-8.29282812	-2.27305	0.023023	0.082674	BETAMETHASONE 17,21-DIPROPIONATE
6.488551	-0.2703	-0.082997906	-8.299790625	-1.56997	0.116423	0.273182	OXYBUTYNIN CHLORIDE
6.498311	-0.3556	-0.083180228	-8.318022796	-6.51714	7.17E-11	1.21E-09	ESTRADIOL VALERATE
6.531479	-0.35723	-0.08331235	-8.331234953	-1.57592	0.115045	0.271193	GUANETHIDINE MONOSULFATE
6.451786	-0.26106	-0.083641646	-8.36416463	-1.4069	0.159457	0.33664	Losmapimod (GW856553X)
6.513218	-0.33002	-0.083757746	-8.375774648	-3.21859	0.001288	0.007751	INDINAVIR SULFATE

6.501059	-0.29563	-0.08395652	-8.395651967	-1.5881	0.112263	0.266106	ETHANOLAMINE OLEATE
6.532754	-0.35981	-0.083983065	-8.398306487	-1.5886	0.11215	0.266083	DILOXANIDE FUROATE
6.525045	-0.33596	-0.084007032	-8.400703175	-1.83381	0.066683	0.183854	Obeticholic Acid
6.502427	-0.29101	-0.084048793	-8.404879326	-1.83472	0.066548	0.183768	Batimastat (BB-94)
6.49693	-0.28009	-0.084082446	-8.408244566	-1.83545	0.066439	0.183768	Iniparib (BSI-201)
6.460898	-0.20848	-0.084342611	-8.434261087	-1.84113	0.065602	0.182249	Vemurafenib (PLX4032, RG7204)
6.503791	-0.29372	-0.084378713	-8.437871269	-1.84192	0.065487	0.182027	Ibrutinib (PCI-32765)
6.514548	-0.33271	-0.084560987	-8.456098678	-3.24945	0.001156	0.00706	OXELAIDIN CITRATE
6.509203	-0.31082	-0.084685016	-8.46850165	-1.80605	0.070911	0.192811	URACIL
6.509203	-0.32189	-0.084709369	-8.470936867	-3.25515	0.001133	0.006941	ADELMIDROL
6.49693	-0.35841	-0.084753525	-8.47535252	-1.87812	0.060365	0.171061	Azasetron hydrochloride
6.507856	-0.38192	-0.084758319	-8.475831902	-1.87823	0.06035	0.171061	Laetrile
6.513218	-0.31896	-0.08482933	-8.482933045	-1.80913	0.070431	0.192172	TRIHEXYPHENIDYL HYDROCHLORIDE
6.499687	-0.35854	-0.084980797	-8.498079698	-6.65821	2.77E-11	4.81E-10	TESTOSTERONE PROPIONATE
6.513218	-0.31509	-0.085021469	-8.50214695	-2.33043	0.019784	0.073882	ALFUZOSIN HYDROCHLORIDE
6.431364	-0.21732	-0.085181398	-8.518139756	-1.8876	0.059079	0.168893	Cefotetan Disodium
6.494155	-0.27457	-0.085183977	-8.5183977	-1.8595	0.062957	0.176624	Epacadostat (INCB024360)
6.517196	-0.32036	-0.085426531	-8.542653066	-1.86479	0.062211	0.174914	Argatroban
6.502427	-0.2984	-0.085556894	-8.555689371	-1.61837	0.105582	0.253616	CEFPODOXIME PROXETIL
6.485721	-0.32878	-0.085646102	-8.564610244	-6.71034	1.94E-11	3.42E-10	OXYPHENBUTAZONE
6.475671	-0.31266	-0.085646899	-8.564689932	-1.89792	0.057707	0.166082	Naloxegol oxalate
6.503791	-0.31093	-0.085698822	-8.569882227	-3.29318	0.000991	0.006133	HYDROXYPROGESTERONE
6.515874	-0.32563	-0.085725788	-8.572578843	-1.62157	0.104896	0.252768	FINASTERIDE
6.534026	-0.35381	-0.085992878	-8.599287757	-1.87715	0.060497	0.171224	Tamibarotene
6.513218	-0.31896	-0.086022988	-8.602298841	-1.83458	0.066568	0.183768	AMPROLIUM
6.503791	-0.29665	-0.086182905	-8.618290453	-2.36226	0.018164	0.068939	LINAGLIPTIN
6.459392	-0.21125	-0.086199377	-8.619937658	-1.63053	0.10299	0.249673	BUSPIRONE HYDROCHLORIDE
6.502427	-0.2984	-0.0862298	-8.622980036	-1.6311	0.102869	0.249497	MESALAMINE
6.507856	-0.30808	-0.086266773	-8.626677266	-1.83978	0.0658	0.182699	ACECLIDINE
6.468347	-0.22939	-0.08627832	-8.627832016	-1.63202	0.102675	0.249382	BENZYDAMINE HYDROCHLORIDE
6.477121	-0.24072	-0.086381211	-8.638121118	-1.88563	0.059345	0.169028	Erlotinib
6.52763	-0.34943	-0.086404737	-8.640473677	-1.63441	0.102173	0.248629	ALTRENOGEST
6.480007	-0.26276	-0.086455991	-8.645599074	-3.32227	0.000893	0.005623	CYTISINE
6.506505	-0.30666	-0.086460608	-8.646060784	-1.63547	0.101951	0.248243	SYMCLOSENE
6.487138	-0.26063	-0.086554169	-8.655416861	-1.88941	0.058837	0.168294	Oclacitinib
6.49693	-0.35266	-0.086735704	-8.673570437	-6.79571	1.08E-11	1.93E-10	DIPYRIDAMOLE
6.50515	-0.29642	-0.086751785	-8.67517846	-1.89372	0.058262	0.167116	Cabozantinib (XL184, BMS-907351)
6.525045	-0.4186	-0.086936353	-8.693635261	-1.46232	0.143654	0.313918	EDOXABAN TOSYLATE HYDRATE
6.499687	-0.28557	-0.086994504	-8.699450439	-1.89902	0.057562	0.165758	Safinamide Mesylate
6.488551	-0.26683	-0.087514893	-8.75148935	-2.39877	0.01645	0.064343	RAMELTEON
6.49693	-0.35266	-0.087706116	-8.7706116	-6.87174	6.34E-12	1.16E-10	DACARBAZINE
6.49276	-0.34378	-0.087771233	-8.777123283	-6.87684	6.12E-12	1.12E-10	DANAZOL
6.50515	-0.31368	-0.087823561	-8.782356142	-3.37482	0.000739	0.004715	ERYTHROMYCIN STEARATE
6.448706	-0.25464	-0.087891144	-8.789114415	-1.94765	0.051457	0.153621	Selexipag(NS-304)
6.537819	-0.44607	-0.087913672	-8.791367174	-1.47876	0.139205	0.306156	CYSTINE
6.463893	-0.28732	-0.087939341	-8.793934063	-1.94872	0.051329	0.153418	Ceftizoxim·Na
6.485721	-0.32878	-0.088026052	-8.802605239	-6.89681	5.32E-12	9.87E-11	ADENOSINE
6.480007	-0.24646	-0.088291779	-8.829177902	-1.92734	0.053938	0.15849	Bitopertin
6.509203	-0.31082	-0.088419064	-8.841906377	-1.88568	0.059338	0.169028	JOSAMYCIN
6.50515	-0.29642	-0.088442665	-8.844266546	-1.93063	0.053529	0.157691	Ulixertinib (BVD-523, VRT752271)
6.513218	-0.31896	-0.088458505	-8.845850521	-1.88652	0.059224	0.169011	FLUMETHASONE
6.519828	-0.32559	-0.088688705	-8.86887047	-1.936	0.052867	0.156234	Telaprevir (VX-950)
6.489958	-0.27177	-0.088729485	-8.87294847	-1.8923	0.058451	0.167375	SULFADIMETHOXINE
6.50515	-0.30259	-0.088977875	-8.897787483	-1.8976	0.057749	0.166109	CAPTOPRIL
6.488551	-0.33481	-0.089296086	-8.929608575	-6.99631	2.63E-12	4.96E-11	FLUOCINONIDE
6.495544	-0.28446	-0.089392984	-8.939298425	-1.69094	0.090849	0.229589	SITAGLIPTIN PHOSPHATE
6.509203	-0.30723	-0.089435545	-8.943554516	-2.45142	0.01423	0.057321	CELECOXIB
6.488551	-0.33481	-0.089469349	-8.946934915	-7.00989	2.39E-12	4.56E-11	HYDROCHLOROTHIAZIDE
6.482874	-0.25215	-0.089902661	-8.990266118	-1.9625	0.049704	0.149609	MEK162 (ARRY-162, ARRY-438162)
6.466868	-0.22491	-0.089920114	-8.992011364	-1.9177	0.05515	0.160344	QUININE
6.49276	-0.2718	-0.089957672	-8.995767212	-1.9637	0.049565	0.149277	Nilotinib (AMN-107)
6.501059	-0.30539	-0.08999553	-8.999553049	-3.45829	0.000544	0.00355	CHLORALOSE
6.514548	-0.3151	-0.090184287	-9.018428693	-1.96865	0.048993	0.148342	Serotonin HCl
6.495544	-0.2831	-0.09029502	-9.029501975	-1.92569	0.054143	0.15877	NEFOPAM
6.532754	-0.35128	-0.090320007	-9.032000678	-1.97161	0.048654	0.147839	Tazarotene
6.457882	-0.20249	-0.090517466	-9.051746572	-1.97592	0.048164	0.146784	Macitentan
6.509203	-0.30448	-0.090576055	-9.0576055	-1.9772	0.048019	0.146518	Zosuquidar (LY335979) 3HCl
6.4843	-0.32575	-0.090675013	-9.067501265	-7.10435	1.21E-12	2.35E-11	NORGESTREL
6.495544	-0.34971	-0.090688922	-9.068892191	-7.10544	1.20E-12	2.34E-11	CARBOPLATIN
6.541579	-0.45416	-0.090737434	-9.073743389	-1.52625	0.126947	0.288741	LINACLOTIDE (1 mg/ml)
6.526339	-0.33853	-0.090774965	-9.077496465	-1.98154	0.04753	0.145761	Rotigotine
6.521138	-0.34605	-0.090884732	-9.088473242	-3.49246	0.000479	0.003175	METITEPINE MESYLATE
6.494155	-0.34675	-0.090916537	-9.091653747	-7.12327	1.05E-12	2.07E-11	PIPERACILLIN SODIUM
6.392697	-0.07295	-0.090982184	-9.098218391	-1.98607	0.047026	0.144608	Posaconazole
6.518514	-0.32971	-0.09110054	-9.110054008	-1.94287	0.052032	0.154638	TOLNAFTATE
6.528917	-0.42693	-0.091408936	-9.140893608	-1.53755	0.124159	0.284341	FLURANDRENOLIDE
6.478566	-0.25985	-0.091428467	-9.142846693	-3.51335	0.000442	0.002958	PROCODAZOLE
6.536558	-0.44336	-0.09146989	-9.146989011	-1.53857	0.123908	0.284234	ANASTROZOLE
6.482874	-0.32271	-0.091469894	-9.146989378	-7.16663	7.69E-13	1.52E-11	MOXALACTAM DISODIUM
6.523746	-0.33338	-0.091487689	-9.148768885	-1.9971	0.045814	0.141819	Moxonidine
6.396199	-0.07991	-0.091609007	-9.160900702	-1.99975	0.045527	0.141187	LDK378
6.503791	-0.36728	-0.091863308	-9.186330754	-7.19745	6.14E-13	1.23E-11	ESTRIOL
6.519828	-0.32802	-0.092012194	-9.201219399	-2.52204	0.011668	0.048846	ALLYLESTRENOL
6.482874	-0.25215	-0.092036257	-9.203625676	-2.00908	0.044529	0.138427	Otenabant (CP-945598) HCl
6.487138	-0.33708	-0.092042847	-9.204284683	-1.54821	0.121571	0.281014	Testosterone Enanthate
6.49693	-0.35266	-0.092096018	-9.2096018	-7.21569	5.37E-13	1.08E-11	AMINOCAPROIC ACID HYDROCHLORIDE
6.536558	-0.44369	-0.092301648	-9.230164759	-2.04538	0.040817	0.129722	Astaxanthin
6.494155	-0.28165	-0.092326249	-9.23262487	-1.74642	0.080738	0.211663	ISRADIPINE
6.463893	-0.21444	-0.092394193	-9.239419328	-2.01689	0.043707	0.136534	Vismodegib (GDC-0449)
6.522444	-0.41301	-0.092591065	-9.259106488	-1.55743	0.119368	0.277432	RIVASTIGMINE TARTRATE
6.515874	-0.32435	-0.092781084	-9.278108374	-1.97871	0.047849	0.146347	TRIMEPRAZINE TARTRATE
6.50515	-0.29931	-0.093179012	-9.317901207	-2.55402	0.010649	0.045701	DEXLANSOPRAZOLE
6.499687	-0.35854	-0.093215559	-9.321555858	-7.3034	2.81E-13	5.79E-12	INDAPAMIDE
6.49276	-0.27882	-0.093258624	-9.325862413	-1.76406	0.077722	0.205334	PENTAGASTRIN
6.498311	-0.29983	-0.093966205	-9.396620525	-3.61087	0.000305	0.002109	CRESOPIRINE
6.49276	-0.34378	-0.093983887	-9.398388714	-7.3636	1.79E-13	3.80E-12	DEMECLOCYCLINE HYDROCHLORIDE
6.513218	-0.33002	-0.094079227	-9.407922746	-3.61521	0.0003	0.002085	PHENOTHRIN
6.52763	-0.42416	-0.094196023	-9.419602326	-1.58443	0.113096	0.267832	DESOGESTREL
6.518514	-0.32298	-0.094224866	-9.422486622	-2.05685	0.0397	0.126725	Deoxycholic acid
6.498311	-0.28871	-0.094313932	-9.431393177	-2.0114	0.044283	0.137829	PYRILAMINE MALEATE
6.502427	-0.36438	-0.094412365	-9.441236499	-7.39717	1.39E-13	3.00E-12	OXYTETRACYCLINE
6.521138	-0.34605	-0.094429403	-9.442940253	-3.62867	0.000285	0.001998	ISAXONINE
6.499687	-0.28862	-0.094692846	-9.469284633	-2.59552	0.009445	0.041191	AMINOHIPPURIC ACID
6.491362	-0.3408	-0.094776253	-9.477625292	-7.42568	1.12E-13	2.43E-12	BETHANECHOL CHLORIDE
6.509203	-0.30723	-0.094999177	-9.499917726	-2.60391	0.009217	0.040332	TETROQUINONE
6.514548	-0.32294	-0.095040878	-9.5040878	-1.79777	0.072213	0.194904	ACESULFAME POTASSIUM
6.502427	-0.29398	-0.095128772	-9.51287723	-2.60747	0.009122	0.040053	CARVEDILOL
6.502427	-0.36438	-0.095264482	-9.526448247	-7.46393	8.39E-14	1.84E-12	METHYLPREDNISOLONE
6.522444	-0.33314	-0.095310931	-9.531093081	-2.61246	0.008989	0.039541	FOLIC ACID
6.537819	-0.36135	-0.095315699	-9.531569895	-2.08066	0.037465	0.120859	Tivozanib (AV-951)
6.514548	-0.32166	-0.09535213	-9.535212962	-2.03354	0.041998	0.132488	PROBUCOL
6.549003	-0.39271	-0.095715829	-9.571582939	-1.81054	0.070213	0.191932	DESONIDE
6.55145	-0.39767	-0.095944877	-9.594487735	-1.81487	0.069544	0.190613	SOLIFENACIN SUCCINATE
6.507856	-0.3046	-0.096180974	-9.618097448	-2.63631	0.008381	0.037164	CETIRIZINE HYDROCHLORIDE
6.498311	-0.28592	-0.09644983	-9.644982977	-2.64368	0.008201	0.036481	FLUOXETINE HYDROCHLORIDE
6.522444	-0.3487	-0.09659278	-9.659278019	-3.7118	0.000206	0.001478	TENOFOVIR
6.514548	-0.33271	-0.096695138	-9.669513764	-3.71574	0.000203	0.001459	MOXISYLYTE HYDROCHLORIDE
6.502427	-0.36438	-0.096734318	-9.673431843	-7.57909	3.49E-14	7.83E-13	CEPHRADINE
6.521138	-0.33058	-0.096831085	-9.683108534	-2.65413	0.007951	0.035587	OXYPHENCYCLIMINE HYDROCHLORIDE
6.465383	-0.28544	-0.097331735	-9.733173537	-7.6259	2.42E-14	5.51E-13	DORAMECTIN
6.52763	-0.42416	-0.097470965	-9.74709651	-1.63952	0.101106	0.247094	METHOXYVONE
6.515874	-0.39888	-0.097895804	-9.789580372	-1.64666	0.099628	0.245237	Oxytocin (Syntocinon)
6.502427	-0.2984	-0.09806295	-9.806294952	-1.85493	0.063606	0.178151	SODIUM MONOFLUOROPHOSPHATE
6.502427	-0.30816	-0.098217795	-9.821779504	-3.77425	0.00016	0.001177	BENZOCLIDINE
6.515874	-0.32435	-0.098282168	-9.828216833	-2.09603	0.03608	0.116979	PROADIFEN HYDROCHLORIDE
6.480007	-0.26276	-0.098358942	-9.835894171	-3.77967	0.000157	0.001157	CARGLUMIC ACID
6.546543	-0.46517	-0.098418039	-9.841803925	-2.18092	0.029189	0.099433	Patchouli alcohol
6.489958	-0.27177	-0.098575005	-9.857500522	-2.10228	0.035529	0.115645	LOMUSTINE
6.531479	-0.35082	-0.098842272	-9.884227162	-2.70925	0.006743	0.031161	TRIFLUPROMAZINE HYDROCHLORIDE
6.510545	-0.31484	-0.098851266	-9.885126578	-1.86985	0.061505	0.173173	NIACINAMIDE
6.539076	-0.4491	-0.098860269	-9.886026945	-2.19072	0.028472	0.097574	17-Beta-Estradiol-3,17-Dipropionate

6.437751	-0.23087	-0.098905397	-9.890539718	-1.66364	0.096184	0.238595	Vildagliptin (LAF-237)
6.534026	-0.43824	-0.099074827	-9.907482719	-2.19548	0.028129	0.096724	Menatetrenone
6.542825	-0.37129	-0.099167232	-9.916723175	-2.16474	0.030408	0.102696	Dofetilide
6.509203	-0.30723	-0.09944239	-9.944239016	-2.7257	0.006416	0.02992	FENOFIBRIC ACID
6.488551	-0.26683	-0.099578233	-9.957823264	-2.72943	0.006344	0.029661	CITALOPRAM HYDROBROMIDE
6.457882	-0.2082	-0.099739871	-9.973987137	-1.88666	0.059207	0.169011	METHYLPHENIDATE HYDROCHLORIDE
6.513218	-0.31246	-0.099921353	-9.992135254	-2.1812	0.029168	0.099429	Maraviroc
6.50515	-0.29642	-0.100135017	-10.01350172	-2.18587	0.028825	0.098588	Teneligliptin hydrobromide
6.480007	-0.3166	-0.100340066	-10.03400663	-7.8616	3.77E-15	9.07E-14	METHSCOPOLAMINE BROMIDE
6.485721	-0.26129	-0.100451163	-10.0451163	-2.75335	0.005899	0.02793	FLUCONAZOLE
6.506505	-0.30666	-0.100530097	-10.05300973	-1.9016	0.057223	0.16506	MEGLUTOL
6.567026	-0.43898	-0.100669702	-10.06697018	-3.86847	0.00011	0.000825	CEPHALOSPORIN C Zn
6.509203	-0.31212	-0.100673232	-10.0673232	-1.90431	0.05687	0.164319	TIBOLONE
6.482874	-0.32271	-0.101014044	-10.10140437	-7.91441	2.44E-15	5.96E-14	NIFEDIPINE
6.502427	-0.29398	-0.101158371	-10.11583711	-2.77274	0.005559	0.026724	MEFLOQUINE HYDROCHLORIDE
6.511883	-0.3098	-0.101323981	-10.13239811	-2.21182	0.026979	0.093713	Potassium Iodide
6.525045	-0.33596	-0.101416988	-10.14169883	-2.21385	0.026839	0.09329	Iloperidone
6.502427	-0.2984	-0.101569294	-10.15692938	-1.92126	0.054699	0.15968	PROTIRELIN
6.545307	-0.395	-0.10168386	-10.16838598	-3.90744	9.33E-05	0.000714	IPRIFLAVONE
6.546543	-0.38773	-0.101883356	-10.18833565	-1.9272	0.053955	0.15849	PRULIFLOXACIN
6.439333	-0.23447	-0.101959494	-10.19594939	-2.2594	0.023858	0.084791	Cefoxitin·Na
6.537819	-0.37007	-0.101975758	-10.19757578	-1.92895	0.053737	0.158123	ATRACURIUM BESYLATE
6.507856	-0.30939	-0.102151328	-10.21513285	-1.93227	0.053326	0.157276	CYPROHEPTADINE HYDROCHLORIDE
6.503791	-0.36728	-0.102304345	-10.23043447	-8.0155	1.11E-15	2.75E-14	FLUMETHAZONE PIVALATE
6.515874	-0.39918	-0.102311662	-10.23116617	-2.2672	0.023378	0.083469	Flurandrenolide
6.494155	-0.34675	-0.102333075	-10.23330747	-8.01775	1.11E-15	2.75E-14	NITROFURANTOIN
6.485721	-0.25781	-0.102493135	-10.24931346	-2.23734	0.025264	0.088719	17-AAG (Tanespimycin)
6.525045	-0.33823	-0.102670224	-10.26702243	-2.81418	0.00489	0.02396	TRAZODONE HYDROCHLORIDE
6.480007	-0.253	-0.102708467	-10.27084672	-1.94281	0.052039	0.154638	NIZATIDINE
6.519828	-0.32802	-0.102842262	-10.28422624	-2.81889	0.004819	0.023679	IRBESARTAN
6.534026	-0.35381	-0.102954441	-10.29544412	-2.24741	0.024614	0.086852	Amuvatinib (MP-470)
6.468347	-0.29667	-0.103478592	-10.34785916	-1.74057	0.081759	0.213032	Rofecoxib
6.468347	-0.29667	-0.103642875	-10.36428745	-1.74333	0.081276	0.212096	Azilsartan
6.503791	-0.29665	-0.103680751	-10.36807507	-2.84187	0.004485	0.022381	BROMPHENIRAMINE MALEATE
6.552668	-0.47835	-0.103954299	-10.39542985	-2.30361	0.021245	0.078023	Betulin
6.528917	-0.42693	-0.104085933	-10.40859334	-1.75078	0.079983	0.210223	USNIC ACID
6.431364	-0.21732	-0.104108477	-10.41084769	-2.30702	0.021054	0.077439	Lorcaserin
6.514548	-0.32166	-0.104134877	-10.41348772	-2.22085	0.026361	0.091937	VALPROATE SODIUM
6.498311	-0.3556	-0.104293082	-10.4293082	-8.17132	2.22E-16	5.60E-15	DIFLUBENZURON
6.501059	-0.28829	-0.104305381	-10.43053813	-2.2769	0.022792	0.082117	CX-4945 (Silmitasertib)
6.502427	-0.36438	-0.104312578	-10.43125784	-8.17285	2.22E-16	5.60E-15	NITROFURAZONE
6.471292	-0.30324	-0.104353995	-10.43539953	-2.31246	0.020752	0.076605	Darunavir
6.49693	-0.29703	-0.104368674	-10.43686737	-4.01061	6.06E-05	0.00048	meta-CRESYL ACETATE
6.477121	-0.24072	-0.104538141	-10.45381414	-2.28198	0.02249	0.081436	Alcaftadine
6.509203	-0.30448	-0.104569229	-10.45692286	-2.28266	0.02245	0.081406	Alibendol
6.514548	-0.33271	-0.104605874	-10.46058736	-4.01972	5.83E-05	0.000464	EFAROXAN HYDROCHLORIDE
6.518514	-0.32298	-0.10476893	-10.47689295	-2.28702	0.022195	0.080823	Filgotinib (GLPG0634)
6.531479	-0.43244	-0.104998948	-10.49989483	-1.76614	0.077372	0.204594	Cabozantinib malate (XL184)
6.471292	-0.30324	-0.105060655	-10.5060655	-2.32812	0.019906	0.074283	Clavulanate Potassium
6.457882	-0.27438	-0.105243151	-10.52431511	-2.33217	0.019692	0.073593	ARRY380
6.471292	-0.30324	-0.10525968	-10.52596804	-2.33253	0.019673	0.073575	Cidofovir
6.525045	-0.35397	-0.10598192	-10.59819205	-4.0726	4.65E-05	0.00038	BETAMIPRON
6.499687	-0.35854	-0.106019149	-10.60191493	-8.30656	0	5.60E-15	DEXTROMETHORPHAN HYDROBROMIDE
6.531479	-0.34875	-0.106636834	-10.66368336	-2.3278	0.019923	0.074294	Ozanimod (RPC1063)
6.515874	-0.32029	-0.106722149	-10.67221486	-2.92524	0.003442	0.017909	NATEGLINIDE
6.491362	-0.26902	-0.107555137	-10.75551367	-2.34784	0.018883	0.07114	Lenvatinib (E7080)
6.485721	-0.25781	-0.107825695	-10.78256952	-2.35375	0.018585	0.070203	Albendazole Oxide
6.502427	-0.29398	-0.107968881	-10.79688815	-2.95941	0.003082	0.016269	CHLOROGUANIDE HYDROCHLORIDE
6.49693	-0.28591	-0.108016639	-10.80166391	-2.30363	0.021243	0.078023	TENYLIDONE
6.518514	-0.32971	-0.108325421	-10.83254212	-2.31022	0.020876	0.076951	CEFOTETAN
6.525045	-0.33596	-0.108466839	-10.84668386	-2.36774	0.017897	0.068281	Etoricoxib
6.457882	-0.27417	-0.108559705	-10.85597047	-1.82603	0.067845	0.186456	TRIAMCINOLONE DIACETATE
6.503791	-0.29983	-0.108637376	-10.86373765	-2.31687	0.020511	0.075933	CHLOROQUINE DIPHOSPHATE
6.495544	-0.35516	-0.108675268	-10.86752678	-1.82798	0.067553	0.185852	Tirofiban·HCl
6.5302	-0.3462	-0.109174019	-10.91740185	-2.38318	0.017164	0.066312	Naratriptan
6.525045	-0.34296	-0.109403843	-10.94038427	-2.33322	0.019637	0.073494	STREPTOZOSIN
6.477121	-0.31045	-0.109410453	-10.94104533	-8.57226	0	5.60E-15	ETHIONAMIDE
6.511883	-0.3098	-0.109710644	-10.97106436	-2.39489	0.016625	0.064928	EX 527 (Selisistat)
6.50515	-0.30259	-0.110009358	-11.00093577	-2.34613	0.018969	0.071309	SACCHARIN
6.518514	-0.32545	-0.110613192	-11.06131922	-3.03189	0.00243	0.013294	TRIFLUMURON
6.431364	-0.21732	-0.110836373	-11.08363729	-2.45611	0.014045	0.056756	Mizolastine
6.541579	-0.36882	-0.110890529	-11.08905288	-2.42065	0.015493	0.061206	Melatonin
6.565848	-0.43659	-0.111138087	-11.11380866	-4.27074	1.95E-05	0.000173	PROTOPORPHYRIN IX
6.518514	-0.32971	-0.111159539	-11.11595393	-2.37066	0.017756	0.067997	ATENOLOL
6.469822	-0.24214	-0.111223535	-11.12235351	-4.27402	1.92E-05	0.000171	IDRAMANTONE
6.488551	-0.26891	-0.111667609	-11.1667609	-2.3815	0.017242	0.066477	SULFAMONOMETHOXINE
6.540329	-0.36633	-0.112092368	-11.20923683	-2.44689	0.01441	0.057956	Fluoxymesterone
6.519828	-0.32802	-0.112118741	-11.21187412	-3.07316	0.002118	0.011865	ARMODAFINIL
6.50515	-0.30391	-0.11275125	-11.27512498	-2.13278	0.032943	0.108959	ZOLMITRIPTAN
6.49276	-0.2718	-0.112763292	-11.27632924	-2.46153	0.013835	0.055994	Pralatrexate
6.50515	-0.37018	-0.11287652	-11.28765197	-8.84383	0	5.60E-15	FLUOCINOLONE ACETONIDE
6.49693	-0.28009	-0.113249074	-11.32490737	-2.47214	0.013431	0.054576	Povidone iodine
6.513218	-0.32025	-0.113296553	-11.32965529	-2.14309	0.032106	0.107089	PHENYLETHYL ALCOHOL
6.553883	-0.40259	-0.113357623	-11.33576235	-2.14425	0.032013	0.106989	PREDNISOLONE SODIUM PHOSPHATE
6.518514	-0.32298	-0.113365328	-11.33653284	-2.47467	0.013336	0.054276	Malotilate
6.521138	-0.33504	-0.113815332	-11.3815332	-2.4273	0.015212	0.06032	UREA
6.510545	-0.30714	-0.113999602	-11.39996017	-2.48852	0.012828	0.052753	Dapoxetine HCl
6.503791	-0.30116	-0.114189752	-11.41897517	-2.15999	0.030774	0.103796	RISPERIDONE
6.549003	-0.39271	-0.114535251	-11.4535251	-2.16652	0.030271	0.102371	EXEMESTANE
6.511883	-0.31755	-0.114733717	-11.47337174	-2.17028	0.029986	0.101607	SOTALOL HYDROCHLORIDE
6.507856	-0.30939	-0.114961128	-11.49611283	-2.17458	0.029662	0.100776	PHYSOSTIGMINE SULFATE
6.519828	-0.32802	-0.115163669	-11.51636695	-3.15662	0.001596	0.009318	CARVEDILOL PHOSPHATE
6.488551	-0.26344	-0.115218747	-11.52187473	-2.51513	0.011899	0.049611	Cilengitide
6.501059	-0.28829	-0.115396311	-11.53963108	-2.51901	0.011769	0.049188	Motolimod (VTX-2337)
6.519828	-0.33238	-0.115833016	-11.58330159	-2.47033	0.013499	0.054765	OCTODRINE
6.447158	-0.18118	-0.115858853	-11.58588525	-2.5291	0.011435	0.048189	Pexidartinib (PLX3397)
6.521138	-0.33504	-0.116086357	-11.60863569	-2.47573	0.013296	0.054201	DIFLOXACIN HYDROCHLORIDE
6.503791	-0.29372	-0.116247085	-11.62470849	-2.53758	0.011162	0.047244	Pranlukast
6.499687	-0.35854	-0.116264977	-11.62649769	-9.10931	0	5.60E-15	ATROPINE SULFATE
6.465383	-0.2903	-0.116459963	-11.64599631	-1.95892	0.050122	0.150511	Bepotastine Besilate
6.518514	-0.32971	-0.116631704	-11.66317043	-2.48736	0.012869	0.05284	DOXIFLURIDINE
6.466868	-0.29372	-0.116859389	-11.68593887	-2.58958	0.009609	0.04173	Bendazac L-lysine
6.539076	-0.36384	-0.117450558	-11.74505582	-2.56385	0.010352	0.044464	Quizartinib (AC220)
6.528917	-0.36181	-0.117512655	-11.75126552	-4.5157	6.31E-06	6.04E-05	GUANIDINE HYDROCHLORIDE
6.49276	-0.2718	-0.11752765	-11.75276501	-2.56553	0.010302	0.044286	Zafirlukast
6.509203	-0.31212	-0.117589166	-11.7589166	-2.22429	0.026129	0.091256	ETHYL PARABEN
6.517196	-0.32287	-0.118087677	-11.80876768	-3.23677	0.001209	0.007334	TRIMETOZINE
6.429752	-0.21385	-0.118478932	-11.84789316	-2.62547	0.008653	0.038193	Kitasamycin
6.537819	-0.36322	-0.118544243	-11.85442426	-3.24928	0.001157	0.00706	ISOFLUPREDONE ACETATE
6.50515	-0.29642	-0.119425716	-11.94257158	-2.60697	0.009135	0.040077	Gimeracil
6.49276	-0.27882	-0.119471504	-11.9471504	-2.25989	0.023828	0.084791	CAPTAMINE
6.521138	-0.4102	-0.119836	-11.98359999	-2.01571	0.043831	0.136837	Reboxetine mesylate
6.506505	-0.37307	-0.120068016	-12.00680162	-9.40728	0	5.60E-15	ISOSORBIDE DINITRATE
6.472756	-0.30615	-0.120175429	-12.01754287	-2.02142	0.043237	0.135561	Fosaprepitant dimeglumine salt
6.522444	-0.33314	-0.120324091	-12.03240907	-3.29807	0.000974	0.006042	ROSUVASTATIN CALCIUM
6.499687	-0.29285	-0.120396075	-12.03960746	-2.27738	0.022763	0.082117	RABEPRAZOLE SODIUM
6.499687	-0.28557	-0.12039918	-12.03991801	-2.62822	0.008583	0.037984	Pilaralisib (XL147)
6.539076	-0.44878	-0.12049308	-12.04930801	-2.02676	0.042687	0.134249	Bimatoprost
6.4843	-0.26169	-0.120699125	-12.06991247	-2.28312	0.022424	0.081367	ZALEPLON
6.499687	-0.30262	-0.1217856	-12.17856002	-4.67989	2.87E-06	2.89E-05	MEPIROXOL
6.531479	-0.34875	-0.122175126	-12.21751262	-2.66698	0.007654	0.034617	Daclatasvir (BMS-790052)
6.503791	-0.36728	-0.122289582	-12.2289582	-9.58134	0	5.60E-15	AMINOSALICYLATE SODIUM
6.52763	-0.3411	-0.122319483	-12.23194835	-2.67013	0.007582	0.03437	Elvitegravir (GS-9137, JTK-303)
6.535294	-0.35633	-0.122800686	-12.28006859	-2.68064	0.007348	0.033472	Tofacitinib (CP-690550) Citrate
6.4843	-0.27146	-0.123241311	-12.32413107	-4.73583	2.18E-06	2.25E-05	ENOXOLONE
6.514548	-0.3151	-0.123534595	-12.35345953	-2.69666	0.007004	0.032161	Aloxistatin
6.550228	-0.4731	-0.123714789	-12.37147893	-2.74149	0.006116	0.028729	Fraxetin
6.521138	-0.33058	-0.12375389	-12.37538898	-3.39208	0.000694	0.00445	TELMISARTAN
6.475671	-0.23784	-0.123945291	-12.39452912	-2.70562	0.006818	0.031447	Rifapentine
6.539076	-0.38238	-0.124128634	-12.41286336	-4.76993	1.84E-06	1.92E-05	COUMARIN
6.526339	-0.34559	-0.124339919	-12.43399191	-2.65176	0.008007	0.035747	TRIAMCINOLONE

6.50515	-0.30391	-0.124622211	-12.46222108	-2.35732	0.018407	0.069759	TERPENE HYDRATE
6.499687	-0.35854	-0.124693242	-12.46932425	-9.76966	0	5.60E-15	GUAIFENESIN
6.544068	-0.45951	-0.124719624	-12.47196244	-2.09785	0.035918	0.116688	HYDROQUININE HYDROBROMIDE HYDRATE
6.507856	-0.37594	-0.12532919	-12.53291901	-9.81949	0	5.60E-15	MECAMYLAMINE HYDROCHLORIDE
6.522444	-0.40703	-0.125450023	-12.54500226	-9.82896	0	5.60E-15	CHLORPROMAZINE HYDROCHLORIDE
6.517196	-0.39585	-0.125554439	-12.55544394	-9.83714	0	5.60E-15	FLUDROCORTISONE ACETATE
6.519828	-0.33238	-0.125596483	-12.55964828	-2.67855	0.007394	0.033621	DAPAGLIFLOZIN
6.511883	-0.38453	-0.125630488	-12.5630488	-9.8431	0	5.60E-15	PHENINDIONE
6.501059	-0.29563	-0.125731251	-12.57312508	-2.3783	0.017393	0.066955	DOXAZOSIN MESYLATE
6.507856	-0.3018	-0.125878283	-12.58782834	-2.74782	0.005999	0.028233	Evacetrapib (LY2484595)
6.502427	-0.29101	-0.12610863	-12.61086299	-2.75285	0.005908	0.02793	Ciclesonide
6.478566	-0.2436	-0.126594775	-12.65947752	-2.76346	0.005719	0.027265	R788 (Fostamatinib) Disodium
6.456366	-0.19948	-0.126748934	-12.67489345	-2.76683	0.00566	0.027035	Suprofen
6.466868	-0.29372	-0.126924606	-12.69246059	-2.81262	0.004914	0.024053	Gamma-Oryzanol
6.531479	-0.35082	-0.127151138	-12.71511384	-3.48519	0.000492	0.003241	ACETYLSEROTONIN
6.518514	-0.32298	-0.127448929	-12.74489289	-2.78211	0.005401	0.026087	Amprenavir
6.507856	-0.37594	-0.127740952	-12.77409517	-10.0085	0	5.60E-15	AMANTADINE HYDROCHLORIDE
6.513218	-0.31896	-0.127880028	-12.7880028	-2.72725	0.006386	0.029807	GALANTAMINE
6.536558	-0.35884	-0.128050245	-12.80502454	-2.79523	0.005186	0.025169	Uridine
6.509203	-0.32189	-0.128070184	-12.80701835	-4.92139	8.59E-07	9.52E-06	OROTIC ACID
6.434569	-0.22422	-0.128264804	-12.82648036	-2.84232	0.004479	0.022381	Icotinib
6.528917	-0.3458	-0.128777894	-12.87778938	-3.52978	0.000416	0.002809	LEFLUNOMIDE
6.534026	-0.35381	-0.129539052	-12.95390522	-2.82773	0.004688	0.023168	Balofloxacin
6.510545	-0.30714	-0.129796601	-12.97966013	-2.83335	0.004606	0.022897	Vidofludimus
6.495544	-0.27733	-0.129951636	-12.99516361	-2.83674	0.004558	0.022678	Tedizolid Phosphate
6.469822	-0.22622	-0.130439621	-13.04396208	-2.84739	0.004408	0.022104	Anidulafungin (LY303366)
6.519828	-0.33363	-0.130507477	-13.05074773	-2.46865	0.013562	0.05498	ORBIFLOXACIN
6.518514	-0.39865	-0.130580904	-13.05809039	-10.231	0	5.60E-15	DEXAMETHASONE ACETATE
6.503791	-0.29372	-0.131145839	-13.11458393	-2.86281	0.004199	0.021202	Diphenidol HCl
6.534026	-0.37215	-0.132303455	-13.23034551	-5.08407	3.69E-07	4.27E-06	DEFERIPRONE
6.518514	-0.32298	-0.132370561	-13.23705613	-2.88954	0.003858	0.019773	VX-222 (VCH-222, Lomibuvir)
6.540329	-0.36813	-0.132658708	-13.26587085	-3.63616	0.000277	0.001946	TEGASEROD MALEATE
6.475671	-0.23784	-0.132756694	-13.27566942	-2.89797	0.003756	0.019288	Ripasudil (K-115)
6.469822	-0.22622	-0.132840386	-13.28403864	-2.8998	0.003734	0.019214	LY2157299
6.513218	-0.38737	-0.133286722	-13.32867217	-10.443	0	5.60E-15	NOSCAPINE HYDROCHLORIDE
6.564666	-0.50417	-0.134511556	-13.45115563	-2.98075	0.002875	0.015352	Mesterolone
6.561101	-0.41721	-0.134751757	-13.47517566	-2.54893	0.010805	0.046257	LINDANE
6.502427	-0.29707	-0.135300167	-13.53001667	-2.8855	0.003908	0.019949	SULFAMETHOXYPYRIDAZINE
6.481443	-0.31966	-0.135572245	-13.55722449	-10.622	0	5.60E-15	METHOXSALEN
6.4843	-0.33098	-0.135811983	-13.5811983	-2.28443	0.022346	0.081237	Quinidine·HCl·H2O
6.507856	-0.37594	-0.136116268	-13.61162681	-10.6647	0	5.60E-15	BISACODYL
6.525045	-0.33596	-0.136348342	-13.63483417	-2.97637	0.002917	0.01554	CB1954
6.485721	-0.25781	-0.13660706	-13.66070596	-2.98202	0.002864	0.015304	5,5-Dimethyloxazolidine-2,4-dione
6.510545	-0.38167	-0.137105658	-13.71056577	-10.7422	0	5.60E-15	PINDOLOL
6.525045	-0.33596	-0.137484667	-13.74846668	-3.00118	0.002689	0.014525	Regorafenib (BAY 73-4506)
6.571709	-0.51932	-0.13818013	-13.81801299	-3.06204	0.002198	0.012221	Etilefrine hydrochloride
6.49276	-0.2718	-0.138411376	-13.84113764	-3.02141	0.002516	0.013734	Rifabutin
6.478566	-0.2473	-0.138723489	-13.87234893	-3.80239	0.000143	0.001066	ALOGLIPTIN BENZOATE
6.522444	-0.40703	-0.138776947	-13.87769467	-10.8731	0	5.60E-15	DEXAMETHASONE
6.456366	-0.27112	-0.139493017	-13.94930169	-3.09114	0.001994	0.011323	Capreomycin disulfate
6.52763	-0.34821	-0.14059754	-14.05975398	-2.99848	0.002713	0.014623	ISOTRETINON
6.482874	-0.25215	-0.141089991	-14.10899907	-3.07988	0.002071	0.011651	Taladegib (LY2940680)
6.507856	-0.3018	-0.141147195	-14.11471951	-3.08113	0.002062	0.011615	BIRB 796 (Doramapimod)
6.471292	-0.22914	-0.142684275	-14.26842753	-3.11468	0.001841	0.010558	Nalmefene HCl
6.518514	-0.40456	-0.142708996	-14.27089961	-2.40044	0.016375	0.064295	LY2228820
6.542825	-0.45684	-0.142746735	-14.27467349	-2.40108	0.016347	0.064233	GESTODENE
6.471292	-0.23535	-0.144031394	-14.40313941	-2.72446	0.006441	0.030005	THEOBROMINE
6.510545	-0.38167	-0.144539313	-14.45393131	-11.3246	0	5.60E-15	ISOPROTERENOL HYDROCHLORIDE
6.514548	-0.3151	-0.144630046	-14.46300457	-3.15716	0.001593	0.009312	Stanozolol
6.485721	-0.25781	-0.144810684	-14.48106841	-3.1611	0.001572	0.009227	Divalproex Sodium
6.526339	-0.41533	-0.145958894	-14.59588939	-11.4358	0	5.60E-15	HYDROCORTISONE HEMISUCCINATE
6.532754	-0.35331	-0.146402004	-14.64020043	-4.01286	6.00E-05	0.000476	BUPIVACAINE HYDROCHLORIDE
6.4843	-0.25499	-0.146461832	-14.64618317	-3.19714	0.001388	0.008263	Meprednisone
6.528917	-0.35204	-0.147128214	-14.71282137	-2.78304	0.005385	0.026036	TEMEFOS
6.532754	-0.35331	-0.14726406	-14.72640604	-4.03649	5.43E-05	0.000435	VALACYCLOVIR HYDROCHLORIDE
6.526339	-0.35659	-0.148502336	-14.8502336	-5.70655	1.15E-08	1.60E-07	ESCIN
6.503791	-0.36728	-0.148694457	-14.86944567	-11.6501	0	5.60E-15	GLUTATHIONE
6.52763	-0.34943	-0.149434862	-14.94348624	-2.82667	0.004703	0.023222	LACITOL
6.465383	-0.22339	-0.149616208	-14.96162076	-2.8301	0.004653	0.023042	ZILEUTON
6.444045	-0.24461	-0.14962932	-14.96293204	-3.31575	0.000914	0.0057	Benorilate
6.519828	-0.40145	-0.149832731	-14.9832731	-11.7393	0	5.60E-15	IMIPRAMINE HYDROCHLORIDE
6.454845	-0.19645	-0.150734977	-15.07349768	-3.29042	0.001	0.006186	EPZ-6438
6.55145	-0.38843	-0.151068642	-15.10686424	-3.29771	0.000975	0.006042	Aprepitant
6.475671	-0.23784	-0.151953463	-15.19534629	-3.31702	0.00091	0.005681	Alvelestat (AZD9668)
6.541579	-0.36882	-0.152059949	-15.20599488	-3.31935	0.000902	0.005641	Etonogestrel
6.523746	-0.34157	-0.152082236	-15.20822357	-2.87675	0.004018	0.020409	DECAMETHONIUM BROMIDE
6.550228	-0.38601	-0.152121034	-15.2121034	-3.32068	0.000898	0.005628	Ponesimod
6.535294	-0.35633	-0.152510145	-15.2510145	-3.32917	0.000871	0.005492	Rocilinostat (ACY-1215)
6.540329	-0.37398	-0.154244766	-15.42447661	-3.28953	0.001004	0.006198	RIFAMPIN
6.481443	-0.31966	-0.154262298	-15.42622975	-12.0864	0	5.60E-15	ETHINYL ESTRADIOL
6.509203	-0.32189	-0.154843149	-15.48431487	-5.95021	2.68E-09	3.92E-08	MOROXYDINE HYDROCHLORIDE
6.556303	-0.48582	-0.155465923	-15.54659228	-2.61502	0.008922	0.039279	Heparin sodium
6.5302	-0.35464	-0.155553927	-15.55539272	-2.94242	0.003257	0.017101	METHYLPREDNISOLONE SODIUM SUCCINATE
6.564666	-0.4342	-0.157317696	-15.73176964	-6.0453	1.49E-09	2.28E-08	FORMESTANE
6.558709	-0.40286	-0.158213213	-15.82132132	-3.45367	0.000553	0.003602	Dutasteride
6.545307	-0.46218	-0.158265116	-15.82651157	-2.66211	0.007765	0.034937	Radotinib
6.540329	-0.36813	-0.160465238	-16.04652378	-4.39833	1.09E-05	0.000101	BUPROPION
6.510545	-0.38771	-0.160572535	-16.05725354	-3.55825	0.000373	0.002535	Baricitinib (LY3009104, INCB028050)
6.535294	-0.43441	-0.162551897	-16.25518971	-12.7359	0	5.60E-15	MEGESTROL ACETATE
6.532754	-0.35128	-0.163542958	-16.35429583	-3.57001	0.000357	0.00243	Emricasan
6.536558	-0.4371	-0.163877631	-16.38776315	-12.8397	0	5.60E-15	PREDNISOLONE
6.525045	-0.4186	-0.164886773	-16.48867731	-2.77349	0.005546	0.026687	Etravirine (TMC125)
6.536558	-0.35884	-0.164887667	-16.48876667	-3.59936	0.000319	0.002192	Ivacaftor (VX-770)
6.49693	-0.28009	-0.165963988	-16.59639878	-3.62286	0.000291	0.002038	Ruxolitinib (INCB018424)
6.514548	-0.3902	-0.167195812	-16.71958117	-13.0997	0	5.60E-15	HOMATROPINE METHYLBROMIDE
6.557507	-0.40993	-0.16723619	-16.72361899	-3.1634	0.001559	0.009167	RIZATRIPTAN BENZOATE
6.49276	-0.27745	-0.16785817	-16.78581701	-3.57985	0.000344	0.002353	NICERGOLINE
6.485721	-0.33429	-0.168118292	-16.81182921	-3.72547	0.000195	0.001412	Odanacatib (MK-0822)
6.537819	-0.43979	-0.170454403	-17.04544029	-13.355	0	5.60E-15	HYDROCORTISONE PHOSPHATE TRIETHYLAMINE
6.535294	-0.43441	-0.172093347	-17.20933471	-13.4834	0	5.60E-15	DEXAMETHASONE SODIUM PHOSPHATE
6.541579	-0.4478	-0.172525902	-17.25259023	-13.5173	0	5.60E-15	PREDNISOLONE ACETATE
6.523746	-0.4098	-0.172567008	-17.25670083	-13.5206	0	5.60E-15	MAPROTILINE HYDROCHLORIDE
6.567026	-0.50889	-0.17714107	-17.71410703	-2.97961	0.002886	0.015393	Latanoprost
6.511883	-0.3098	-0.177277522	-17.72775219	-3.86982	0.000109	0.000821	Eltrombopag Olamine
6.514548	-0.31769	-0.180568427	-18.0568427	-4.94936	7.45E-07	8.32E-06	TACROLIMUS
6.555094	-0.40505	-0.180818529	-18.08185289	-3.42032	0.000625	0.004038	TROSPIUM CHLORIDE
6.478566	-0.3189	-0.182523488	-18.25234878	-4.04468	5.24E-05	0.000423	Bromfenac
6.526339	-0.41533	-0.182881239	-18.2881239	-14.3287	0	5.60E-15	IBUPROFEN
6.562293	-0.41962	-0.184359042	-18.43590417	-3.48729	0.000488	0.003223	EDITOL
6.499687	-0.28557	-0.185691622	-18.56916223	-4.0535	5.05E-05	0.000409	Emtricitabine
6.466868	-0.22035	-0.18651646	-18.65164598	-4.0715	4.67E-05	0.000381	Fluorometholone Acetate
6.550228	-0.38601	-0.188460433	-18.84604333	-4.11394	3.89E-05	0.000324	Sorafenib
6.459392	-0.20549	-0.189238167	-18.92381673	-4.13092	3.61E-05	0.000302	Sitafloxacin Hydrate
6.465383	-0.29053	-0.189503201	-18.95032005	-4.19935	2.68E-05	0.00023	Nimorazole
6.547775	-0.38113	-0.201337984	-20.13379837	-4.39504	1.11E-05	0.000103	Resminostat
6.488551	-0.26891	-0.203610549	-20.36105487	-4.34233	1.41E-05	0.000128	ROSIGLITAZONE MALEATE
6.480007	-0.24646	-0.204535029	-20.45350289	-4.46483	8.01E-06	7.57E-05	Ospemifene
6.535294	-0.43441	-0.204661079	-20.46610786	-16.0351	0	5.60E-15	ETHOPROPAZINE HYDROCHLORIDE
6.541579	-0.4478	-0.20489178	-20.48917799	-16.0532	0	5.60E-15	HISTAMINE DIHYDROCHLORIDE
6.555094	-0.4766	-0.205993945	-20.5993945	-16.1395	0	5.60E-15	MEDRYSONE
6.569374	-0.43396	-0.206209245	-20.62092449	-3.9006	9.60E-05	0.000731	OMEGA-3-ACID ESTERS (EPA shown)
6.597695	-0.49131	-0.207879663	-20.78796628	-3.9322	8.42E-05	0.00065	PROSCILLARIDIN A
6.499687	-0.28557	-0.214915809	-21.49158089	-4.69144	2.71E-06	2.75E-05	Ilomastat (GM6001, Galardin)
6.556303	-0.47918	-0.217585799	-21.75857988	-17.0478	0	5.60E-15	HYDROCORTISONE ACETATE
6.613842	-0.52401	-0.221930053	-22.19300529	-4.19797	2.69E-05	0.000231	MELENGESTROL ACETATE
6.598791	-0.5033	-0.245268619	-24.52686189	-9.42502	0	5.60E-15	PREDNICARBATE
6.576341	-0.4379	-0.251525326	-25.1525326	-5.49059	4.01E-08	5.14E-07	Sorafenib Tosylate
6.563481	-0.41234	-0.269388639	-26.93886391	-5.88053	4.09E-09	5.85E-08	Pexmetinib (ARRY-614)
6.605305	-0.58359	-0.313540813	-31.35408132	-24.5658	0	5.60E-15	MEDROXYPROGESTERONE ACETATE
6.642465	-0.67158	-0.322559132	-32.25591323	-7.14784	8.82E-13	1.74E-11	Chidamide
6.641474	-0.56733	-0.338015449	-33.80154489	-7.3786	1.60E-13	3.41E-12	Mocetinostat (MGCD0103)

6.643453	-0.57127	-0.390469608	-39.04696084	-8.52363	0	5.60E-15	Entinostat (MS-275)
6.913284	-1.13037	-0.916451089	-91.6451089	-17.3354	0	5.60E-15	FLUORESCEIN

Idarubicin (100%)
