# Supplementary material for: Repurposing anthelmintic agents to eradicate resistant leukemia
Source: Blood Cancer J. 2020 Jun 26;10(6):72. doi: 10.1038/s41408-020-0339-9 (PMC7320149; doi:10.1038/s41408-020-0339-9)
Supplement: Supplementary file 4 — supplementary table 3 [file 41408_2020_339_MOESM4_ESM.pdf]

Supplementary Table S3: Patient information of B- and T-ALL samples, and respective IC50 for each antihelmintic agent.

Sample	Risk Group	Clinical Stage	Age (years)	Sex	Class	Moxidectin (Log IC50)	Milbemycin (Log IC50)	Ivermectin (Log IC50)
B-R-03		Relapse	5.7	Female	B-ALL	3.09482038	3.101747074	3.200576927
B-R-10		Relapse	9.7	Male	B-ALL	2.99550357	3.159266331	3.183839037
B-SR-03	SR	Diagnosis	3.4	Female	B-ALL	2.914396052	3.039414119	3.25163822
B-R-01		Relapse	9.6	Female	B-ALL	3.029789471	3.16613397	3.258637283
B-SR-06	SR	Diagnosis	4	Female	B-ALL	2.711638538	3.026941628	3.182414652
B-VHR-10	VHR	Diagnosis	14.4	Female	B-ALL	2.760120785	3.027757205	3.218010043
T-VHR-04	VHR	Diagnosis	8	Male	T-ALL	3.208441356	3.288025535	3.38774566
B-VHR-12	VHR	Diagnosis	15.3	Female	B-ALL	3.314709693	3.254306332	3.448242413
B-VHR-29	VHR	Diagnosis	1	Female	B-ALL	3.136086097	3.270445908	3.73183042
B-SR-23	SR	Diagnosis	1.8	Female	B-ALL	2.996336518	N/A	N/A
B-R-23		Relapse	13	Female	B-ALL	3.075911761	N/A	N/A
B-R-30		Relapse	2	Male	B-ALL	3.080987047	N/A	N/A
B-R-29		Relapse	17	Female	B-ALL	3.054995862	N/A	N/A
T-non-HR-02	non HR	Diagnosis	7	Male	T-ALL	3.13893394	N/A	N/A
T-R-30		Relapse	17	Female	T-ALL	2.882524538	N/A	N/A
B-R-42		Relapse	6	Male	B-ALL	3.164947373	3.309417226	3.496929648
B-R-26		Relapse	10	Male	B-ALL	3.254064453	3.271841607	3.428296814
B-R-33		Relapse	10	Male	B-ALL	3.212187604	3.255031163	3.469232743
B-R-34		Relapse	11	Female	B-ALL	3.259593879	3.396373728	3.633872263
B-R-28		Relapse	3	Male	B-ALL	3.206556044	3.29841638	3.546542663
B-MR-02	MR	Diagnosis	2.5	Male	B-ALL	2.627365857	2.481729197	3.307709923
B-SR-25	SR	Diagnosis	1.2	Male	B-ALL	3.017867719	2.930082633	3.268577972
B-R-41		Relapse	12	Female	B-ALL	3.05804623	3.13289977	3.263399331
B-R-48		Relapse	14.5	Male	B-ALL	3.115277591	3.059941888	3.307923704
B-HR-28	HR	Diagnosis	12.4	Male	B-ALL	2.974235277	2.823409015	3.299725154
B-MR-03	MR	Diagnosis	3.5	Male	B-ALL	3.1430148	2.888628725	3.358125285
B-R-50		Relapse	19.4	Male	B-ALL	3.462547729	3.309417226	3.527501011
B-R-45		Relapse	2	Male	B-ALL	2.783331763	3.004751156	3.174350597
B-R-40		Relapse	15	Female	B-ALL	2.742489465	N/A	3.182699903
B-HR-29	HR	Diagnosis	12.8	Male	B-ALL	3.206015877	3.281033367	3.470263447
B-R-47		Relapse	2.5	Male	B-ALL	3.196728723	3.169674434	3.208441356
B-R-38		Relapse	1	Female	B-ALL	3.301464073	2.848681654	3.684845362
B-R-39		Relapse	16	Male	B-ALL	3.185542155	3.331832044	3.459241665
B-R-44		Relapse	16	Male	B-ALL	3.10720997	3.317645543	3.529301998
T-R-31		Relapse	3	Female	T-ALL	3.010723865	2.828466547	3.219322508
T-non-HR-01	non HR	Diagnosis	2.9	Male	T-ALL	3.142076461	3.07809415	3.42078062
B-R-35		Relapse	7	Male	B-ALL	3.220892249	3.397592434	3.396896449
B-R-36		Relapse	9 Months	Male	B-ALL	3.227886705	3.25791845	3.482444792
B-R-31		Relapse	2	Male	B-ALL	3.364363355	3.44294987	3.473632927
B-R-51		Relapse	7	Male	B-ALL	3.399154334	3.43679851	3.514282048
T-R-29		Relapse	10	Male	T-ALL	3.186391216	2.656481516	3.300378065
B-R-46		Relapse	6	Female	B-ALL	3.09725731	2.504334912	3.397418542
T-R-28		Relapse	13	Male	T-ALL	3.116275588	3.258637283	3.501333179
T-R-32		Relapse	17	Male	T-ALL	2.789651209	1.512150537	2.587374172
B-HR-30	HR	Diagnosis	2	Female	B-ALL	3.316389751	3.447623098	3.599773939
B-R-27		Relapse	17	Female	B-ALL	3.314077992	3.45591024	3.693639026
B-R-49		Relapse	6.4	Male	B-ALL	3.199206479	3.308350949	3.488550717
B-SR-26	SR	Diagnosis	3.2	Male	B-ALL	3.139879086	3.31196566	3.465828815
B-R-43		Relapse	4	Female	B-ALL	3.151982395	3.055378331	3.389520466
B-R-32		Relapse	6	Male	B-ALL	3.441223674	3.460145817	3.600101256
B-R-37		Relapse	12	Male	B-ALL	3.347720217	3.38147609	3.457276186
B-MR-04	MR	Diagnosis	3.1	Male	B-ALL	2.935507266	2.78554337	3.67669361
B-MR-05	MR	Diagnosis	3.1	Male	B-ALL	3.190891717	2.431363764	3.489536629
B-VHR-02	VHR	Diagnosis	12.8	Female	B-ALL	3.339650158	3.006466042	3.71941416
B-VHR-01	VHR	Diagnosis	14.1	Male	B-ALL	3.14	N/A	N/A

SR: standard risk
MR: medium risk
HR: high risk
non-HR: non high risk
VHR: very high risk

N/A: Not available
