# Supplementary material for: Repurposing anthelmintic agents to eradicate resistant leukemia
Source: Blood Cancer J. 2020 Jun 26;10(6):72. doi: 10.1038/s41408-020-0339-9 (PMC7320149; doi:10.1038/s41408-020-0339-9)
Supplement: Supplementary file 5 — supplementary table 4 [file 41408_2020_339_MOESM5_ESM.pdf]

Supplementary Table S4: Synergy values (Z-score) for combination of moxidectin and dexamethasone in B- and T-ALL samples

Sample	Z-score	Class
B-VHR-10	22.893	B-ALL
B-VHR-12	20.233	B-ALL
B-R-03	15.074	B-ALL
B-R-03	15.029	B-ALL
B-SR-26	12.39	B-ALL
T-R-28	12.02	T-ALL
B-R-32	11.85	B-ALL
T-VHR-04	9.36	T-ALL
B-R-37	9.1	B-ALL
B-R-49	6.22	B-ALL
T-R-32	5.82	T-ALL
T-R-32	4.72	B-ALL
B-MR-04	3.69	B-ALL
T-R-30	3.58	B-ALL
B-R-27	2.63	B-ALL
B-VHR-01	2.44	B-ALL
B-R-29	1.25	B-ALL
T-non-HR-02	0.23	T-ALL
B-SR-23	-2.81	B-ALL
