# Supplementary material for: Repurposing anthelmintic agents to eradicate resistant leukemia
Source: Blood Cancer J. 2020 Jun 26;10(6):72. doi: 10.1038/s41408-020-0339-9 (PMC7320149; doi:10.1038/s41408-020-0339-9)
Supplement: Supplementary file 6 — supplemental figures [file 41408_2020_339_MOESM6_ESM.pdf]

S1A

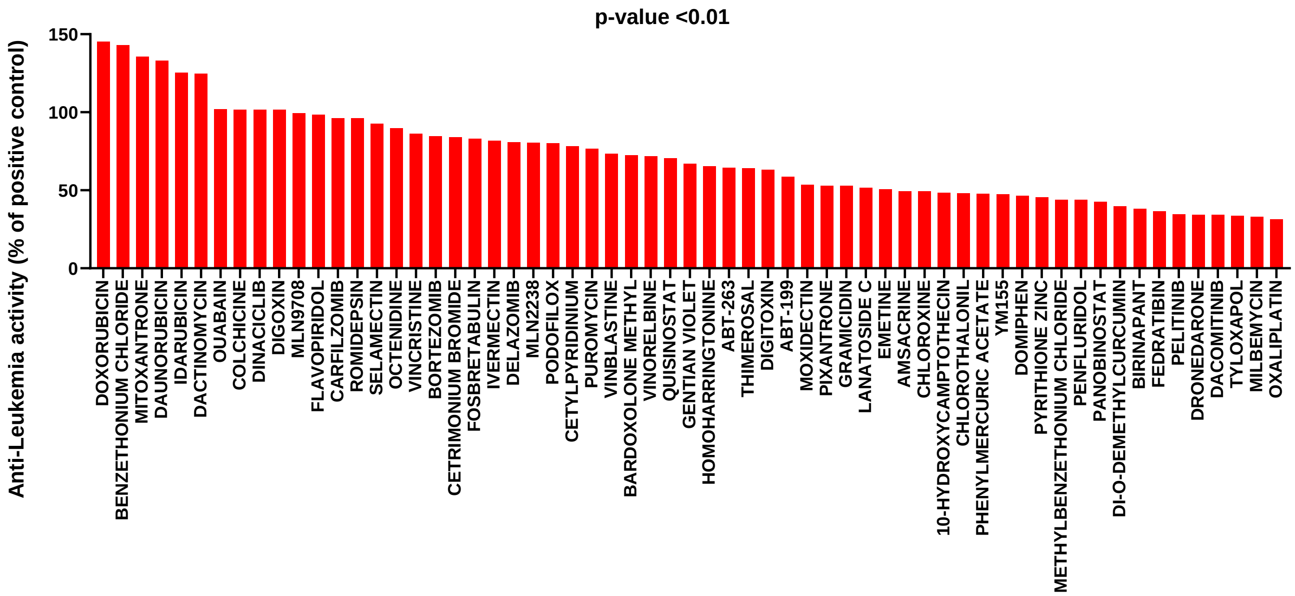

S1B

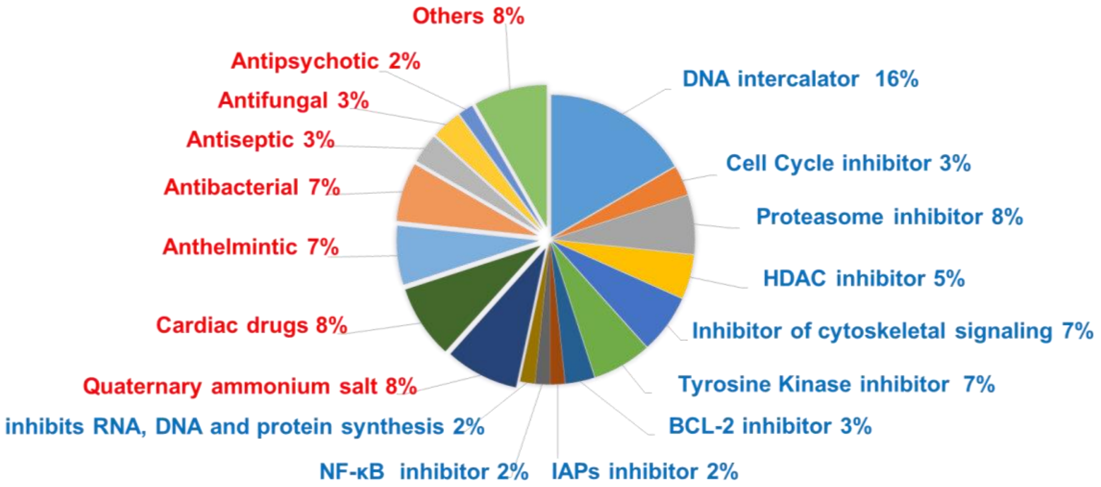

Identified compounds	61
Known anti-leukemia effect	33
New anti-leukemia effect	28

Figure S1: Results of *ex-vivo* drug repurposing screen. (**A**) Rank order of the compounds (n=61), with anti-leukemia activity > 30% of the positive control idarubicin and p-value<0.01. (**B**) Molecular and clinical classification of the compounds (n= 61) identified during the screen. The pie chart is depicting the distribution (in % of all) of the different drug families. The identified compounds included drugs already in use for anti-leukemia treatment (known anti-leukemia effect, n=33) and molecules with so far unappreciated anti-leukemia effect (n=28).

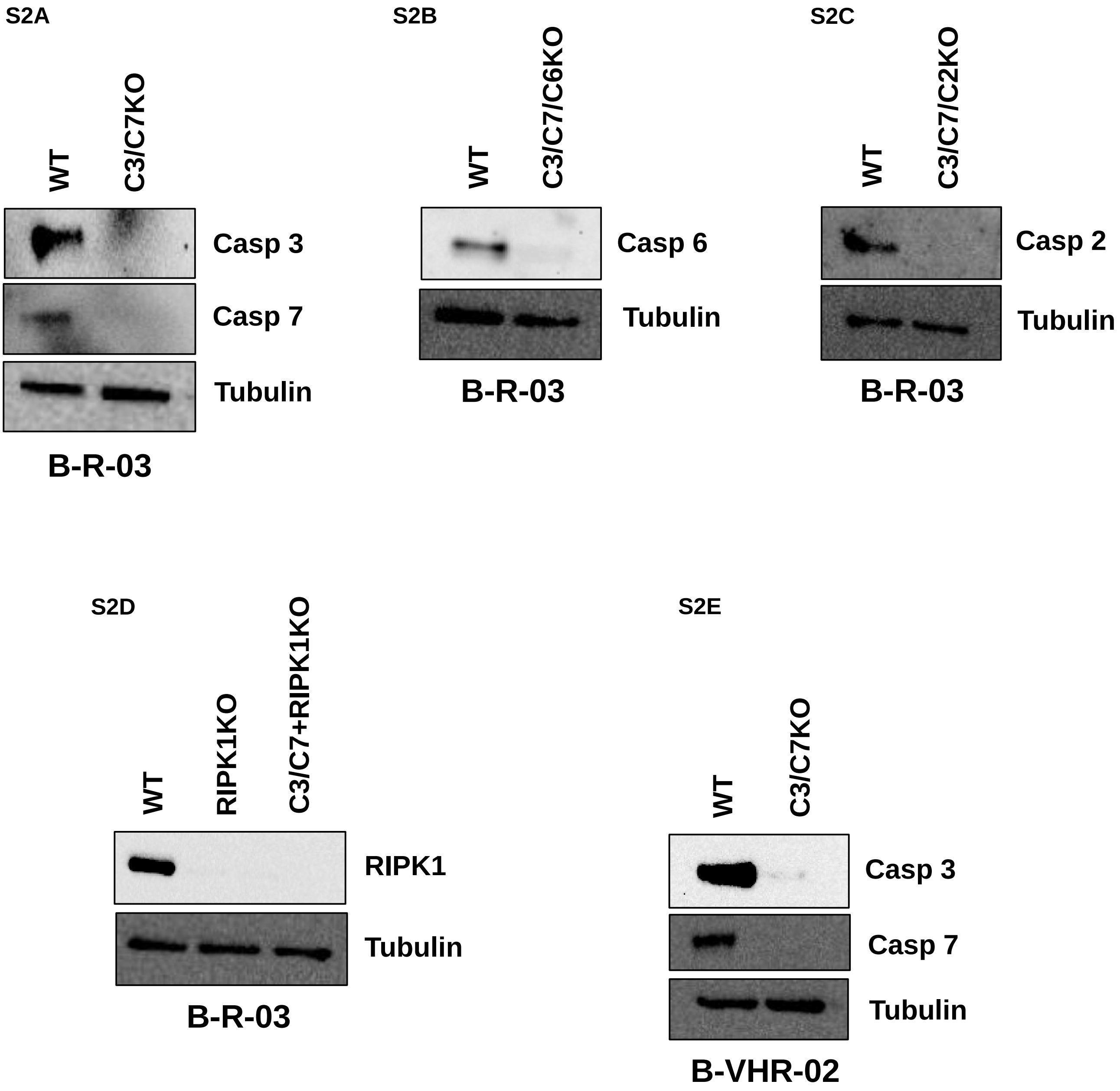

Figure S2: Validation of caspase and RIPK1 knockout. (**A**) Validation by western blot of caspases-3 and -7 knockout (C3/C7KO) in B-R-03 cells. (**B**) Validation by western blot of caspase-6 knockout. The sgRNA targeting caspase 6 was added in B-R-03 C3/C7KO cells in order to generate the triple knockout (C3/C7/C6KO). (**C**) Validation by western blot of caspase-2 knockout. The sgRNA targeting caspase 2 was added in B-R-03 C3/C7KO cells in order to generate the triple knockout (C3/C7/C2KO). (**D**) Validation by western blot of triple knockout, RIPK1 and caspases-3/ -7 KO (C3/C7+RIPK1KO). The sgRNA targeting RIPK1 was added in B-R-03 C3/C7KO cells. (**E**) Validation by western blot of caspases-3 and -7 knockout (C3/C7KO) in B-VHR-02 cells.

S3A

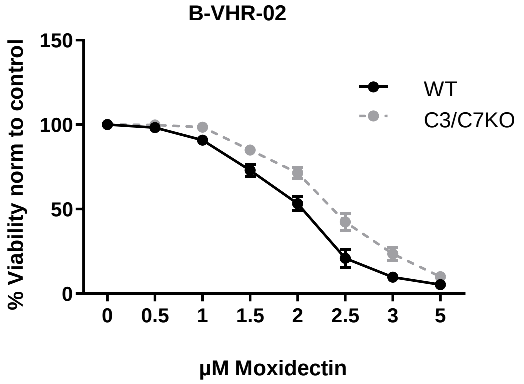

S3B

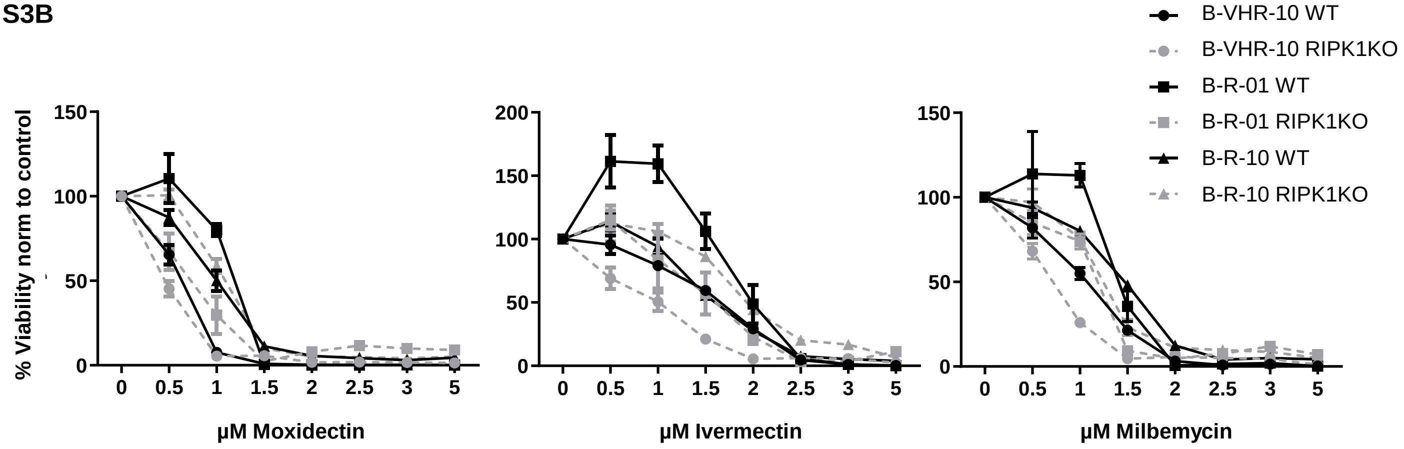

S3C

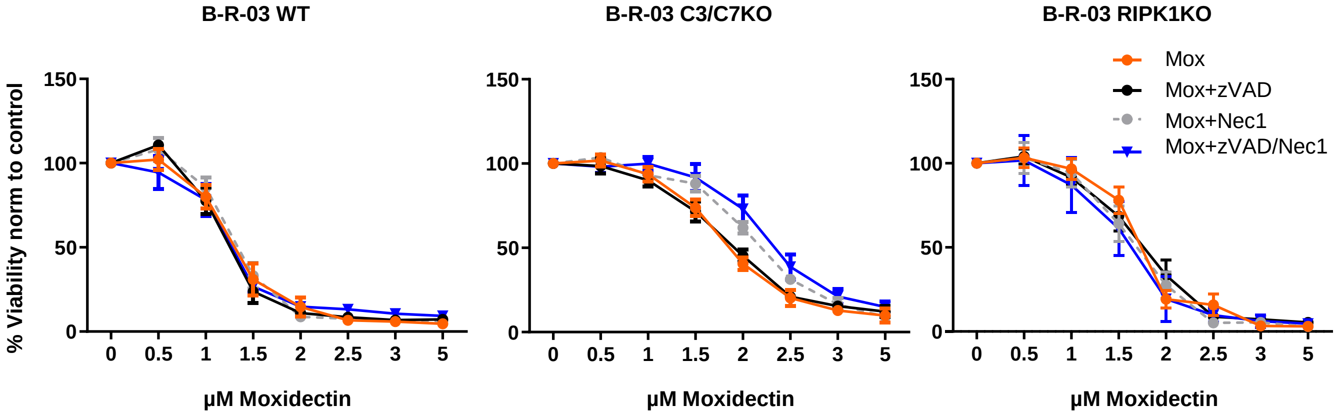

S3D

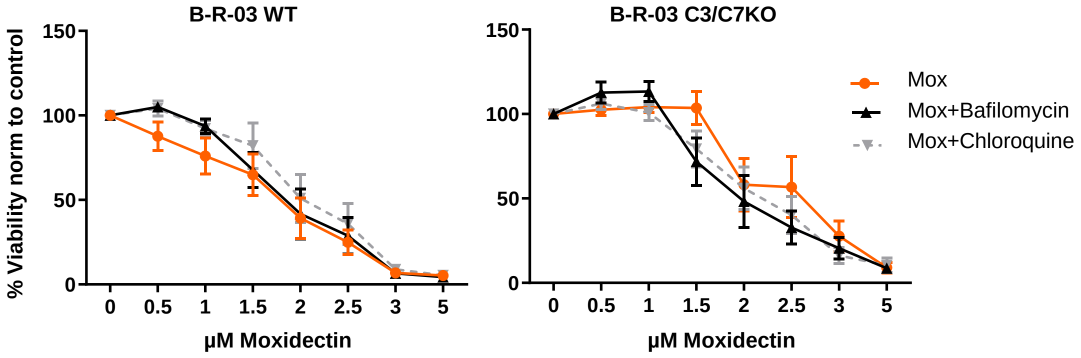

Figure S3: Anthelmintic agents induce cell death independent of caspases and RIPK1. Dose response curves of wild type (WT) and indicated gene knockout (KO) ALL are given. (**A**) Moxidectin induces cell death in WT (black), and Caspases-3 and -7 KO (C3/C7KO; gray) B-VHR-02. (**B**) Moxidectin, ivermectin, and milbemycin induce cell death in WT (B-VHR-10, B-R-01, and B-R-10, black continuous lines) and RIPK1 KO (RIPK1KO, gray dotted lines) ALL. (**C**) Moxidectin (Mox, oragnge) induces cell death in B-R-03 WT, C3/C7KO, and RIPK1KO, in presence of the apoptotic inhibitor z-VAD (25µM) (Mox+zVAD, black), or the necroptotic inhibitor Nec-1 (25µM) (Mox+Nec1, gray), either alone or in combination (Mox+zVAD/Nec-1, blue). (**D**) Moxidectin (Mox, orange) induces cell death in B-R-03 WT and C3/C7KO, in presence of the autophagy inhibitors bafilomycin (5nM) (Mox+Bafilomycin, black), and chloroquine (10µM) (Mox+Chloroquine, gray). All the dose response curves were normalized to vehicle control and performed in N=3 independent experiments. Quantifications represent mean±s.e.m.

S4A

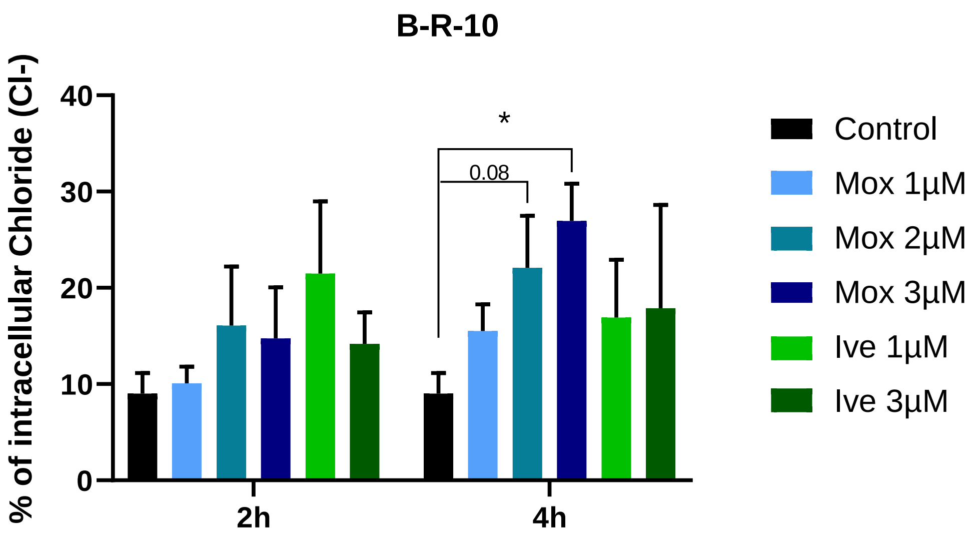

B-R-10
% of intracellular Chloride (Cl-)
40
30
20
10
0
2h
4h
*
0.08
Control
Mox 1µM
Mox 2µM
Mox 3µM
Ive 1µM
Ive 3µM

S4B

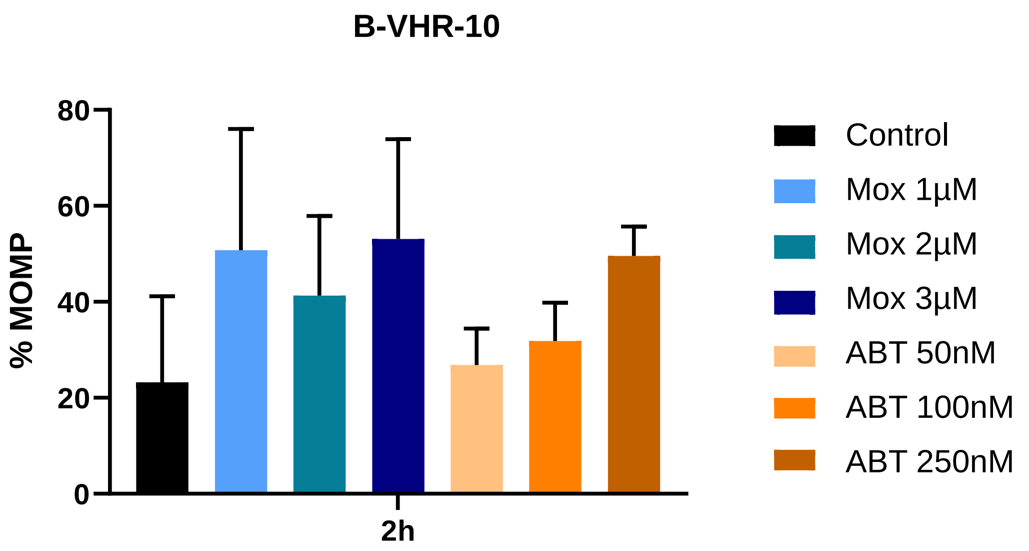

B-VHR-10
% MOMP
80
60
40
20
0
2h
Control
Mox 1µM
Mox 2µM
Mox 3µM
ABT 50nM
ABT 100nM
ABT 250nM

Figure S4: Moxidectin induces leukemia cell death by increasing intracellular chloride and inducing mitochondrial outer membrane permeabilization (MOMP). (**A**) Quantification of intracellular chloride in vehicle treated B-R-10 (black) compared to cells treated with moxidectin (Mox) 1µM (light blue), 2µM (blue) and 3µM (dark blue) or ivermectin (Ive) 1µM (light green), and 3µM (green) for 2 or 4 hour. Quantifications of N=3 independent experiments representing mean±s.e.m, Paired *t-test*, p-value 0.08; * p-value ≤0.015. (**B**) MOMP quantification in control B-VHR-10 (black) ALL cells compared to cells treated with moxidectin (Mox) 1µM (light blue), 2µM (blue) and 3µM (dark blue) or ABT-263 (ABT) 50nM (light pink), 100nM (orange), and 250nM (brown) for 2h. Quantifications of N=3 independent experiments representing mean±s.e.m. Paired *t-test*.

S5A

B-VHR-12

Z-Score 6.498

B-VHR-10

Z-Score 5.664

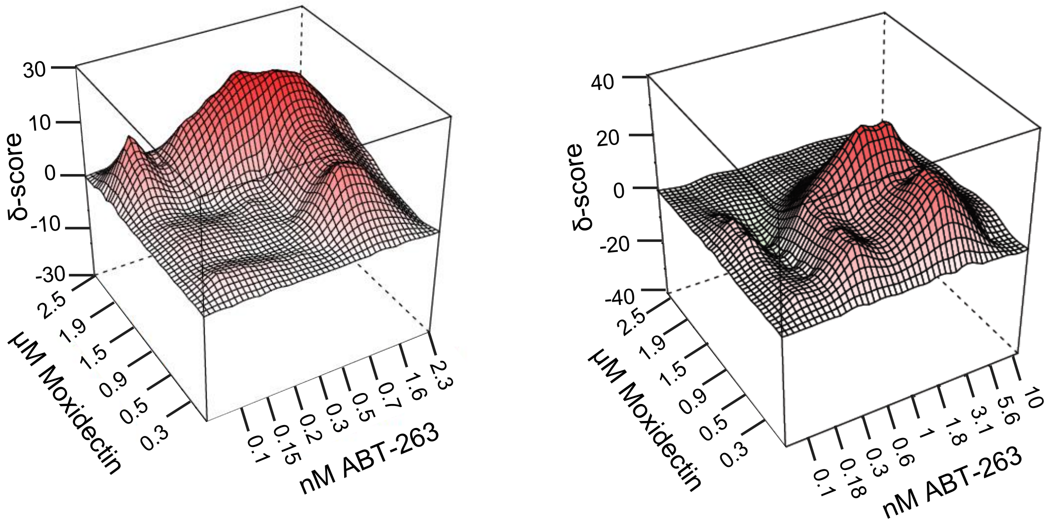

S5B

B-R-10

Z-Score 15.074

B-R-03

Z-Score 15.029

B-VHR-10

Z-Score 22.893

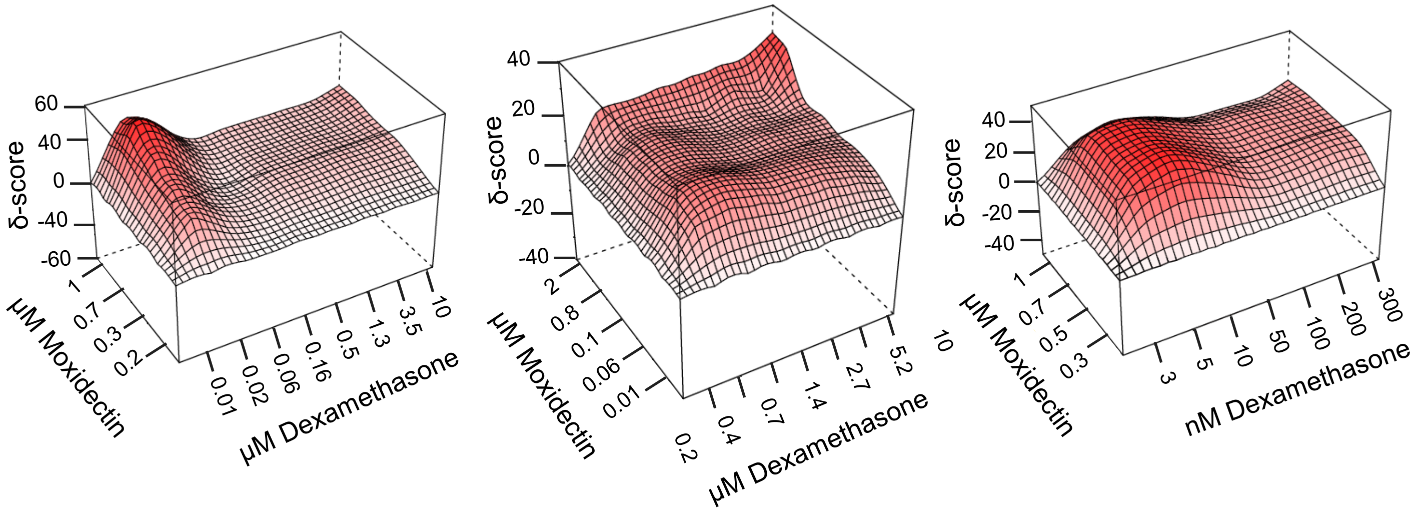

Figure S5: Synergistic activity of moxidectin with ABT-263 and dexamethasone. (**A**) 3D representation map of the calculated synergy between moxidectin and ABT-263 in B-VHR-12 (Z-Score= 6.498) and B-VHR-10 (Z-Score= 5.664) samples. (**B**) 3D representation map of the calculated synergy between moxidectin and dexamethasone in B-R-10 (Z-Score= 15.074), B-R-03 (Z-Score= 15.029), and B-VHR-10 (Z-Score= 22.893) samples.
